# Conductive Nanocomposite Hydrogels for Neural Tissue Engineering: A Systematic Scoping Review of Recent Trends

**DOI:** 10.1002/advs.202416085

**Published:** 2025-09-08

**Authors:** Mohammad Moghaddasi, Busra Oktay, Ayse Betul Bingol, Reyhan Yanikoglu, Meryem Muslu, Ibrahim T Ozbolat, Cem Bulent Ustundag

**Affiliations:** ^1^ Department of Bioengineering Yildiz Technical University Istanbul 34722 Turkey; ^2^ Department of Chemical Engineering Yildiz Technical University Istanbul 34210 Turkey; ^3^ Engineering Science and Mechanics Department Penn State University, University Park PA USA; ^4^ The Huck Institutes of the Life Sciences Penn State University, University Park PA USA; ^5^ Biomedical Engineering Department Penn State University, University Park PA USA; ^6^ Neurosurgery Department Penn State University, University Park PA USA; ^7^ Health Biotechnology Joint Research and Application Center of Excellence Istanbul 34210 Turkey

**Keywords:** conductive hydrogel, nanocomposite, nanomaterial, neural differentiation, neural tissue engineering

## Abstract

Conductive nanocomposite hydrogels (CNHs) represent a promising tool in neural tissue engineering, offering tailored electroactive microenvironments to address the complex challenges of neural repair. This systematic scoping review, conducted in accordance with PRISMA‐ScR guidelines, synthesizes recent advancements in CNH design, functionality, and therapeutic efficacy for central and peripheral nervous system (CNS and PNS) applications. The analysis of 125 studies reveals a growing emphasis on multifunctional materials, with carbon‐based nanomaterials (CNTs, graphene derivatives; 36.8%), metals (Iron oxides, gold, etc.; 24.0%), conductive polymers (PEDOT, PPy, etc.; 16.0%), and hybrid systems dominating due to their synergistic electrical, mechanical, and bioactive properties. For CNS repair, spinal cord injury models (*n*  =  42) leverage antioxidant‐conductive hybrids and immunomodulatory systems to mitigate oxidative stress and neuroinflammation. For PNS repair—particularly sciatic nerve regeneration (*n*  =  20)—CNHs demonstrate efficacy through stimuli‐responsive strategies (including wireless and self‐powered piezoelectric and magnetic systems) and biomimetic scaffold design to guide axonal regeneration. Tailored hydrogel designs also address traumatic brain injury, stroke, and Parkinson's disease. Beyond these, CNHs show promise in diverse neural tissue engineering contexts, including neurovascular niche reconstruction for diabetic wound healing, coordinated neurogenic and osteogenic differentiation in bone and muscle repair, and auditory neurogenesis in cochlear applications. This review highlights the potential of CNHs by elucidating recent applications across various neural tissue engineering contexts.

## Graphical abstract

This systematic scoping review explores conductive nanocomposite hydrogels for neural tissue engineering(NTE), focusing on composition, cellular interactions, and neural differentiation capabilities. The review synthesizes data from 125 studies, examining important features including commonly used materials, cell types, and differentiation strategies for a variety of neural regeneration applications. It provides an integrative analysis of key properties and methods, offering insights into their potential as therapeutic tools for NTE.

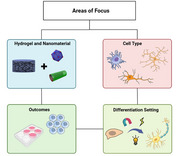

## 1 Review Methodology, Information Sources, and Study Selection

This systematic scoping review was conducted in accordance with the Preferred Reporting Items for Systematic Reviews and Meta‐Analyses Extension for Scoping Reviews (PRISMA‐ScR) guidelines.^[^ ^1^ ^]^ The review protocol was registered on the Open Science Framework (OSF) on September 26, 2024, prior to the initiation of the review process.^[^ ^2^ ^]^ Relevant literature published primarily between January 1, 2020, and December 31, 2024, was identified through comprehensive searches of PubMed (MEDLINE), Scopus, Web of Science (WoS), ScienceDirect, and Wiley databases using customized search terms (see Supplementary Files or OSF). Identified studies were imported into the Rayyan platform^[^ ^3^ ^]^ for deduplication and screening. Title and abstract screening were independently performed by multiple reviewers on Rayyan with a double‐blind approach. This was followed by full‐text screening, during which articles were assessed for eligibility based on predefined inclusion and exclusion criteria. Any disagreements were resolved through consultation with a third reviewer.

The inclusion and exclusion criteria were designed to select studies pertinent to the scope of this review. No restrictions were imposed on the methodology for hydrogel synthesis. Studies incorporating conductive nanostructures—such as nanoparticles, fibers, or sheets—were included and classified into certain subtypes based on material composition including carbon, metal, polymer, ceramic, or semiconductor. Moreover, studies involving human or animal cells were eligible if their aim was to create neural tissues or models. In vivo regeneration studies were also included, even in the absence of in vitro investigations. Additionally, neural differentiation studies were required to provide clear evidence of differentiation into neural or glial lineages, with details on induction methods (e.g., electrical stimulation or neurogenic media) and evaluations of differentiation outcomes, such as gene/protein analysis or morphological observations. Exclusion criteria were applied to eliminate studies lacking conductive nanostructures or cellular components, those unrelated to neural differentiation, non‐English publications, non‐original research formats (e.g., reviews), studies with inaccessible PDFs, or studies published outside the 2020–early 2025 timeframe.

To ensure consistency in data extraction, a pilot data extraction test was conducted using a random selection of 10 studies. These studies underwent double‐blind data extraction by the authors using the prepared extraction form, and the results were recorded in MS Excel (RRID: SCR_016137). Any discrepancies were addressed through a reconciliation process in consultation with a supervisor reviewer, C.U., who has expertise in neural tissue engineering and nanocomposites. This process led to further refinement of the data extraction form (see Supporting Information files or OSF). After the pilot test, data extraction for the 125 eligible studies was performed by blinded reviewers, with the extracted data recorded in MS Excel. Discrepancies were resolved with assistance from the supervisor reviewer (C.U.). Data cleaning and analysis were then conducted using pandas (RRID: SCR_018214) in Python, tidyverse (RRID: SCR_019186) in R, or MS Excel. Visualizations were created using ggplot2 in R (RRID: SCR_014601) or MS Excel. For access to the data and code notebooks, readers are referred to the OSF registration or Supplementary files.

**Figure 1:**
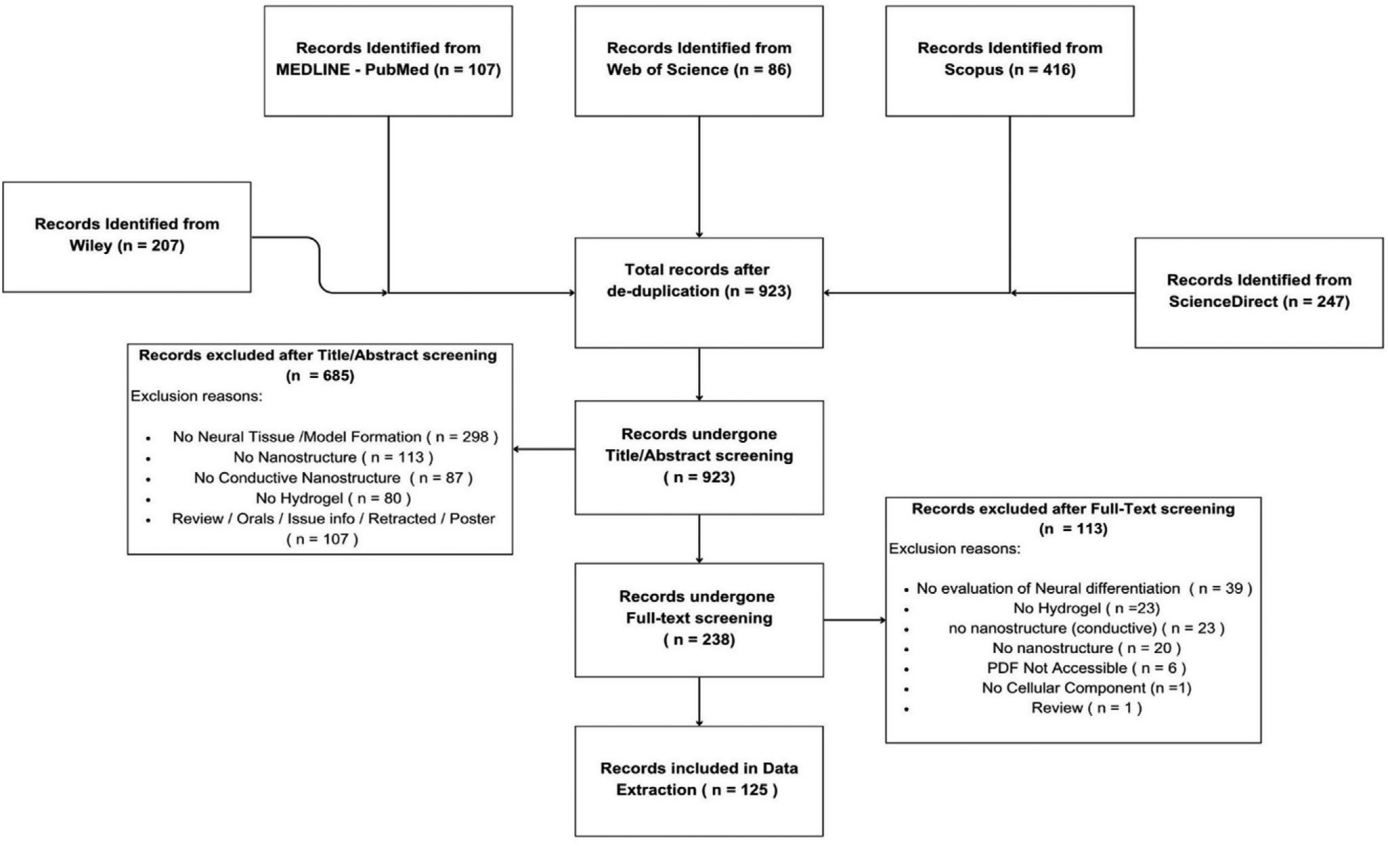
Study Screening Flow Diagram. Using customized search terms, search across Scopus (416), MEDLINE (107), WoS (86), Wiley (207), and ScienceDirect (247) electronic databases identified a total of 1063 articles. After deduplication, 923 papers remained and underwent title/abstract and full‐text screening using Rayyan, resulting in the exclusion of 685 and 113 articles, respectively, with reasons documented. The justifications for exclusion, along with the corresponding number of articles, are detailed for both stages. During data extraction, full‐text articles for six studies could not be accessed.

Our initial search identified 1063 studies (Scopus, *n* = 416; MEDLINE, *n* = 107; WoS, *n* = 86; ScienceDirect, *n* = 247; Wiley, *n* = 207), which were reduced to 923 after deduplication. Following title and abstract screening, 685 studies were deemed ineligible, with justifications provided. Full‐text screening excluded an additional 113 studies, with reasons detailed in **Figure** **1** or supplementary tables accessible via OSF. The reconciled data extraction results from the final 125 publications are available on OSF.

This systematic scoping review aims to summarize recent applications of conductive nanocomposite hydrogels in neural tissue engineering, with a focus on studies published in the past five years. The decision to approach this topic systematically was made to minimize bias and ensure high‐quality data analysis, resulting in reproducible evidence.^[^ ^1^ ^]^ To date, no review has offered a systematic analysis of this research area, specifically focusing on diverse application areas and outcome evaluation of cell‐laden nanocomposite hydrogels. In this review, we provide a broad evaluation of the critical aspects of neural tissue regeneration using conductive nanocomposite hydrogels. The research questions that guided the planning of this review were as follows:

- What are the sources and characteristics of the cells, biomaterials, and nanostructures used in the creation of conductive hydrogel models, and what techniques were employed for their synthesis and integration?
- What are the outcomes of using conductive hydrogels for the intended injury or application? If applicable, how was neural cell differentiation induced, and how were the outcomes assessed both in vitro and in vivo?

## 2 Overview of CNHs in Neural Tissue Engineering (NTE)

### 2.1 Introduction and Background

**Figure 2:**
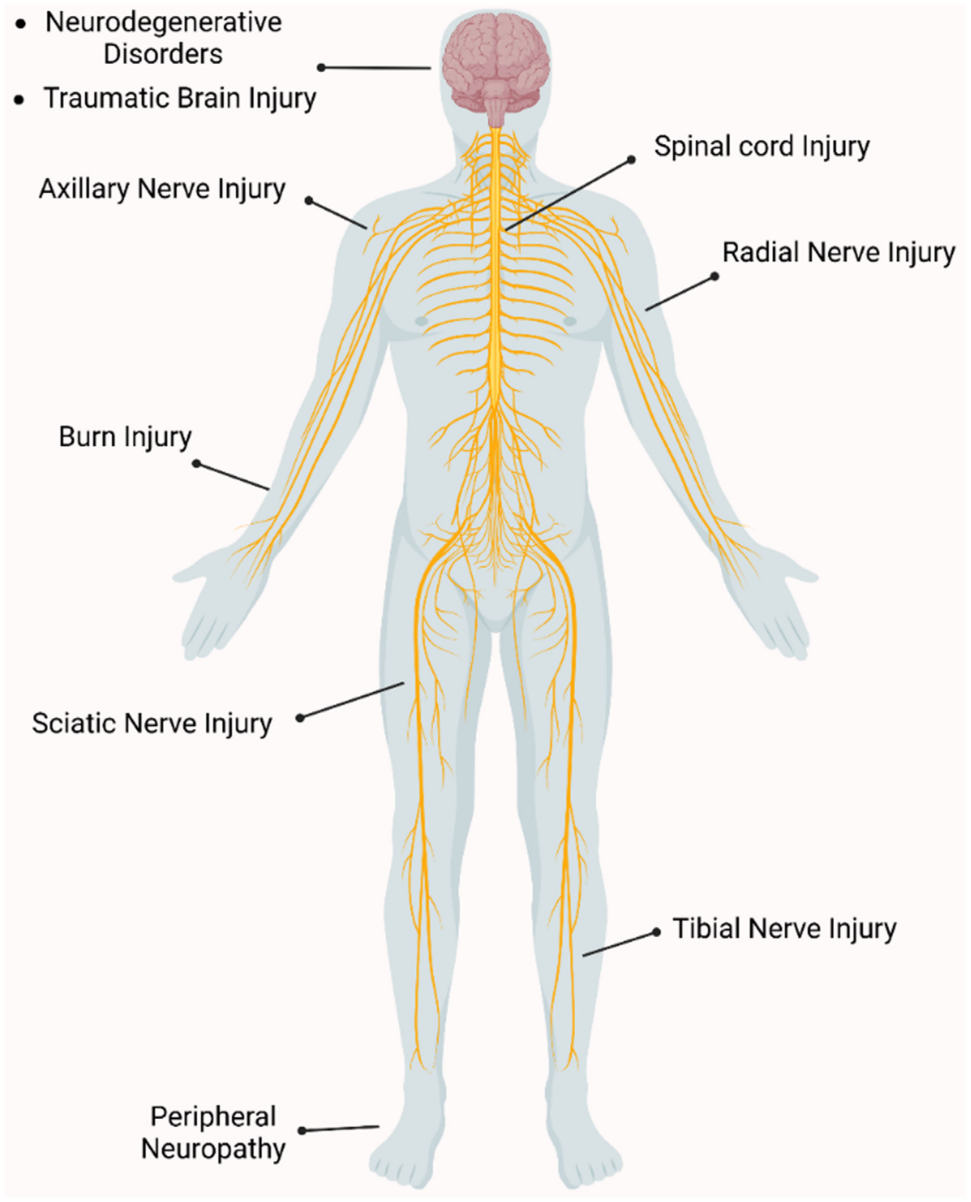
Instances of Central and Peripheral Nervous System Injuries. This illustration highlights the locations of various conditions affecting the central and peripheral nervous systems. Damage to the CNS, such as neurodegenerative disorders and traumatic brain injuries, impacts the brain and spinal cord, affecting movement, sensation, and cognition. PNS injuries, such as damage to the sciatic, tibial, and radial nerves, can lead to localized issues with movement and sensation. (Created in BioRender. mqdsi, M. 2025 https://BioRender.com/3r4wlnx).

Nervous tissue, which includes both the central and peripheral nervous systems (CNS/PNS), plays a crucial role in sensory, motor, and cognitive functions. However, it is highly susceptible to damage from trauma, neurodegenerative diseases (e.g., Alzheimer's disease), or metabolic disorders, leading to disruptions in the electrochemical signaling essential for neural communication **Figure** **2**.^[^ ^4^, ^5^ ^]^ Unlike many other tissues, neurons have limited regenerative capacity: in the CNS regeneration is largely impeded by inhibitory factors such as glial scarring, whereas the PNS retains greater regenerative potential because Schwann cells support axonal regrowth, albeit at a slower rate.^[^ ^6^ ^]^ Recent advancements in NTE have focused on the use of conductive biomaterials and electroactive scaffolds to replicate native signaling, promote axonal regrowth, and overcome barriers to regeneration, presenting promising strategies for functional recovery.^[^ ^7^ ^]^

**Figure 3:**
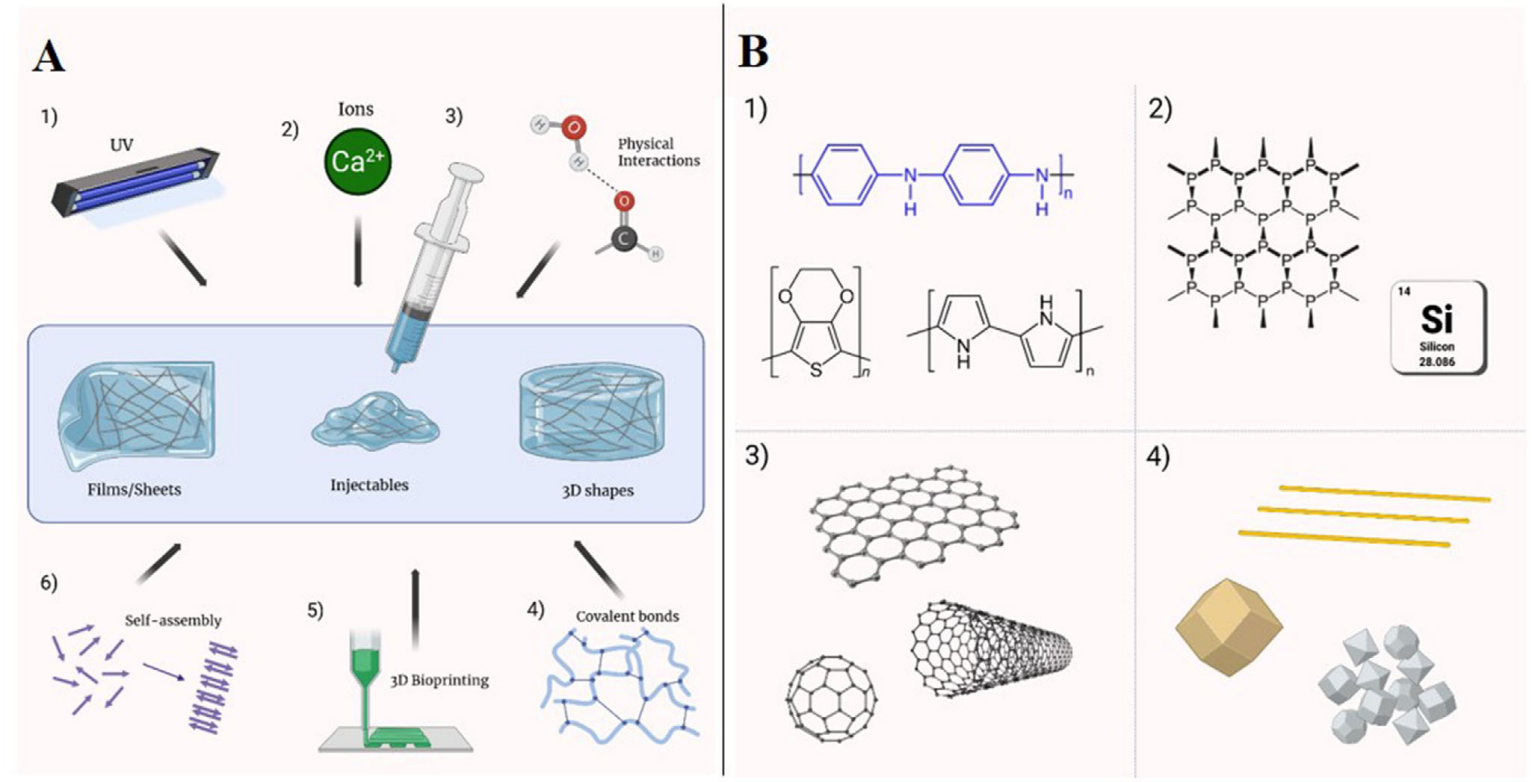
Hydrogel Synthesis Methods and Conductive Nanotypes. A) A variety of methods, including (1) Photopolymerization, (2) Ionic crosslinking, (3) Physical crosslinking, (4) Covalent crosslinking, (5) 3D bioprinting, and (6) Self‐assembly, are utilized to fabricate hydrogels in various shapes such as films/sheets, injectables, and general 3D shapes via molding (from left to right). (Created in BioRender. mqdsi, M. 2025 https://BioRender.com/brrzf94). B) Conductive nanomaterials can be categorized into four main groups: (1) Polymer‐based (e.g., PEDOT, Ppy, PANI), (2) Semiconductor‐based (e.g., black phosphorus, silicon‐based), (3) Carbon‐based (e.g., carbon nanotubes/nanodots, graphene), and (4) Metal‐based (e.g., gold, iron, platinum). Some materials may not fit neatly into the proposed classification, and the purpose was to categorize materials with similar composition and properties for clarity in comparison. (Created in BioRender. mqdsi, M. 2025 https://BioRender.com/y124pjt).

Hydrogels are interconnected polymer networks that play a crucial role in tissue engineering due to their high water content, biocompatibility, tunable mechanical properties, and stimuli‐responsive behavior.^[^ ^7^ ^]^ Various hydrogel fabrication techniques for biomedical applications have been extensively reported in the literature. Different crosslinking methods—including physical, chemical, ionic, and optical—are commonly employed, both in traditional and advanced techniques such as 3D bioprinting, to tailor hydrogel properties **Figure** **3**A.^[^ ^8^ ^]^ In NTE, hydrogels hold significant promise for neural regeneration and differentiation by providing physical guidance cues that support axonal growth, mimicking native ECM stiffness, and enabling the sustained release of neurotrophic factors.^[^ ^9^ ^]^ However, pristine hydrogels face limitations in NTE applications, such as low electrical conductivity, limited mechanical strength, and reduced bioactivity. Advances in nanotechnology have led to the development of nanocomposite hydrogels, which enhance bioactivity and electrical conductivity, making them particularly suitable for excitable tissues.^[^ ^10^ ^]^

From the late 1990s through the early 2000s, significant advancements were made in the production and characterization of nanocomposites. Around the same period, the incorporation of nanomaterials into hydrogels began to gain attention. Researchers realized that this combination could enhance the mechanical and functional properties of hydrogels, leading to the emergence of nanocomposite hydrogels.^[^ ^11^ ^]^ Since then, hydrogels have been explored for various applications, including tissue engineering, wound healing, drug delivery, and biosensing.^[^ ^12^ ^]^ In the field of NTE, numerous studies have incorporated different conductive nanomaterials into hydrogels and investigated their interactions with neural cells, which date back to the early 2000s.^[^ ^13^ ^]^ CNHs offer notable advantages over conventional pure hydrogels by incorporating electrically conductive nanomaterials such as graphene, carbon nanotubes, or conductive polymers into the hydrogel structure. This integration enables essential functions for neural repair that traditional hydrogels lacking electrical conductivity cannot achieve. By mimicking the electrochemical microenvironment of neural tissue, CNHs provide a conductive substrate that supports bioelectrical signal propagation, including action potentials and synaptic transmission, which are crucial for coordinating neuronal activity.^[^ ^14^ ^]^ The conductivity of CNHs allows for electrical stimulation (ES), which enhances neuronal differentiation, axon growth, and the release of neurotrophic factors, thereby accelerating regeneration in spinal cord injuries and peripheral nerve gaps.^[^ ^15^, ^16^ ^]^ Furthermore, incorporating nanomaterials of different dimensions into the hydrogel matrix introduces nanoscale topographical features, such as surface roughness and aligned fibrillar patterns, that help replicate the structural complexity of neural extracellular matrices.^[^ ^17^, ^18^ ^]^ These nanotopographical cues, combined with tunable mechanical properties modulated by nanomaterial concentration and characteristics, have the potential to synergistically enhance cellular responses critical for NTE applications.

### 2.2 Types of Conductive Materials Used in CNHs

The integration of conductive nanomaterials within hydrogel media equips the composite with higher electrical conductivity, providing a biomimetic environment that promotes neural differentiation by supporting endogenous signaling pathways.^[^ ^19^ ^]^ Conductive nanomaterials can be categorized into several groups Figure 3B; **Table** **1**, each offering distinct advantages for NTE.

**Table 1:** Comparative overview of conductive nanomaterials for NTE: classification by nanostructure type, properties, and applications.^[^ ^29^, ^30^, ^31^ ^]^

**Nanostructure Type**	**Examples**	**Advantages**	**Disadvantages**	**Applications**
**Carbon‐based**	CNTs: ‐ Single‐walled (SWCNTs)‐ Multi‐walled (MWCNTs) Graphene Derivatives: ‐ Graphene Nanoplatelets ‐ Graphene Oxide ‐ Reduced Graphene Oxide Carbon Quantum Dots (GQDs)	‐ High electrical conductivity ‐ Exceptional mechanical strength ‐ Large surface for functionalization ‐ Chemical stability	‐ Potential cytotoxicity (pristine forms) ‐ Aggregation tendency ‐ Batch‐to‐batch variability ‐ Processing challenges ‐ Unclear long‐term biodegradation ‐ Dispersion difficulties	‐ Neural stem cell differentiation ‐ Peripheral nerve conduits ‐ Neural electrodes ‐ Drug delivery systems
**Metal‐based**	Gold Nanoparticles (AuNPs) Silver Nanoparticles (AgNPs) Iron Oxide Nanoparticles: ‐ Magnetite (Fe_3_O_4_) ‐ Maghemite (γ‐Fe_2_O_3_) Platinum Nanoparticles	‐High electrical conductivity ‐ Biocompatibility ‐Magnetic responsiveness ‐ Antimicrobial properties ‐ Catalytic activity	‐ Non‐biodegradable accumulation ‐ Oxidation susceptibility (some metals) ‐ Size‐dependent toxicity ‐ Immune system activation	‐ Magnetic field‐guided delivery ‐ Antimicrobial implants ‐ Wireless electrical stimulation ‐ Neural electrode coatings ‐ Emerging photothermal therapy
**Ceramic‐based**	Barium Titanate (BaTiO_3_) Bismuth Ferrite (BiFeO_3_) Cobalt Ferrite (CoFe_2_O_4_) Titanium Dioxide (TiO_2_) Zinc Oxide (ZnO)	‐ Piezoelectric and self‐powered stimulation properties ‐ Mechanical‐electrical transduction ‐ Biocompatibility ‐ Photocatalytic activity	‐ Lower electrical conductivity ‐ Brittleness and limited flexibility ‐ Processing difficulties ‐ Complex synthesis requirements	‐ Self‐powered neural stimulators ‐ Bone‐neural interface materials ‐ Mechanically‐responsive scaffolds ‐ Bioactive neural conduits
**Polymer‐based**	Polypyrrole (PPy) Polyaniline (PANi) Polythiophene (PT) PEDOT/ PEDOT:PSS Poly(*p*‐phenylene vinylene) (PPV)	‐Tunable electrical conductivity ‐ Biocompatibility and Biodegradability ‐ Mechanical flexibility ‐ Easy processing and cost‐effective fabrication ‐ Electroactivity: Redox‐active properties	‐ Environmental stability and conductivity degradation issues ‐ Susceptibility to oxidation ‐ Poor cell adhesion in some types ‐ Doping agent leaching ‐ Processing solvent toxicity	‐ Neural prosthetics ‐ Biosensors ‐Tissue engineeringscaffolds ‐ Electrical stimulation devices
**Semiconductor‐based**	Black Phosphorus (BP) Germanium Phosphide (GeP) Silicon Phosphide (SiP) Molybdenum Disulfide (MoS_2_)	‐ Semiconducting behavior ‐ Biodegradability ‐ Tunable electronic properties ‐ Anisotropic properties: Direction‐dependent conductivity ‐ Photonic responsiveness	‐ Air/moisture sensitivity ‐ Limited stability ‐ Complex synthesis ‐ Batch variability ‐ Processing challenges ‐ Degradation rate control difficulties	‐ Photonic neural interfaces ‐ Neural sensors

Carbon‐based nanomaterials such as carbon nanotubes (CNTs), graphene oxide (GO), reduced graphene oxide (rGO), carbon dots, and graphene nanoplatelets are among the most widely used conductive nanostructures due to their excellent electrical conductivity, high mechanical strength, large surface area, and ease of surface modification. CNTs, available as single‐ or multi‐walled structures, are particularly effective at promoting neuronal growth and facilitating electrical stimulation, although their hydrophobic nature may necessitate surface modifications.^[^ ^20^ ^]^ GO and rGO—which are derived from graphene via oxidation and partial reduction, respectively— provide superior hydrophilicity and enhanced cell adhesion, promoting neurite outgrowth.^[^ ^21^ ^]^ Additionally, zero‐dimensional (0D) carbon nanostructures such as graphene quantum dots offer biocompatibility at low concentrations and can improve conductivity without significantly altering hydrogel mechanics, though higher concentrations may raise cytotoxicity concerns due to increased reactive oxygen species (ROS) production.^[^ ^22^ ^]^ Metal‐based nanomaterials including gold, silver, and iron oxide nanoparticles (IONPs) such as magnetite (Fe_3_O_4_) play a crucial role in NTE by offering superb electrical conductivity, antibacterial effects, and magnetic properties. These materials promote cell adhesion, neurite outgrowth, and neuronal marker expression, and can adjust cellular alignment when exposed to magnetic fields.^[^ ^23^ ^]^ Gold nanoparticles (AuNPs) enhance electrical conductivity and neuronal differentiation, while superparamagnetic iron oxide nanoparticles (SPIONs) enable magnetic field‐based guidance of neural growth and controlled delivery of neurogenic factors. Emerging materials, such as MXenes (e.g., Ti_3_C_2_Tx), also support neuronal maturation and network formation due to their biocompatibility.^[^ ^24^ ^]^

Research on conducting polymers began in the 1980s, and since then, studies have shown these materials to possess suitable biocompatibility, tunable conductivity, and mechanical flexibility, making them promising candidates for NTE. Polymer‐based nanomaterials, including conductive polymers such as polyaniline (PANI), polypyrrole (PPy), and poly(3,4‐ethylenedioxythiophene) (PEDOT), are increasingly utilized in NTE because of their tunable electrical properties, exhibiting electrical conductivity due to their conjugated structures.^[^ ^25^ ^]^ When embedded into hydrogels, these materials facilitate cellular interactions and have been shown to promote the differentiation of neural stem cells (NSCs) into neurons. Often, these conductive polymers are combined with synthetic polymers such as PEGDA to maintain scaffold integrity while enhancing electrical properties.^[^ ^26^ ^]^ Although traditional ceramic‐based nanomaterials are less conductive, they offer unique piezoelectric properties that convert mechanical stimulation (e.g., ultrasound) into localized electrical signals, supporting neural differentiation. Materials such as barium titanate (BaTiO_3_), bismuth ferrite (BiFeO_3_), and cobalt ferrite (CoFe_2_O_4_) are often used in composite systems—frequently combined with conductive polymers or magnetite—to achieve synergistic piezoelectric and magneto‐responsive effects.^[^ ^27^ ^]^ Lastly, semiconductor‐based nanomaterials, although less commonly adopted, have shown promise in neural tissue engineering. Phosphorus‐based 2D nanomaterials such as black phosphorus (BP), germanium phosphide (GeP), and silicon phosphide (SiP) are integrated as semiconductors to support neural tissue formation. BP, for instance, degrades into non‐toxic phosphate ions and has demonstrated potential in enhancing neural cell survival and differentiation.^[^ ^28^ ^]^

### 2.3 Neural Differentiation via CNHs

Neural differentiation is a complex process where neural stem or progenitor cells specialize into specific neuronal or glial lineages, playing a crucial role in the development and regeneration of the nervous system. This intricate biological event is governed by a sophisticated interaction between internal genetic programs and numerous external signals from the cellular microenvironment. These external factors, which include morphogens, cell‐to‐cell interactions, and signaling mediated by ECM, collectively impact epigenetic states and the expression of genes specific to certain lineages.^[^ ^32^ ^]^ In the realm of NTE, creating biomimetic scaffolds that can effectively direct and enhance neural differentiation is essential. CNHs represent a promising class of materials in this regard, as they are well‐suited to replicate the natural electrochemical environment of neural cells, thus aiding in ion transport and endogenous bioelectric signaling to influence lineage commitment.^[^ ^33^ ^]^ By incorporating conductive nanostructures into hydrogel matrices, CNHs provide a synergistic blend of topographical, electrical, and biochemical cues that are vital for fostering effective neural tissue differentiation.

The native electrical conductivity of nerve tissue, at 8 × 10^−4^ to 1.3 × 10^−3^ S cm^−1^,^[^ ^26^ ^]^ underscores the importance of an electroactive environment for neural development. CNHs contribute to this by replicating the electrical behavior of host tissue, facilitating optimal ion and electron conduction.^[^ ^34^ ^]^ The electrical conductivity by CNHs, either individually or in conjunction with exogenous ES, is a key factor in neural differentiation. They allow direct electrical signaling and modulation of membrane potential by creating a conductive path that simulates natural bioelectrical signals. Such a direct electrical connection alters the resting membrane potential of neural stem cells or progenitors, leading to the activation of voltage‐gated ion channels. The resulting ion influx, especially of Ca^2+^, serves as a key second messenger, which deploys intracellular signal transduction cascades that promote neural differentiation events such as neurite growth and expression of neuronal genes.^[^ ^35^ ^]^ Moreover, the combination of ES with conductive scaffolds can enhance neurogenic outcomes. ES has emerged as an influential method for promoting neural differentiation in various cell types through multiple mechanisms. These include the activation of intracellular signaling pathways, such as PI3K/Akt and MAPK/ERK, along with the stimulation of specific ion channels (e.g., SCN1A and CACNA1C), which are key to its effects.^[^ ^36^ ^]^ Coupling between mechanical and electrical signals is also emphasized by mechanosensitive channels like PIEZO2, where contact between neural cells and conductive hydrogel matrix can create mechanical stresses that open these channels to convert mechanical stimuli into electrical signals that drive differentiation.^[^ ^37^ ^]^

Topography is a critical regulator of intracellular signaling, cell growth, and lineage‐specific differentiation. The incorporation of nanostructures into hydrogel matrices introduces nanotopographical cues that increase surface area, roughness, and spatial patterning.^[^ ^38^ ^]^ These features enhance cell‐matrix interactions by promoting the adsorption of ECM proteins (e.g., laminin, fibronectin) containing integrin‐binding motifs. Neural cells recognize these motifs via transmembrane receptors, which activate adhesion complexes and cytoskeletal remodeling to guide neurite outgrowth and directional migration. Aligned nanostructures, for instance, mimic the anisotropic organization of native neural ECM, providing contact guidance cues that polarize growth cones and accelerate axon elongation.^[^ ^39^, ^40^ ^]^ Moreover, nanotopography can influence mechanotransduction by modifying local stiffness gradients and force transmission. For example, nanoscale grooves or ridges induce nuclear deformation, triggering YAP/TAZ signaling, governing transcriptional programs for neuronal differentiation.^[^ ^41^ ^]^ Concurrently, nanotopography activates focal adhesion kinase to increase pro‐neuronal gene expression.^[^ ^42^ ^]^ Also, nanoscale disordered topographies with feature sizes well below 100 nm have been found to accelerate electrophysiological maturation of neurons, consistent with enhanced function of voltage‐gated ion channels.^[^ ^43^ ^]^

Integrating nanomaterials into hydrogel networks enhances their strength through supplementary physical or chemical interactions, which allow for precise tuning of the elastic modulus to the stiffness of native neural tissue. Furthermore, neural cells require dynamic microenvironments that allow remodeling during processes of neurite extension. Nanomaterials are able to deliver viscoelastic behavior and rapid stress relaxation by reversible physical interactions or dynamic covalent bonds. This property mimics the dissipative character of neural ECM energy, relaxing cytoskeletal tension, preventing cell death, and stimulating neurite outgrowth through allowing the hydrogel to relax cellular forces.^[^ ^44^, ^45^ ^]^ Nanomaterials also endow CNHs with a variety of functionalities required for neural tissue engineering. They include surface functionalization with bioactive molecules (e.g., growth factors and cell‐adhesion peptides) to mimic neural ECM signaling in order to facilitate cell attachment and guide differentiation.^[^ ^46^ ^]^ Besides, nanomaterials have the ability to render CNHs stimulus‐responsive: magnetic nanoparticles aid scaffold orientation or site‐directed drug delivery by magnetic fields, and thermo‐/photosensitive nanomaterials are capable of facilitating on‐demand cargo release or scaffold remodeling by ultrasound or light.^[^ ^47^ ^]^ The combination of these multifactorial cues in synergistic ways—tunable stiffness gradients, electrically conductive, and surface chemistry—allows the creation of flexible platforms adjustable to various neural regeneration processes.

## 3 Results

### 3.1 Distribution Analysis of Key Parameters

**Figure 4:**
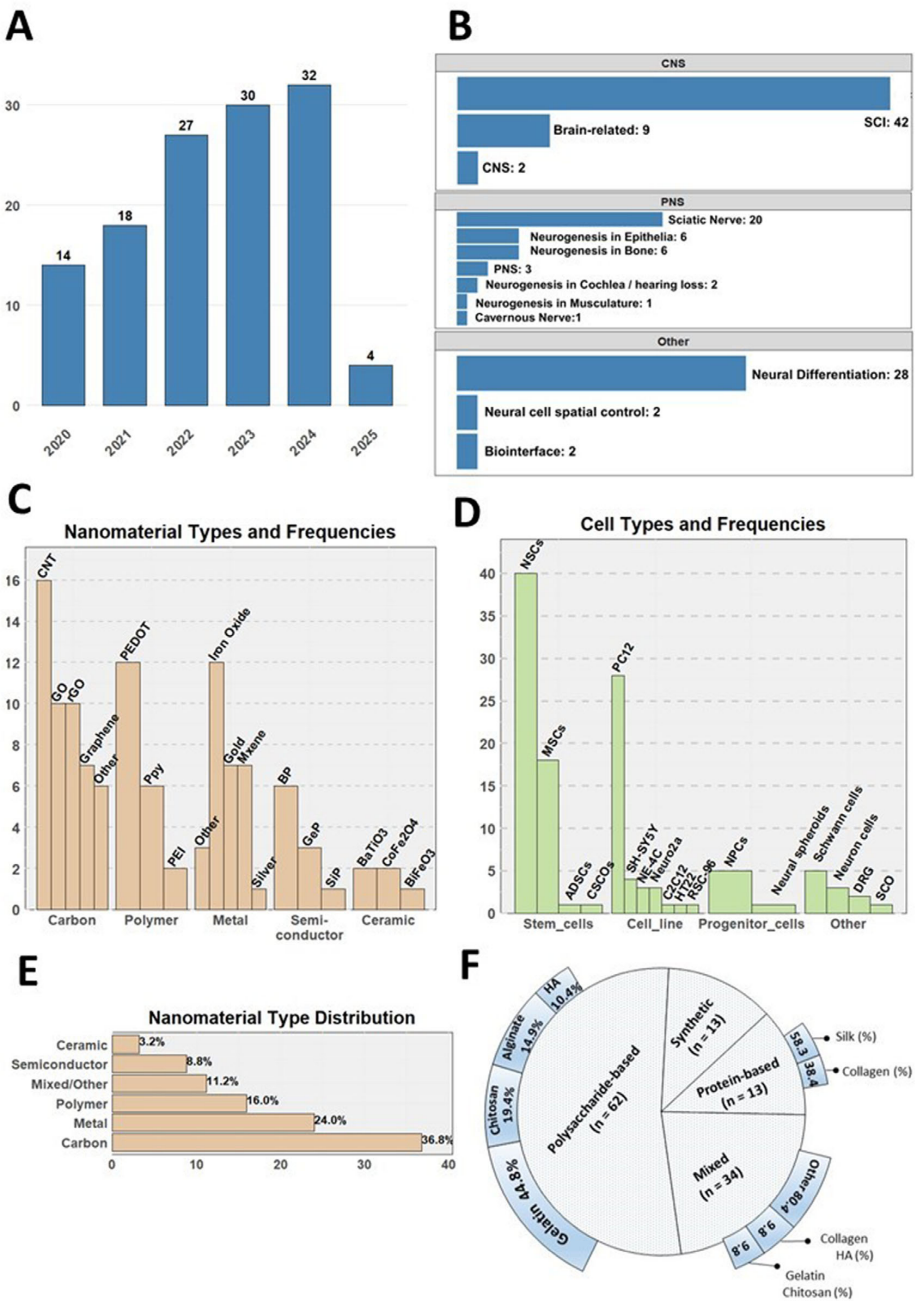
Summary of the Distribution of Key Parameters Based on A) publication year, B) target application, C,E) utilized nanomaterials [Carbon‐based = Other: g‐C_3_N_4_, Carbon quantum dots, Carbon nanofibers; Polymer‐based = PEI: Polyethylenimine; Metal‐based = Other: MoS_2_, FeCl3, MgMn‐based layered double hydroxides; Semiconductor‐based = BP: Black Phosphorus, GeP: Germanium Phosphide, SiP: Silicon Phosphide; Ceramic‐based = BaTiO3: Barium Titanate, CoFe2O4: Cobalt Ferrite, BiFeO3: Bismuth Ferrite] D) type of cell seeded [ADSCs: Adipose‐Derived Stem Cells, CSCOs: Cochlear Stem Cells Organoids; Cell_line = PC12: Rat Pheochromocytoma Cells, SH‐SY5Y: Human Neuroblastoma Cells, NE‐4C: Mouse Neural Stem Cells, Neuro2a: Mouse Neuroblastoma Cells, C2C12: Mouse Myoblast Cells, HT22: Mouse Hippocampal Cells, RSC‐96: Rat Schwann Cells; Progenitor_cells = NPCs: Neural Progenitor Cells; Other: SCO: Spinal Cord Organotypic Culture] and F) type of biomaterials used as the hydrogel backbone.

The distribution of key parameters is outlined across panels A–F of **Figure** **4**. This review includes studies published between 2020 and early 2025, highlighting recent advancements in conductive nanocomposite hydrogels for neural tissue engineering. The distribution of studies over these years is as follows: 14 in 2020, 18 in 2021, 27 in 2022, 30 in 2023, 32 in 2024, and 4 in 2025 (panel A). In terms of target applications, most studies focus on CNS, particularly spinal cord injury (SCI, *n* = 42). Additional CNS‐related research includes applications targeting the brain (*n* = 9) and general CNS contexts (*n* = 2). For PNS, the sciatic nerve is the most frequently studied target (*n* = 20), followed by neurogenesis in epithelial structures (*n* = 6) and bone (*n* = 6). Other applications, such as cochlear/hearing loss, musculature, and cavernous nerve regeneration, each appeared in 1–2 studies. Under the “Other” category, neural differentiation (*n* = 28) emerged as a key area of interest, while topics such as neural cell spatiometric control and biointerfaces were explored in fewer studies (each *n* = 2).

Cells seeded onto the hydrogels were predominantly rodent‐derived stem cells and cell lines, which is expected given their widespread use in neural tissue modeling due to their physiological relevance to humans, ease of cultivation, ethical considerations, and broad applicability.^[^ ^47^ ^]^ Among stem cells, neural stem cells (NSCs; *n* = 40) were the most frequently used, followed by mesenchymal stem cells (MSCs, *n* = 18). In the cell line category, PC12 cells (*n* = 28) were the most commonly studied, while SH‐SY5Y, NE‐4C, and Neuro‐2a were utilized to a lesser extent. Other less frequently observed cell types included neural progenitor cells (*n* = 5) and Schwann cells (*n* = 5), collectively accounting for 8% of the total studies. Additionally, primary neuron cells (*n* = 3) and dorsal root ganglion (DRG) cells (*n* = 2) were occasionally reported.

In the distribution of nanomaterial types, carbon‐based nanomaterials (*n* = 46) were the most frequently used, accounting for 36.8% of the studies. Among them, CNTs (*n* = 16), GO (*n* = 10), rGO (*n* = 10), and graphene (*n* = 7) comprised the majority. The preference for graphene derivatives is likely due to their superior hydrophilicity and ease of functionalization, enabled by the presence of oxygen‐containing functional groups.^[^ ^48^ ^]^ The next commonly used standalone nanomaterials were metals (24.0%) and polymers (16.0%). Among metal‐based nanomaterials, iron oxide (*n* = 12), gold (*n* = 7), and MXene (*n* = 7) were the most prevalent. In the polymer category, PEDOT (*n* = 12) and polypyrrole (*n* = 6) were frequently utilized due to their inherent electroconductive properties. Less commonly used nanomaterials included semiconductors (8.0%) and ceramics (4.0%). Notably, ceramic nanoparticles were often integrated with more conductive nanomaterials, such as electroconductive polymers or magnetite, to develop magnetoelectric composites. These hybrid structures leverage both piezoelectric and magneto‐responsive properties, enabling the generation of electrical charges when exposed to an external magnetic field.^[^ ^27^ ^]^

Lastly, panel F illustrates the distribution of material types used in the backbone of hydrogels, with subcategories detailed around the main pie chart. Standalone natural‐based compounds were utilized in nearly 61.4% of the studies, while purely synthetic materials were reported in only 10.6% of cases. More frequently, synthetic materials were combined with natural ones, forming composite natural‐synthetic blends that helped address challenges related to biocompatibility, degradation products, and the lack of natural adhesion sites.^[^ ^49^ ^]^ Among natural materials, polysaccharides were the most prevalent (*n* = 62), with gelatin (44.8%), chitosan (19.4%), alginate (14.9%), and hyaluronic acid (HA, 10.4%) being the most commonly used. Protein‐based materials (*n* = 13) appeared less frequently, with silk and collagen accounting for nearly all instances in this category. Lastly, the mixed category (*n* = 34) included studies utilizing multiple material types—either combinations of natural compounds, synthetic compounds, or both. Among these, collagen/HA (9.8%) and chitosan/gelatin (9.8%) were the most commonly observed formulations.

### 3.2 CNHs for CNS Applications

#### 3.2.1 SCI Repair Strategies

The regeneration of spinal cord tissue demands biomaterials that replicate the electroactive neural niche while dynamically addressing inflammation, oxidative stress, and mechanical mismatch.^[^ ^50^ ^]^ Recent advances in conductive nanocomposite hydrogels emphasize multifunctionality – merging key properties including electrical conductivity, immunomodulation, and antioxidant effects – to overcome the challenges of SCI repair (**Table** **2**). Conductive‐antioxidant hybrids counter oxidative stress while restoring bioelectrical signaling. Wu et al. engineered a collagen‐hyaluronan methacrylate hybrid hydrogel integrated with difunctional polypyrrole (PPy) nanoparticles to create a synergistic antioxidant and conductive environment for improved spinal cord injury repair. The antioxidant effect was primarily facilitated by the redox activity of PPy NPs, which efficiently scavenged DPPH radicals in a concentration‐dependent manner. The synergistic microenvironment effectively promoted neural differentiation of BMSCs, as indicated by the upregulation of neuron‐associated genes/proteins and increased expression of L‐VGCCs, further augmented by electrical stimulation.^[^ ^56^ ^]^ Furthermore, a MXene‐Au (MAu)‐embedded GelMA nanocomposite hydrogel combined with electrical stimulation exhibited multifunctional efficacy in SCI repair. Antioxidant functionality was facilitated via MXene's electron‐transfer capabilities, enabling effective ROS scavenging. MAu‐GelMA demonstrated over 80% elimination of DPPH radicals in vitro and significantly reduced in vivo ROS levels (DHE staining) at injury sites. This ROS‐scavenging ability lessened oxidative damage and inhibited secondary inflammation, as evidenced by reduced IL‐6⁺ cell counts.^[^ ^57^ ^]^ In additional research by Zhang et al., an injectable silk fibroin/black phosphorus/gallic acid (SF/BP/GA) nanocomposite conductive hydrogel exhibited antioxidant capabilities, attributed to BP's neuroprotective effects against ROS, while GA contributed anti‐inflammatory and pro‐myelination properties.^[^ ^53^ ^]^ Lastly, in a molybdenum disulfide/graphene oxide/polyvinyl alcohol (MoS_2_/GO/PVA) nanocomposite hydrogel developed by Chen et al., antioxidant properties were achieved through ROS scavenging by MoS_2_/GO nanosheets, reducing cellular ROS in NE‐4C and RAW264.7 cells and modulating cytokines.^[^ ^52^ ^]^

Immunomodulatory‐conductive systems integrate electroactivity with immune regulation to reshape the injury microenvironment. Zhang et al utilized a dual‐functional Gl‐C/W40 hydrogel scaffold—composed of CNTs, and the anti‐inflammatory compound wogonin (Wog)—significantly enhancing SCI recovery by synergizing electroconductivity with immunomodulation. The sustained release of Wog critically shifted macrophage polarization toward regenerative phenotypes, attributable to Wog's suppression of inflammation and neural cell apoptosis via the STAT3 pathway.^[^ ^63^ ^]^ Similarly, a hydrogel synthesized from diacerein‐terminated 4‐armed polyethylene glycol and graphene oxide exhibited a synergistic, sustained anti‐inflammatory effect alongside electroconductivity for the repair of spinal cord injuries. In vivo, this combination facilitated a pro‐regenerative microenvironment, as evidenced by the reduction in lesion cavities and enhanced neural regeneration.^[^ ^62^ ^]^ Lastly, an injectable, conductive GOMW hydrogel, composed of GelMA, oxidized dextran, and MoS_2_, demonstrated microenvironment responsiveness for SCI repair. Its acid‐sensitive release of Wnt5a at the injury site drove potent anti‐inflammatory effects, shifting macrophage polarization in vitro and in vivo toward the regenerative M2 phenotype.^[^ ^72^ ^]^

**Figure 5:**
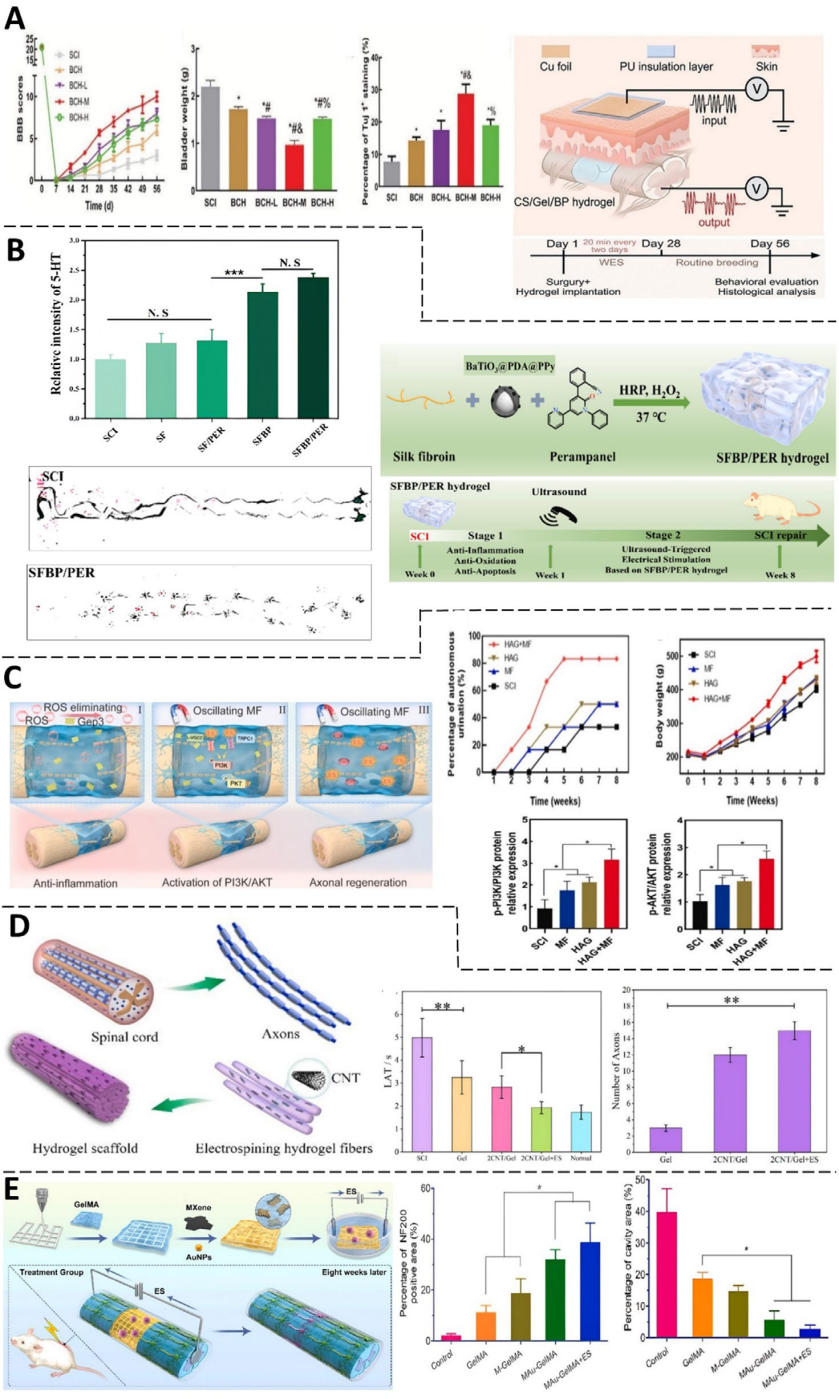
Application of CNHs and Electrical Stimulation for SCI. A) Schematic and timeline of in vivo wireless electrical stimulation via CS/Gel/BP hydrogel. Diagrams from left to right show:  BBB scores, bladder weights, and Tuj1 staining of rats in each group. (L,M,H refer to low, medium, and high ES doses, respectively) (Adapted with permission.^[^ ^71^ ^]^ Copyright 2025, JOHN WILEY AND SONS) B) Fabrication process of SFBP/PER hydrogel for spinal cord injury repair. Bottom: Footprint analysis at 8 weeks post‐surgery, with forepaws marked in red and hind paws in black. Top: Mean fluorescence density of tyrosine hydroxylase. (Adapted with permission.^[^ ^65^ ^]^ Copyright 2025, ELSEVIER).  C) HA/GeP3 hydrogel with wireless in situ stimulation promotes spinal cord regeneration via PI3K/AKT activation. Bottom: p‐PI3K/PI3K and p‐AKT/AKT expression. Top: Animal weight monitoring and independent urination rates. (Adapted with permission.^[^ ^64^ ^]^ Copyright 2025, ELSEVIER) D) Schematic of CNT/GelMA hydrogel fiber preparation inspired by spinal cord and axons. Left: Pain perception assessment via thermal pain test. Right: Axon count analysis 8 weeks post‐surgery. (Reproduced under the terms of the CC BY 4.0 license.^[^ ^58^ ^]^)  E) A multifunctional hydrogel with MXene‐Au composite and NSCs, combined with electrical stimulation, was developed to enhance motor recovery after SCI in rats. Left: NF200‐positive area percentage. Right: Cavity area percentage after 8 weeks.   (Adapted with permission.^[^ ^57^ ^]^ Copyright 2025, ELSEVIER).

Lastly, recent advances have harnessed wireless‐enabled hydrogel systems to deliver on‐demand ES without invasive hardware, markedly improving neural repair outcomes. Li et al. developed an SFBP/PER piezoelectric hydrogel, integrating Perampanel (PER), BaTiO3, and PPy, that generated ultrasound triggered electrical stimulation (1.0 MHz, 1.0 W cm^−^^2^) without implanted electrodes, thereby reducing infection risk and surgical complexity^[^ ^65^ ^]^ **Figure** **5**B. By combining the hydrogel's conductivity with PER‐mediated AMPA receptor antagonism, this platform modulated inflammation, accelerated motor recovery (improved BBB scores from week 2, longer stride length, restored hindpaw–forepaw coordination), and promoted structural repair via daily 15 min treatments. Building on magnetic actuation, an injectable HA/GeP_3_ hydrogel leveraged a 3 mT, 30 kHz field (20 min every 2 d) to stimulate NSC proliferation and migration via epithelial‐mesenchymal transition activation, yielding enhanced BBB scores, normalized weight, and restored spontaneous urination in spinal cord‐injured rats^[^ ^64^ ^]^ (Figure 5C). Similarly,  Liu et al. integrated BP into a conductive hydrogel that, when subjected to rotating magnetic fields (15 min of exposure every 12 h), established an electroconductive microenvironment which, within 3 days following injury, mitigated neuroinflammation and stimulated the PI3K/Akt signaling pathway to guide endogenous NSC toward mature neuronal phenotypes (23% enhancement in ChAT⁺/NF200⁺) and promote synaptic formation.^[^ ^51^ ^]^ Lastly, Wu et al. engineered a capacitive‐coupling‐responsive CS/Gel/BP scaffold that acted as a wireless receptor electrode, coupled with an external transmitter, thereby facilitating the generation of controllable in situ currents^[^ ^71^ ^]^ (Figure 5A). The incorporation of polydopamine‐coated BP nanosheets imparted stability, diminished impedance, stimulated calcium‐dependent signaling pathways, and expedited regeneration in spinal cord injuries. Complementing these wireless platforms, electrode dependent conductive scaffolds continued to refine NTE. Yao et al. fabricated aligned CNT/GelMA hydrogel fibers via electrospinning, which under applied ES, enhanced PC12 cell elongation, proliferation, and NSC differentiation. In vivo, the fibers reduced inflammation and enabled robust axonal regeneration across spinal cord lesions and remyelination, significantly improving functional recovery in rat SCI models^[^ ^58^ ^]^ (Figure 5D). Moreover, Agarwal et al. produced a graphene‐crosslinked collagen conduit by cryogelation (3.93 mS cm^−1^ at 0.5% w/v graphene) that maintained high porosity, and when stimulated (0.20 V mm^−1^, 5 min) drove upregulation of neuronal markers (MAP2, βtubulin III, Nestin) in BMMSCs. This conduit also polarized RAW 264.7 macrophages toward an anti‐inflammatory M2 phenotype.^[^ ^54^ ^]^

**Table 2:** Summary of studies utilizing CNHs for SCI repair, detailing the materials used, fabrication techniques, conductivity, differentiation/ES settings, and type of cells. (Note: Abbreviations are explained in Sections S1.1 in the Supporting Information).

**Hydrogel Backbone**	**Nano‐material (Dimension)**	**Fabrication and Integration Method**	**Highest Conductivity [S cm^−1^]**	**Differentiation Strategy**	**ES Setting**	**Type of Cell**	**Key Outcomes**	**Ref**.
Chitosan/ silk	Black phosphorus (2D)	Covalent crosslinking ‐ OP1	2.70 ×10^−2^	C + E	**MWS** (**Parameters not reported** **Duration**: 5 h over 10 d)	Stem cells	The BP Hydrogel enhanced synaptic regeneration and functional recovery in spinal cord injury models, while RMF further amplified its anti‐inflammatory effects by suppressing microglial activation.	[51]
PVA	GO/ MoS_2_ (0D, 2D)	physical crosslinking ‐ OP1	2.10 × 10^−3^	C	—	Stem cells (NSCs)	The MoS2GOPVA hydrogel reduced ROS levels, promoted NSCs neuronal differentiation while inhibiting glial cells, and improved locomotor function in SCI mice.	[52]
Silk	Black phosphorus (2D)	Physical crosslinking/ PPM ‐ OP1	4.00 × 10^−3^	C+N	—	Stem cells (NSCs)	The SFBPGA hydrogel increased NeuN‐positive neurons at the injury site, reduced ROS levels, and minimized fibrous scar formation. Basso Mouse Scale scores showed improved locomotion in SCI mice.	[53]
Collagen	Graphene (2D)	Covalent crosslinking ‐ OP1	4.00 × 10^−3^	C+E	**DCS** [**EFS**: 2000 mV cm^−1^ **Duration**: 0.6 h over 7 d]	Stem cells (BM‐MSCs)	Collagen‐graphene cryogels enhanced BM‐MSC proliferation under inflammatory conditions and promoted kynurenine secretion, aiding immune suppression post‐SCI. Histological analysis showed spinal cord infiltration into the graphene cryogel pores.	[54]
HA/ Gelatin	Au nanorods (1D)	Extrusion bioprinting ‐ OP1	1.30 × 10^−5^	C	—	Stem cells (NSCs)	The gold nanorod hydrogel exhibited high electrical conductivity, promoting neural regeneration. It was nontoxic, injectable, and paste‐like, able to provide safe implantation and better retention at the injury site.	[55]
Collagen/HA	Ppy (0D)	Self‐assembly/ PPM ‐ OP1	1.80 × 10^−3^	C+N+E	**DCS** [**EFS**: 100 mV cm^−1^ **Duration**: 4 h over 4 d]	Stem cells (MSCs)	The integration of polypyrrole into the hydrogel matrix conferred both conductive and antioxidative properties, establishing a conducive microenvironment for BMSCs and enhancing their therapeutic potential in spinal cord injury repair.	[56]
Gelatin	AuNPs, MXene (0D, 2D)	PPM ‐ OP1	6.00 × 10^−3^	C+E	**ACS** [**PW**: Not reported, **PA**: 100 mV, **f**: 100 Hz, **Duration**: Not reported]	Stem cells (NSCs)	Immunofluorescence staining demonstrated a significantly higher expression of NF200 in the MAu‐GelMA ES group. Additionally, this group notably reduced inflammatory cell aggregation and glial scar formation. Histological analysis revealed minimal cavity formation and enhanced myelin regeneration. Figure 5E	[57]
Gelatin	CNT (1D)	PPM/ Covalent crosslinking ‐ OP1	1.80 × 10^−4^	C+E	**DCS** [**EFS**: 2000 mV cm^−1^ **Duration**: 7 h over 7 d]	Stem cells (NSCs)	Conductive CNT/GelMA hydrogel fibers promote enhanced neuronal differentiation and axon‐like sprouting. CNTs integrate with regenerative tissue without causing toxicity, and CNT/GelMA with electrical stimulation reduces inflammation.	[58]
HA/ Gelatin	PEDOT (0D)	Extrusion bioprinting ‐ OP1	6.00 × 10^−3^	C	—	Stem cells (NSCs)	In a rat model of complete spinal cord transection, the conductive scaffolds resulted in recovery of hindlimb motor function. Immunofluorescence staining revealed that the scaffolds effectively promoted neuronal regeneration, minimized glial scar formation, and supported myelin regeneration at the injury site.	[59]
Agarose/ Gelatin	PPy (0D)	Physical crosslinking ‐ OP1	2.00 × 10^−3^	C+N	—	Stem cells (NSCs)	The conductive supramolecular hydrogel reduced injured cavity areas and glial fibrosis formation. Its injectable nature allowed it to fill irregular cystic cavities resulting from spinal cord injuries.	[60]
HA	Germanium phosphide (2D)	Covalent crosslinking ‐ OP1	3.65 × 10^−3^	C+N	—	Stem cells (NSCs)	The GeP nanosheet hydrogel exhibited anti‐inflammatory properties, with reduced TNF‐α and increased IL‐10 levels, promoting healing in SCI. Immunostaining for neuron‐specific markers (Tuj‐1, NF200, MAP‐2) showed enhanced expression. Electrophysiological assessments confirmed that the conductive hydrogels supported propagation of motor‐evoked potentials.	[61]
PEG	GO (2D)	Physical crosslinking ‐ OP1	10^−1^	C	—	In vivo	The injectable hydrogel demonstrated anti‐inflammatory properties, inhibiting astrocyte hyperactivation and inflammation. Histological analysis revealed improved spinal cord regeneration, with enhanced immunofluorescence staining for neuronal markers (MAP‐2, and TUJ‐1) in treated spinal cord sections.	[62]
Gelatin	CNT (1D)	PPM ‐ OP1	5.00 × 10^−3^	C+N	—	Stem cells (NSCs)	The hydrogel effectively suppressed pro‐inflammatory responses and fibrosis in the spinal cord. Wogonin‐loaded carbon nanotubes enhance neurogenic differentiation and vascularization. hydrogel implantation accelerated the recovery of motor and bladder functions in SCI models.	[63]
HA	Germanium phosphide (2D)	Covalent crosslinking ‐ OP1	6.00 × 10^−3^	C+E	**MWS** [**FS**: 3 mT **f**: 2 Hz **Duration**: 1 h over 3 d]	Stem cells (NSCs)	Significant recovery in motor function and bladder control was observed, with 80% of rats in the HAG+MF group regaining spontaneous urination. The hydrogel exhibits antioxidant properties, supports nerve repair, and enhances cell proliferation and migration through magnetic‐field‐driven stimulation.	[64]
Silk	BaTiO3/ Ppy (0D)	Covalent crosslinking ‐ OP4	7.00 × 10^−2^	C+E	**UWS** [**f** : 1 MHz, **I**: 1 W cm ^−2^, **Duration**: 15 min per day – total days not reported]	In vivo	The SFBP/PER hydrogel effectively suppresses glutamate cytotoxicity, alleviates oxidative stress, and reduces inflammation in the SCI microenvironment. It enables wireless intraspinal electrical stimulation to enhance motor function recovery, reduce lesion cavity formation, promote remyelination, and support functional neuron regeneration.	[65]
Chitosan	Black phosphorus (2D)	PPM ‐ OP1	1.30 × 10^−3^	C+N	—	Cell line (PC12)	Conductive BP nanosheets enhanced the hydrogel's conductivity. The injectable hydrogel promotes vascular regeneration and neural differentiation, improving Basso, Beattie, and Bresnahan (BBB) scores in the treated groups.	[66]
Xanthan gum	rGO (2D)	Ionic crosslinking ‐ OP1	2.07 × 10^−2^	C	—	In vivo	Behavioral assessments showed improved locomotor function in rats treated with the XGrGO gel scaffold. Histological analysis revealed that the scaffold promoted the orderly growth of regenerated nerve fibers.	[67]
HA/ Gelatin	PEDOT (0D)	Covalent crosslinking ‐ OP1	8.30 × 10^−4^	C	—	Stem cells (MSCs)	In vivo assessments in a rat model of spinal cord injury showed that the scaffolds upregulated glial fibrillary acidic protein (GFAP) and reduced astrocyte reactivity. Additionally, the scaffolds promoted axon migration towards the implantation site.	[68]
Gelatin	MXene (2D)	PPM ‐ OP1	3.70 × 10^−6^	C+N	—	Stem cells (NSCs)	The unique microgroove structure of the hydrogel conduits facilitated directional growth of nerve cells. The conductive hydrogel supported connection between newly formed nerves and damaged axons and exhibited remarkable improvement in BBB score.	[69]
GelMA/ PEGDA	PEDOT (unclear)	Extrusion bioprinting ‐ OP1	1.61 × 10^−5^	C+N	—	Stem cells (NSCs)	Behavioral assessments showed that rats treated with the NSC‐laden CCH scaffold had significantly improved hindlimb locomotion, with higher BBB scores and better inclined plate performance. Histological analysis further revealed reduced glial scarring and enhanced nerve fiber regeneration.	[70]
Chitosan/ Gelatin	Black phosphorus (2D)	Covalent crosslinking ‐ OP1	2.89 × 10^−3^	C+E	**Capacitive coupling WS** [**PW**: 10 ms, **PA**: 1.1 V, **f**: 5 MHz, **Duration**: 1 h over 3 d]	Stem cells (NSCs)	The hydrogel scaffold enabled in situ wireless electrical stimulation, enhancing NSC proliferation, differentiation, remyelination, and axon regeneration, resulting in notable locomotor improvement in behavioral assessments.	[71]
GelMA/ Oxidized Dextran (ODEx)	MoS_2_ (2D)	Covalent crosslinking ‐ OP2	5.18 × 10^−4^	C+N	—	Stem cells (NSCs)	In vitro studies showed that the hydrogel promoted M2 macrophage polarization, enhancing anti‐inflammatory responses and the release of regenerative factors. In vivo, hydrogel transplantation reduced inflammation, recruited NSCs, and supported neuronal differentiation, leading to improved sensory and motor recovery.	[72]
Gelatin/ Tannic acid	Ppy (0D)	Covalent crosslinking ‐ OP4	1.29 × 10^−3^	C+N	—	In vivo	The conductive hydrogel enabled on‐demand bFGF release in response to elevated MMP‐2/9 levels in the SCI microenvironment. In vivo, MMP‐responsive bFGF reduced MMP expression, enhanced axonal regeneration, and increased blood vessel density by leveraging both conductivity and bFGF‐mediated angiogenesis.	[73]

#### 3.2.2 Brain Injury and Disease

**Figure 6:**
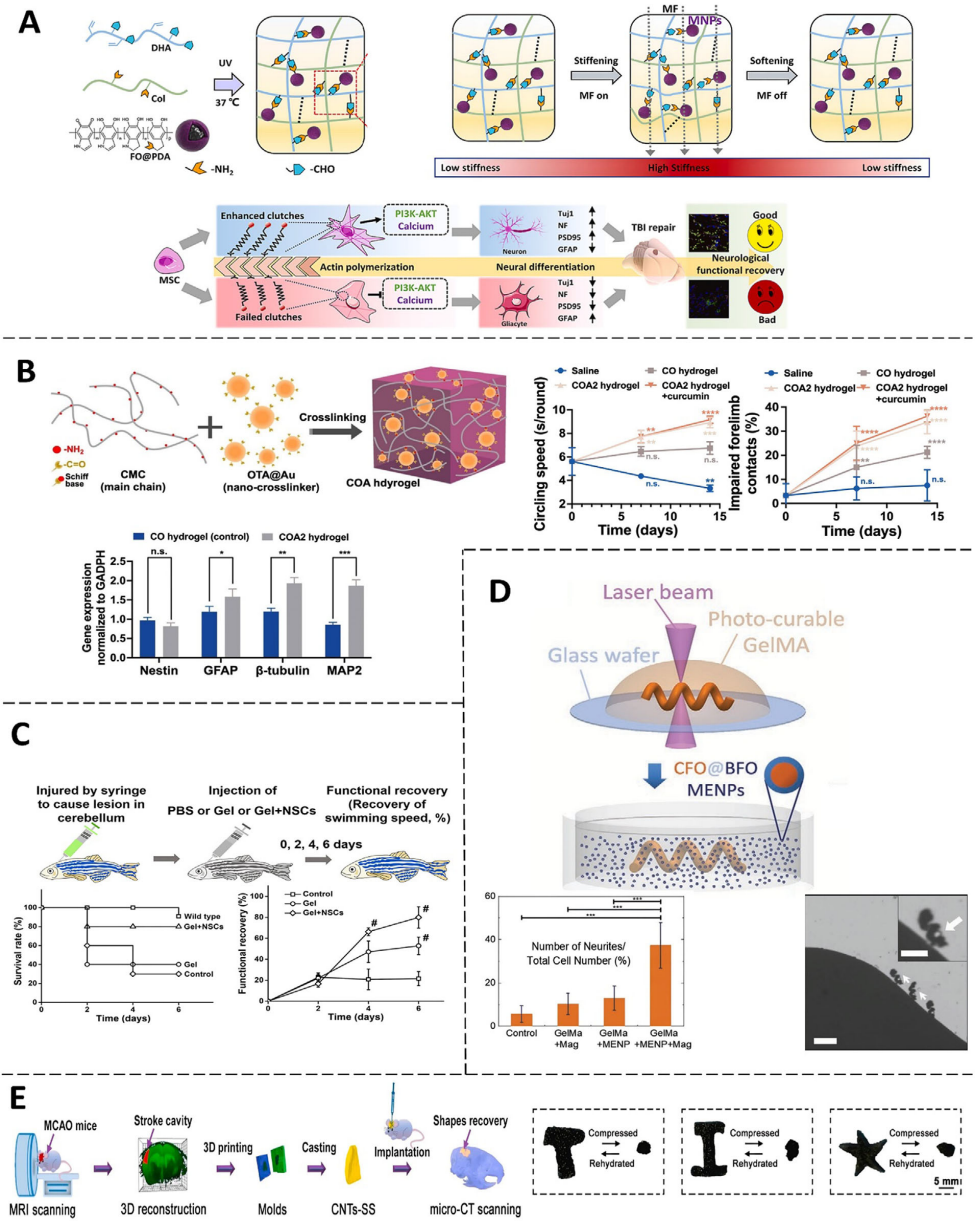
Application of Nanocomposite Conductive Hydrogels for Brain‐related Conditions. A) DHAC‐F hydrogels, with varying cross‐linking bonds, enable reversible matrix regulation of neural differentiation in BMSCs through magnetic on‐off mechanisms. This process modulates mechanotransduction via the PI3K‐AKT and calcium signaling pathways. (Adapted with permission.^[^ ^76^ ^]^ Copyright 2025, ELSEVIER) B) The gelation mechanism of COA hydrogel and its effect on neural‐related gene expression after 14 days. Functional recovery in PD rats treated with or without COA hydrogel containing curcumin was assessed by comparing left‐side circling speeds. (Adapted under the terms of the CC BY 4.0 license.^[^ ^74^ ^]^)  C)  Functional recovery of adult zebrafish after CNS injury assessed with PBS, CDD hydrogel (“Gel”), and NSC‐laden CDD hydrogel (“Gel + NSCs”). Recovery was measured by survival rate and swimming speed over 6 days.(Adapted with permission.^[^ ^77^ ^]^ Copyright 2025, American Chemical Society). D) Two‐photon polymerization (TPP) of GelMA helical microstructures, followed by incubation in core‐shell magnetoelectric nanoparticles (MENPs). The image on the right shows time‐lapse of a cell‐laden helical microswimmer actuated by a rotating magnetic field, and the one on the left displays neurite count relative to the total cell number after 10 d.  (Adapted with permission.^[^ ^81^ ^]^ Copyright 2025, JOHN WILEY AND SONS). E) CNTs‐SS applied to fill stroke cavities for brain tissue repair, compressing into three shapes that recovered to their original forms upon rehydration. (Adapted under the terms of the CC BY 4.0 license.^[^ ^80^ ^]^).

Among the studies in this area, various formulations for a range of brain injuries or disorders have been developed (**Table** **3**). Two studies aimed to incorporate anti‐inflammatory features into hydrogels to reduce neuroinflammation in Parkinson's disease (PD), while also improving conductivity via AuNPs. Xu et al. created a bioactive injectable hydrogel with Ocarboxymethyl chitosan (CMC) and oxidized tannic acid (OTA)‐modified AuNPs as a nano‐crosslinker, termed COA. This OTA@Au nano‐crosslinker provided conductivity, allowing for rapid self‐healing and excellent injectability with fine needles (34G). It demonstrated antioxidative and anti‐inflammatory effects, promoting the recovery of inflamed NSCs and increasing IL‐10 levels while decreasing pro‐inflammatory cytokines. In vivo studies in a rat PD model indicated that the stereotactic implantation of the COA2 hydrogel successfully restored normal electrophysiological functions^[^ ^74^ ^]^ (**Figure** **6**B). In another study led by the same author, a comparable formulation produced similar results, except that the negatively charged dialdehyde polyurethane nanoparticulate (DAPU) in the hydrogel played a key role in promoting the anti‐inflammatory effect.^[^ ^75^ ^]^

CNH‐based stroke therapies aim to provide neuroprotective niches for transplanted cells and enable structural repair of ischemic cavities. In one study, Zhang et al. developed a GelMA/PEDOT:PSS hybrid hydrogel serving as a niche for NSCs to facilitate recovery in a rat cerebral ischemia‐reperfusion model. The hydrogel demonstrated mechanical properties analogous to brain tissue and critical electrical conductivity for neural communication. Encapsulated NSCs in the GelMA/PEDOT:PSS100 hydrogel exhibited neuroprotection against oxygen‐glucose deprivation, notably decreasing neuronal apoptosis. In vivo studies in middle cerebral artery occlusion rats indicated excellent histocompatibility and markedly diminished inflammatory responses at the injury site.^[^ ^79^ ^]^ Additionally, Wang et al. designed an injectable silk sericin scaffold infused with CNTs for targeted repair of ischemic stroke cavities^[^ ^80^ ^]^ (Figure 6E). The scaffold's compressive moduli were adjustable through CNT concentration to align with brain tissue stiffness and exhibited notable shape‐memory characteristics, enabling quick recovery of complex shapes after compression, as described by a mathematical model linking CNT concentration to recovery kinetics. In vivo studies with CNTs‐SS allowed for real‐time monitoring via near‐infrared fluorescence and micro‐CT, ensuring accurate filling of MRI‐identified stroke cavities in MCAO stroke mice models. The scaffold demonstrated high biocompatibility and a regulated degradation profile over 56 days, offering sustained structural integrity. As a carrier for stem cells, CNTs‐SS facilitated migration of GFP‐expressing BMSCs into adjacent brain tissue and markedly improved neuronal differentiation of BMSCs, as indicated by increased Tuj1 expression, clustering of neuron‐related genes, and activation of a Tuj1 promoter‐driven mCherry reporter system.

Innovative CNHs for traumatic brain injury (TBI) repair employ divergent bioengineering strategies that collectively address structural and functional deficits in TBI. Yang et al. demonstrated that an injectable conductive hydrogel derived from Bombyx mori silk fibroin and MXene, combined with electrical stimulation at 300 mV cm^−1^, enhanced neural stem cell‐mediated repair in a TBI model. The hydrogel's porous architecture supported nutrient diffusion and cellular infiltration, exhibited mechanical properties akin to the extracellular matrix, possessed shear‐thinning injectability, and displayed adjustable electrical conductivity based on MXene concentration. In vivo experiments in TBI rat models indicated a reduction in brain cavity volume and lesion area when compared to control groups.^[^ ^78^ ^]^ In another study, Wei et al. demonstrated that Fe_3_O_4_@PDA nanoparticles facilitate remote manipulation of matrix stiffness through magnetic fields (MFs), enhancing neural differentiation of BMSCs and recovery in TBI ^[^ ^76^ ^]^ (Figure 6A). The hydrogel exhibited MFs‐dependent reversible stiffness due to magnetic nanoparticle (MNP)‐induced deformation and reinforcement. In vitro, MFs‐induced stiffening promoted BMSC elongation and spreading via a “clutch‐reinforcement” mechanism, enhancing cell‐matrix interactions through the activation of actin self‐assembly. This mechanical stimulation activated TRPV4 channels, facilitating Ca^2^⁺ influx and upregulating neuronal markers (Tuj1, NF, PSD95) while downregulating GFAP. In vivo, implantation of the ON–OFF hydrogel in TBI rats reduced brain cavity volume, promoted axonal regeneration, enhanced synaptic formation (GAP43, PSD95), and accelerated neurological recovery, as evidenced by improved spatial learning/memory (Morris water maze) compared to static hydrogel or controls, with no adverse effects. The magnetic manipulation of matrix mechanics created a dynamic microenvironment conducive to mechanotransduction‐driven neural repair.

**Table 3:** Summary of studies utilizing CNHs for brain‐related applications, detailing the materials used, fabrication techniques, conductivity, differentiation/ES settings, and type of cells. (Note: Abbreviations are explained in Sections S1.1. in the Supporting Information).

**Hydrogel Backbone**	**Nanomaterial (Dimension)**	**Fabrication & Integration Method**	**Highest Conductivity (S cm^−1^)**	**Differentiation Strategy**	**ES Setting**	**Type of Cell **	**Key Outcomes**	**Ref**.
Chitosan	AuNPs (0D)	Physical crosslinking ‐ OP1	1.36 × 10^−3^	C	—	Stem cells (NSCs)	The COA2 hydrogel reduced GFAP‐positive astrocytes, indicating lower astrocyte response and inflammation in PD models. It also preserved dopaminergic neurons and fibers in the substantia nigra and striatum, showing promise for neurodegenerative disease treatment.	[74]
Chitosan/ polyurethane	AuNPs (0D)	Covalent crosslinking ‐ OP1	4.00 × 10^−3^	C	—	Stem cells (NSCs)	The hydrogel exhibited anti‐inflammatory effects and decreased neuroinflammation. In a PD rat model, it improved motor recovery and reduced neurodegeneration.	[75]
HA/ Collagen	Iron Oxide NPs (0D)	PPM ‐ OP1	—	C+N+E	**Pulsed MWS** [**FS**: 100 mT **f**: Not reported **Duration**: 3.5 h over 7 d]	Stem cells (MSCs)	Leveraging the safety of MF loading, magnetic manipulation effectively promoted TBI repair, enhancing neurological recovery. The on–off magnetic effect stimulated BMSC neural differentiation via activation of PI3K‐AKT and calcium signaling pathways.	[76]
Chitosan/ polyurethane	PPy (0D)	Covalent crosslinking ‐ OP1	6.00 × 10^−3^	C	—	Stem cells (NSCs)	The conductive scaffold improved locomotion in brain‐injured zebrafish, increasing swimming speed.	[77]
Silk	MXene (2D)	Self‐assembly ‐ OP1	2.90 × 10^−3^	C + E	**DCS** [**EFS**: 300 mV cm^−1^ **Duration**: 1.5 h over 3 d]	Stem cells (NSCs)	In TBI rat models, the conductive hydrogel with electrical stimulation improved motor recovery, reduced glial scarring, and enhanced neuronal regeneration. It increased neuronal differentiation and axon length, while also preserving brain tissue integrity.	[78]
Gelatin	PEDOT (Unclear)	PPM ‐ OP1	1.40 × 10^−4^	C	—	Stem cells (NSCs)	After implantation in a rat model of middle cerebral artery occlusion (MCAO), the hydrogel reduced inflammatory responses, indicating its potential to mitigate the adverse effects of cerebral ischemia‐reperfusion injury.	[79]
Silk	CNT (1D)	Covalent crosslinking ‐ OP1	—	C	—	Stem cells (MSCs)	This research introduces CNTs‐SS shape‐memory scaffolds for personalized brain repair after severe stroke. These scaffolds fit irregular stroke cavities, enable real‐time tracking via near‐infrared photoluminescence, and promote neuronal differentiation, advancing stroke treatment.	[80]
Gelatin	Cobalt ferrite/ Bismuth ferrite (0D)	3D bioprinting ‐ OP1	—	C+E	**MWS** [**FS**: 50 mT **f**: 1053 Hz **Duration**: 10 h over 10 d]	Cell line (SH‐SY5Y)	ENP microswimmers effectively deliver viable therapeutic cells and promote neuronal regeneration. Under magnetic stimulation, they enhance SHSY5Y cell differentiation and multipolar cell formation, showing promise for neurodegenerative disease treatment. Figure 6D	[81]
Chitosan	Graphene Nano Platelets (2D)	Solvent casting ‐ OP1	7.70 × 10^−7^	C	—	Cell line (HT‐22)	Chitosan‐GNP aligned scaffolds enhanced HT22 cell adhesion and promoted radial neurite extension, mimicking pyramidal cells—key for brain cell regeneration.	[82]

Among studies with general brain‐related applications, Gosh et al. engineered Chitosan/ graphene nanoplatelets (GNP) nanocomposite scaffolds for brain cell regeneration, utilizing carboxyl‐functionalized GNPs aligned through electric fields. This alignment significantly improved the mechanical strength and electrical conductivity necessary for neural signal transmission in compromised circuits. In vitro, these scaffolds optimized hippocampal neuron proliferation, neurite extension, and intercellular network formation due to GNP‐mediated charge transfer and ECM‐mimetic topography.^[^ ^82^ ^]^ In a study by Xu et al., a multifunctional electroconductive system was developed through the crosslinking of *N*‐carboxyethyl chitosan, chitosan‐modified polypyrrole nanoparticles, and aldehyde‐terminated difunctional polyurethane, resulting in a self‐healing hydrogel with shape‐recoverable properties. This hydrogel facilitated neural stem cell adhesion, proliferation, and differentiation, improved motor function in brain‐injured zebrafish, and allowed for real‐time strain sensing during various mechanical and biological activities^[^ ^77^ ^]^ (Figure 6C).

### 3.3 CNHs for PNS Applications

#### 3.3.1 Peripheral Nerve Injury

**Figure 7:**
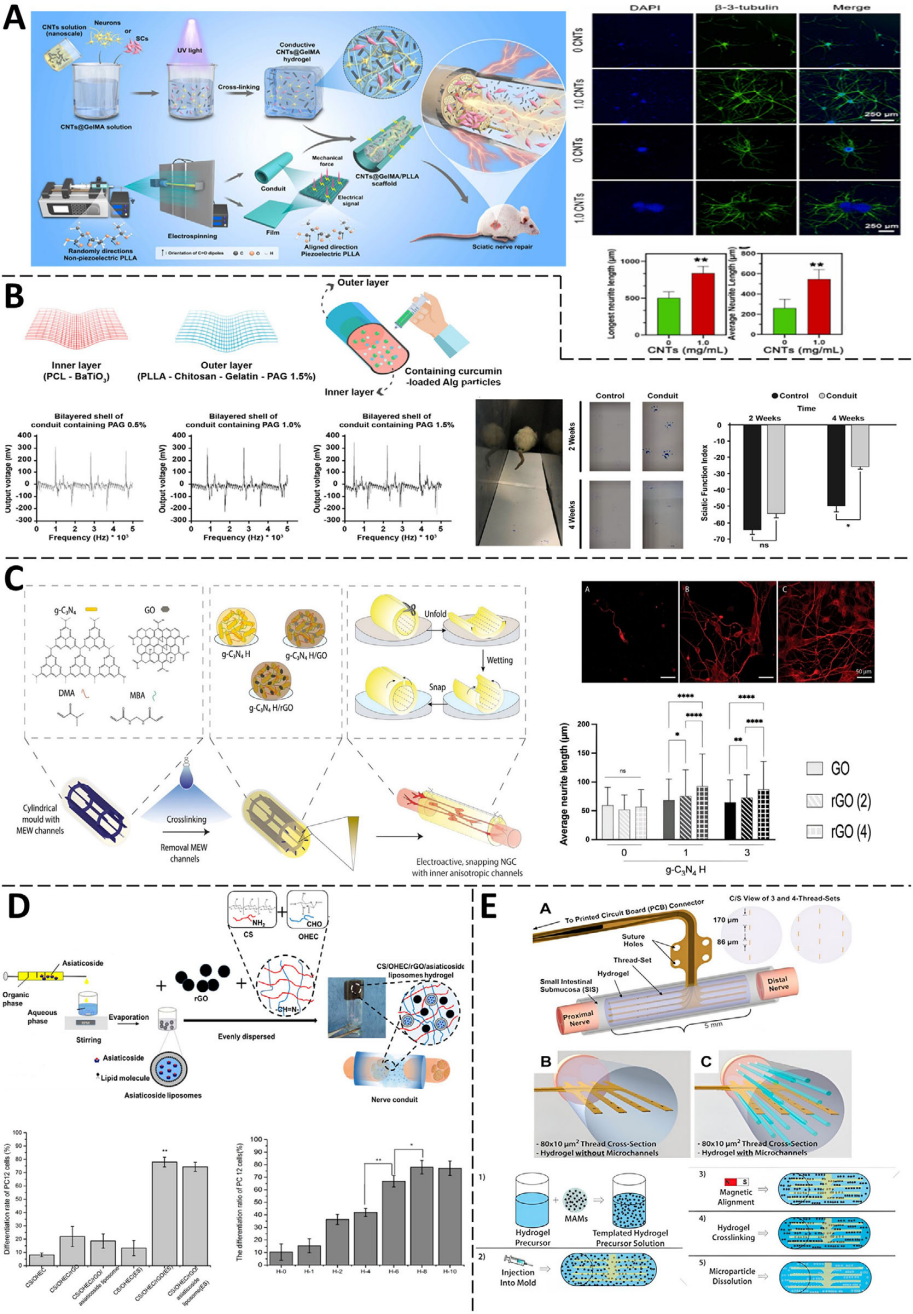
Applications of CNHs in Nerve Repair. A) Application of a wirelessly self‐powered, electroconductive CNTs@GelMA/PLLA scaffold. On the right: representative fluorescence images of neurons cultured on the scaffold for 7 d and the quantitative analysis of maximum and average neurite lengths, respectively (Adapted with permission.^[^ ^101^ ^]^ Copyright 2025, ELSEVIER). B) A bilayered fibrous conduit was made by electrospinning an inner PCL/BaTiO_3_ piezoelectric layer and coating it with an outer PLLA sheath containing PAG, chitosan, and gelatin. Curcumin‐loaded alginate microparticles in gellan filled the lumen. Piezoelectric output was measured at 0.5%, 1%, and 1.5 wt% PAG, and functional recovery in a rat sciatic transection model was tracked via SFI over 4 weeks. (Adapted with permission.^[^ ^102^ ^]^ Copyright 2025, ELSEVIER). C) An anisotropic, conductive gC_3_N_4_/DMAMBA hydrogel conduit with aligned meltelectro‐written microchannels snaps back to its cylindrical form upon hydration. TUJ1‐stained PC12 cells on g‐C_3_N_4_ H/GO_3_, g‐C_3_N_4_ H/rGO_3_ (2), and g‐C_3_N_4_/H/GO_3_ (4) scaffolds exhibited progressively enhanced neurite outgrowth. (Adapted under the terms of the CC BY‐NC license.^[^ ^105^ ^]^ Copyright 2025, ELSEVIER). D) The electrostimulation and PC12 differentiation effect of chitosan/oxidized hydroxyethyl cellulose/reduced graphene oxide/asiaticoside liposome‐based hydrogels over increasing conductivities (H‐0 to H‐10) (Adapted with permission.^[^ ^106^ ^]^ Copyright 2025, ELSEVIER) E) Schematic of the MARTEENI implant: a hydrogel sheath encases decellularized small intestine submucosa with embedded polyimide electrodes. B,C) Cross‐sections comparing polyimide threads in an untemplated hydrogel versus a magnetically templated hydrogel with aligned microchannels. Bottom: Magnetic templating process using alginate microparticles. (Adapted with permission.^[^ ^100^ ^]^ Copyright 2025, ELSEVIER).

The summary of key aspects is outlined in **Table** **4**. A noteworthy advancement in sciatic nerve regeneration has emerged through the development of wirelessly and self‐powered piezoelectric stimulation systems, which reduce the reliance on external power sources while enabling on‐demand therapeutic activation. A novel piezoelectric bilayer conduit by Delavar et al. demonstrated significant improvement in sciatic nerve regeneration in rat subjects ^[^ ^102^ ^]^ (**Figure** **7**B). The conduit's core–shell design utilized an inner layer of BaTiO_3_‐infused PCL nanofibers to produce endogenous electrical stimulation via physiological mechanical forces, thus eliminating the need for external power. This piezoelectric component was paired with an outer PLLA‐CS‐Gel‐Polyaniline/Graphene (PAG) nanocomposite layer to enhance structural integrity. Additionally, the lumen contained gellan gum with curcumin‐loaded alginate particles for prolonged anti‐inflammatory drug release. The conduit's combined benefits, including piezoelectric‐generated electrical stimulation from BaTiO_3_, structural support from aligned fibers, and regulated curcumin delivery, expedited axonal regeneration and functional recovery, indicating a promising wireless therapeutic approach for nerve repair. Complementing this approach, Xu et al. demonstrated a wireless, self‐powered piezoelectric system for peripheral nerve regeneration using ultrasound as a non‐invasive trigger. The system comprised electrospun, aligned nanofibers from barium titanate nanoparticle‐doped P(VDF‐TrFE), forming the inner layer of nerve guidance conduits. These nanofibers converted ultrasound energy into localized electrical stimulation, negating the need for implanted components. In vivo studies in rat models showed that nerve guidance conduit (NGCS) with these nanofibers and ultrasound stimulation enhanced axonal regeneration, functional recovery, and reduced muscle atrophy compared to controls. The ultrasound also facilitated the on‐demand release of nerve growth factor (NGF) from a thermoresponsive hydrogel, further improving regenerative outcomes.^[^ ^83^ ^]^ Lastly, Yang et al demonstrated that an engineered wirelessly self‐powered CNTs@GelMA/PLLA scaffold improved peripheral nerve regeneration ^[^ ^101^ ^]^ (Figure 7A). The scaffold's aligned PLLA nanofibers exhibited piezoelectric properties, generating electrical stimulation from mechanical deformation autonomously. This stimulation was enhanced by the conductive microenvironment of the CNT‐doped GelMA hydrogel, forming a synergistic electroconductive network. In vivo testing in a rat sciatic nerve defect model indicated that this scaffold facilitated functional recovery, as evidenced by improved Sciatic Function Index (SFI) scores and enhanced compound muscle action potential parameters comparable to autograft. These self and wirelessly‐powered approaches collectively demonstrated clinical translation potential by addressing major limitations of conventional electrical stimulation systems including device maintenance, battery replacement, and surgical complications, while maintaining therapeutic efficacy for nerve regeneration applications.

A suite of magnetically responsive composite hydrogels and scaffolds has further advanced sciatic nerve repair by combining targeted stimulation with biomimetic architecture and cell delivery. Wang et. al illustrated that a magnetically enhanced, dual‐oriented fibrous scaffold (OCFP) enabled wireless, on‐demand electrical stimulation for effective peripheral nerve repair. The scaffold comprised cobalt ferrite (CFO) nanofibers magnetically aligned parallel to polyvinylidene fluoride (PVDF) fibers during electrospinning, boosting magnetoelectric coupling. Under a low‐frequency AC magnetic field (1000 Hz, 150 Oe), the OCFP scaffold generated targeted electrical potentials (≈150 mV) – sufficient to modulate neural activity – without implanted electrodes or batteries. In a rat model of sciatic nerve injury, the OCFP scaffold combined with magnetic field stimulation (OCFP+M group) resulted in improved motor function by week 8 compared to controls, as evidenced by gait analysis and Tarlov scoring.^[^ ^96^ ^]^ Similarly, an anisotropic magnetic‐conductive hydrogel scaffold developed by Li et al. enhanced nerve regeneration by integrating magnetically aligned PLGA/Fe_3_O_4_ short fibers (MSFs) and multi‐MWCNTs within a sodium alginate/carboxymethyl chitosan (SA/CMCS) matrix. Under a magnetic field (300 mT), MSFs oriented within ±15° provided guidance for neuronal growth. In vitro, the combination of magnetic and electrical stimulation on neurons cultured on aligned scaffolds exhibited synergistic effects, with ES+MS enhancing cell viability compared to controls and surpassing individual modalities.^[^ ^103^ ^]^ Furthermore, a novel superparamagnetic Mag‐gel scaffold, actuated by a rotating magnetic field (RMF), enhanced peripheral nerve regeneration through extracellular vesicles (EV) generation. RMF actuation provided mechanical stimulation to Schwann cells (SC), inducing calcium influx via PIEZO2 channel activation and increasing therapeutic SC‐derived EVs (SC‐EVs) secretion. RNA‐seq analysis indicated that EVs generated under Mag+RMF stimulation exhibited a favorable molecular composition, showing enrichment of pro‐angiogenic factors and inflammation regulators.^[^ ^95^ ^]^ Moreover, Kasper et al. developed a magnetically aligned regenerative tissue‐engineered electronic nerve interface (MARTEENI) successfully integrating regenerative scaffolding with neural interfacing capabilities^[^ ^100^ ^]^ (Figure 7E). Magnetic templating process utilized magnetic alginate microparticles to guide the formation of linear channels within the hydrogel, promoting Schwann cell proliferation and axonal growth in close proximity to the electrodes, which was expected to improve the selectivity and efficacy of nerve signal recording and stimulation. Lastly, a magnetically actuated helical hydrogel micromotor by Liu et al. served as an effective platform for targeted NSC delivery to promote nerve regeneration. The micromotors, created using a capillary microfluidic chip, embedded Fe_3_O_4_ magnetic nanoparticles in an alginate hydrogel matrix, allowing for precise navigation to injury sites in Xenopus laevis sciatic nerve models through external magnetic field manipulation. This magnetic system facilitated high‐density NSC transport while reducing cell loss and preserving viability. Upon arrival at the target site, enzyme‐responsive degradation of the alginate enabled controlled release of NSCs.^[^ ^93^ ^]^

Across diverse platforms, scaffold architecture emerged as a unifying driver of regenerative efficacy by recapitulating key biophysical cues of the native nerve milieu. In a study, self‐snapping anisotropic channels were created using sacrificial melt electrowriting of PCL fibers embedded in a g‐C_3_N_4_/GO hydrogel precursor, followed by blue‐light crosslinking^[^ ^105^ ^]^ (Figure 7C). After crosslinking and dialysis, the PCL template was removed with dichloromethane, resulting in hollow microchannels that mirrored the printed design. Notably, optimization of channel sizes indicated that the smallest microchannels (≈10 µm diameter post‐hydration) offered superior topographical guidance, promoting maximal alignment and directional extension of PC12 cell neurites. Meanwhile, Han et al. leveraged digital light processing (DLP) to fabricate a sophisticated nerve guidance scaffold with a precisely engineered triphasic architecture comprising GelMA, chitosan, and PEDOT. The core structural innovation lay in the DLP‐printed microlattice framework, which integrated a honeycomb and auxetic structures for mechanical stability and fracture resistance under deformation. Furthermore, interfacial polymerization was utilized to uniformly apply PEDOT nanoparticles to the internal pore surfaces of the GelMA/CS hydrogel, creating conductive pathways while preserving its complex structure. This multifaceted design facilitated 3D topography for cellular infiltration and electroconductivity, thereby promoting thicker myelinated axons, diminishing gastrocnemius muscle atrophy, and achieving functional recovery akin to autografts.^[^ ^92^ ^]^ Pillai et al. examined the influence of macroscale textile architecture in silk fibroin‐based artificial nerve conduits on peripheral nerve regeneration. The primary focus was on comparing three core structures:knitted, braided, and twisted, constructed from degummed silk fibroin threads coated with a poly‐*ε*‐caprolactone/carbon nanofiber nanocomposite. Architectural analysis indicated that the knitted structure, especially at 10% carbon nanofiber loading, exhibited enhanced properties, including the formation of interconnected macrochannels, optimal mechanical compliance, regulated porosity, and advantageous swelling characteristics. In vivo studies using a rabbit sciatic nerve injury model validated the knitted conduit's effectiveness in promoting rapid recovery of near‐normal limb function within a 30‐day period.^[^ ^98^ ^]^ Finally, Hu et al. developed a bioinspired NGC mimicking Morpho butterfly wing architecture, characterized by parallel nanoridges. The wings underwent oxygen plasma treatment for hydrophilicity, followed by the incorporation of GO nanosheets into the inter‐ridge spaces. Hydrohalic acid reduction transformed GO into conductive reduced rGO, improving electrical conductivity. This rGO‐embedded wing was subsequently coated with methacrylated GelMA hydrogel containing brain‐derived neurotrophic factor (BDNF), culminating in a functional conductive NGC scaffold. The maintained anisotropic nanoridge topography offered critical contact guidance, promoting alignment and neurite outgrowth of PC12 and NSC cells along the ridges. Additionally, the rGO integration enhanced electrical signaling and neural network functionality, while the GelMA hydrogel facilitated sustained BDNF release, collectively fostering axon regrowth and functional regeneration of nerve and muscle tissues.^[^ ^85^ ^]^

Multifunctional conductive hydrogels served as advanced drug delivery platforms providing spatiotemporal delivery of bioactive agents to orchestrate angiogenesis, immunomodulation, and neurotrophic support for enhanced nerve repair. In a study by Xu et al., a GelMA/ECM–SiP@PDA conductive hydrogel was housed within a double layer nerve guide conduit to create a multifunctional regeneration platform. Polydopamine‐coated silicon phosphide nanosheets (SiP@PDA) provided a biodegradable source of silicon ions, released in a controlled manner to sustain angiogenesis via HIF1α, VEGF, and bFGF upregulation. Concurrently, the hydrogel niche directed transplanted MSCs toward Schwann cell fates, boosting endogenous neurotrophin release for axonal regrowth and myelination. Together, ionic release and conductivity skewed macrophages to an M2 phenotype, resolving inflammation and, in sciatic defect models, markedly improving functional nerve recovery.^[^ ^91^ ^]^ Moreover, a chitosan/oxidized hydroxyethyl cellulose (CS/OHEC) hydrogel was developed by Zheng et al. for peripheral nerve regeneration^[^ ^106^ ^]^ (Figure 7D). Asiaticoside was encapsulated in liposomes with 70.2% efficiency for controlled release from the hydrogel's 3D network. This sustained release inhibited fibroblast proliferation and collagen formation, thereby reducing scar formation essential for regeneration. Additionally, the hydrogel contained 8% rGO to enhance electrical conductivity, which, when combined with electrical stimulation (ES), improved PC12 differentiation through biochemical synergy. Consequently, the scaffold's dual‐function release system fostered an anti‐fibrotic and pro‐regenerative microenvironment within nerve conduits. In a separate investigation, a conductive hydrogel based on silk fibroin was created through the covalent cross‐linking of thiolated SF with maleimide‐functionalized graphene oxide (Mal‐GO) and the integration of fibroblast‐derived exosomes (EXOs). The released EXOs enhanced angiogenesis via the activation of NOTCH signaling, increased neurotrophic factors (NGF, BDNF), and facilitated axonal regrowth and remyelination. Concurrently, exosomes mitigated the cytotoxicity of GO while enhancing neuronal responsiveness to conductive stimuli.^[^ ^90^ ^]^ Lastly, Wang et al. engineered a conductive hydrogel (GACM) incorporating GelMA, adenine,  thymine‐functionalized carbon nanotubes (CNT‐Thy), and mesaconate for cavernous nerve repair. The CNT‐Thy framework promoted mesaconate release, influencing macrophage polarization towards anti‐inflammatory M2 phenotypes by inhibiting IL‐6 and inflammatory signals, while also boosting Schwann cell activity and neurotrophic factor release. This bioactive system mitigated fibrosis in cavernous tissue by enhancing muscle/collagen ratios and reducing apoptosis, while leveraging CNT‐Thy's conductivity to promote axonal regeneration. The hydrogel's sprayable adhesive property enabled minimally invasive application, restoring erectile function and achieving fertility recovery in animal models through targeted immunomodulation combined with electroactive scaffolding.^[^ ^94^ ^]^

**Table 4:** Summary of studies utilizing CNHs for nerve injury applications, detailing the materials used, fabrication techniques, conductivity, differentiation/ES settings, and type of cells. (Note: Abbreviations are explained in Sections S1.1. in the Supporting Information).

**Hydrogel Backbone**	**Nanomaterial (Dimension)**	**Fabrication & Integration Method**	**Highest Conductivity [S cm^−1^]**	**Differentiation Strategy**	**ES Setting**	**Type of Cell**	**Key Outcomes**	**Ref**.
NIPAM/ AAm/GelMA	Barium Titanate (0D)	PPM‐ other	—	C+E	**UWS** [**f** : 1 MHz, **I**: 0.75 W cm^−2^, **Duration**: 0.7 h over 7 d]	Cell line (PC12)	Ultrasound‐responsive piezoelectric nanofiber‐based hydrogel promoted neurite outgrowth and sciatic nerve repair through ultrasound‐triggered electrical stimulation. Also, the thermoresponsive hydrogel enabled controlled NGF release by shrinking under ultrasound‐induced heating.	[83]
Alginate	Graphene (2D)	Ionic crosslinking‐ OP1	—	C	—	In vivo analysis	The GR‐SA scaffold enhanced sciatic nerve growth factors while reducing inflammation. SFI values confirmed improved recovery with GR‐SA hydrogel treatment.	[84]
Gelatin	Graphene (2D)	PPM ‐ OP1	—	C+N	—	Stem cells (NSC)	In vitro, PC12 cells and NSCs cultured on rGO‐BDNF‐GelMA wings showed enhanced neurite outgrowth and alignment along nanoridges. In vivo, NGCs promoted axon regrowth and functional nerve‐muscle regeneration in rats with 10 mm sciatic nerve defects. The sustained neurotrophic factor release also supported NSC growth and maturation.	[85]
Polyacrylonitrile	Carbon quantum dots (0D)	3D bioprinting ‐ OP1	—	C+N	—	Stem cells (MSC)	In a rat model with a 3 mm sciatic nerve defect, The PAN conduit filled with fibrin hydrogel and GQDs enhanced nerve regeneration. Histological analysis confirmed increased axon density and remyelination in the Schwann cell‐laden conduit, demonstrating effective nerve repair.	[86]
Silk/Collagen	AuNPs (0D)	Self assembly ‐ OP1	7.24 × 10^−8^	C+E	**ACS** [**PW**: 0.1 ms, **PA**: 1.2 V, **f**: 20 Hz, **Duration**: 0.5 h over a day]	Stem cells (MSC)	Ultrastructural analysis showed that conductive conduits with ES reduced Wallerian degeneration, enhancing myelin sheath thickness and axonal integrity. ES also increased BDNF and neurofilament expression, further supporting nerve regeneration.	[87]
Gelatin	rGO (2D)	Thermal Gelation ‐ OP1	10^−3^	C	—	Cell line (PC12)	rGO/GelMA conduits enhanced peripheral nerve regeneration, improving muscle weight and electro‐conduction velocity. They supported PC12 cell growth and differentiation, with functional recovery comparable to autografts after 8 weeks.	[88]
Alginate	Graphene (2D)	Covalent crosslinking‐ Other	—	C	—	Stem cells (ADSC)	ADSCs in graphene foam hydrogel scaffolds reduced muscle atrophy in a diabetic mouse model, with the highest gastrocnemius muscle wet weight in the ADSCs‐loaded group. ADSCs were found to reduce oxidative stress and inflammation while promoting cell proliferation, angiogenesis, and Schwann cell differentiation.	[89]
PEG/Silk	GO (2D)	PPM ‐ OP1	6 × 10^−3^	C	—	Stem cells	SGP‐EXOs Hydrogels showed a higher muscle fiber cross‐sectional area and significant axon regeneration, with improved myelination of regenerated axons. Additionally, graphene oxide enhanced electron transmission essential for cellular activities, while fibroblast exosomes provided bioactive signals for nerve repair.	[90]
Spinal cord ECM/ Gelatin	SiP (2D)	PPM ‐ OP1	10^−2^	C+N	—	Stem cells (MSC)	The GelMA/ECM‐SiP@PDA hydrogel regulates the nerve injury microenvironment, improving inflammation by promoting M2 macrophage polarization. It also supports MSC differentiation into Schwann cells, aiding myelination and neurite regrowth. Additionally, the degradation of SiP nanosheets releases bioactive ions that stimulate angiogenesis.	[91]
Gelatin/ Chitosan	PEDOT (0D)	DLP bioprinting ‐ OP1	9.50 × 10^−2^	C+E	**DCS** **[EFS**: 100 mV cm^−1,^ **Duration**: 2.5 h over 5 d]	Cell line (PC12)	DLP 3D printing enabled the fabrication of GelMA/CS‐PEDOT hydrogels into intricate, conductive 3D structures. In vitro, PEDOT‐treated hydrogels with DC stimulation enhanced PC12 proliferation, maturation, and axonal growth. In vivo, conductive hydrogel nerve conduits promoted nerve regeneration and muscle recovery in 10‐mm sciatic nerve defects in rats.	[92]
Alginate	Iron Oxide NPs (0D)	Ionic crosslinking‐ OP1	—	C+E	**MWS** [**FS**: 3.6 mT **f**: 3 Hz **Duration**: Not reported]	Stem cells (NSC)	At targeted sites, alginate hydrogel micromotors developed with capillary microfluidic chips enabled enzyme‐responsive cell release and NSC proliferation along the spiral framework. Released NSCs formed neuronal connections, restoring neural circuits while maintaining differentiation capacity and supporting functional neural repair in vivo.	[93]
Gelatin	CNT (1D)	PPM ‐ OP1	3 × 10^−2^	C	—	Stem cells (NSC)	A sprayable adhesive conductive hydrogel is designed for cavernous nerve repair after prostate surgery. Composed of gelatin, adenine, carbon nanotubes, and mesaconate, it provides strong adhesion, anti‐swelling properties, and inflammation suppression. By forming an electrical bridge, it promotes axon extension and enhances Schwann cell activity. In vivo tests confirmed its effectiveness in restoring erectile function and improving fertility.	[94]
PAAm/HA	PEI (0D)	Covalent crosslinking‐ OP1	—	C+E	**MWS** [**FS**: 100 mT, **f**: 1 Hz **Duration**: 14 h over 7 d]	Schwann cells	The superparamagnetic hydrogel enhanced nerve repair by regulating Schwann cell‐derived extracellular vesicle (EV) secretion through magneto‐mechanical stimulation. A rotating magnetic field increased EV release, promoting axon growth and tissue repair. Transcriptome analysis confirmed a pro‐regenerative EV profile linked to axon growth, angiogenesis, and inflammation control.	[95]
Gelatin	Cobalt ferrite (0D)	Not reported ‐ Other	—	C + E	**MWS** [parameters not reported]	Stem cells (NSC)	The hydrogel generated ≈150 mV under a low‐frequency magnetic field and increased nerve growth factor expression for repair. In vivo, it significantly improved motor function recovery in rats with sciatic nerve injuries, as evidenced by gait analysis and histological evaluations.	[96]
Collagen	Iron oxide NPs (0D)	Covalent crosslinking‐ OP1	—	C+N	—	Cell line (PC12)	Magnetic nanoparticles (MNPs) enabled controlled collagen fiber alignment, guiding neuronal growth. In vivo, conduits filled with magnetically aligned gels enhanced axonal regeneration in a rat sciatic nerve injury model, showing a higher axon count than controls. Genipin binding prolonged NGF bioavailability.	[97]
PCL/Silk	Carbon nanofiber (1D)	Covalent crosslinking/ self‐ assembly ‐ OP1	—	C	—	Progenitor cells (NPCs) / Cell line (Neuro2a)	In vivo, silk fibroin conduits enhanced axonal regeneration and functional recovery in a rat sciatic nerve injury model. Histological analysis showed improved Schwann cell migration and proliferation, supporting nerve repair.	[98]
Chitin	PEDOT (0D)	Physical crosslinking‐ OP1	2.8 × 10^−4^	C + E	**DCS** **[EFS**: 100 mV cm^−1,^ **Duration**: 2.3 h over 7 d]	Cell line (RSC‐96)	In vivo evaluation of rat sciatic nerve repair showed that PEDOT NPs in chitin hydrogel enhanced nerve regeneration, with results comparable to autografts in terms of muscle weight, fiber area, CMAP, and myelin thickness. Immunofluorescence analysis indicated improved Schwann cell adhesion and angiogenesis at the injury site.	[99]
HA	Iron oxide NPs (0D)	PPM ‐ OP4	—	C	—	Schwann cells	The MARTEENI device combines a magnetically templated hydrogel with microelectrode threads to guide nerve growth and promote regeneration. It promoted cellular infiltration and extracellular matrix deposition, supporting nerve regeneration in a rat sciatic nerve injury model. High axon densities were observed through the implant.	[100]
GelMA/ PLLA	CNT (1D)	PPM ‐ OP1	5.8 × 10^−3^	C+N	—	Schwann cells	CNTsGelMAPLLA groups exhibited enhanced sciatic functional index scores and reduced muscle atrophy. Immunohistochemical analysis revealed a higher density of NF160‐positive nerve fibers and MBP‐positive myelinated Schwann cells, indicating effective regeneration. Additionally, the scaffold promoted thicker myelin sheaths and larger axon diameters compared to controls.	[101]
Alginate/ Gellan Gum	BaTiO3 (0D)	Ionic crosslinking ‐ Other	—	C+E	Self‐generated electrical stimulation	In vivo	The fabricated conduit facilitated localized electrical stimulation due to BaTiO_3_ in its fibrous layer, while encapsulated curcumin reduced oxidative stress and inflammation. Functional assessments, including walking track analysis, demonstrated significant motor function improvement, with enhanced sciatic functional index scores over time in the conduit‐treated group.	[102]
Alginate/ Chitosan/ PLGA	MWCNTs / Iron Oxide NPs (0D, 1D)	Ionic crosslinking‐ OP1	4.15 ×10^−5^	C +E	**MWS** [**FS**: 300 mT **Duration**:120 h over 5 d] **PES** [**EFS**: 250 mV, **f**: 20 Hz, **Duration**:30 h over 5 d	Primary cortical neurons	The composite hydrogel with an aligned structure increased cell viability in cultured cortical neurons compared to individual magnetic or conductive hydrogels. Combined electrical and magnetic stimulation further enhanced neuronal growth, and in a sciatic nerve injury model, it reduced mice's licking latency.	[103]
Gelatin, PEG, Gellan Gum	Carbon nanofiber (1D)	Extrusion bioprinting ‐ OP1	7.5 ×10^−4^	C+N	—	Cell line (Neuro2a)	Nerve conduits (2–8 mm inner diameter, 20 mm length) were successfully 3D printed at 21 °C. N2a cells maintained over 80% viability, while CNF addition increased electrical conductivity fourfold. In differentiation media, all scaffolds promoted neurite outgrowth, indicating neural differentiation.	[104]
g‐c3N4	GO (2D)	PPM ‐ OP1	—	C+N	—	Cell line (PC12)	All hydrogels exhibited optimal mechanical stiffness for peripheral nerve regeneration, were biocompatible, and supported PC12 cell proliferation and differentiation. Electroactive rGO (1 and 3 mg mL^−1^) hydrogels enhanced neural differentiation. PC12 cells on g‐C3N4 HrGO3 hydrogels showed a 47% increase in neurite length. Aligned microchannels in NGCs effectively directed neurite outgrowth.	[105]
Chitosan/ Cellulose	rGO (2D)	Covalent Crosslinking ‐ OP1	5.27×10^−4^	C+E	**DCS** **[EFS**: 250 mV, **Duration**: 8 h over 1 d]	Cell line (PC12)	The porous 3D structure supported asiaticoside release and nerve cell growth with high biocompatibility. PC12 cells in conductive rGO with electrical stimulation showed enhanced differentiation. The CS/OHEC/rGO/asiaticoside liposome hydrogel, combining a natural drug and electrical stimulation, can address nerve conduit limitations.	[106]

### 3.4 Other PNS Applications

**Figure 8:**
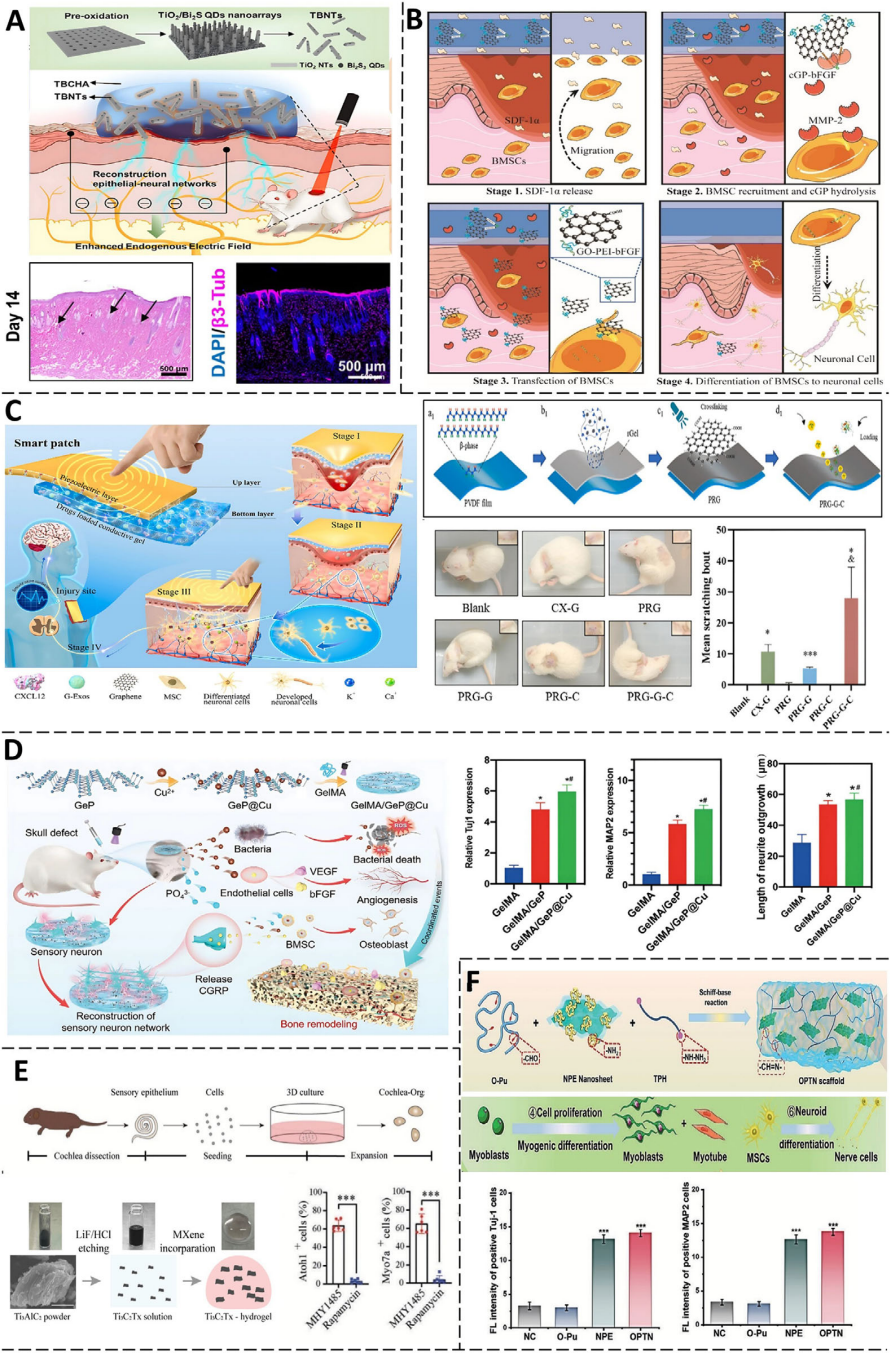
Application of CNHs for PNS. A) Schematic of red‐light‐driven photoelectric hydrogel dressings. Bottom left: H&E staining shows skin appendage regeneration (black arrows). Bottom right: Fluorescence immunohistochemistry reveals nerve ending maturation (purple) on day 14.  (Adapted with permission.^[^ ^108^ ^]^ Copyright 2025, ELSEVIER). B) GO‐based gene delivery system promotes nerve regeneration by releasing SDF‐1α to recruit BMSC, which overexpress MMP‐2, hydrolyzing cGP‐bFGF and releasing GO‐PEI‐bFGF to deliver pDNAs‐bFGF, leading to neuronal differentiation. (Adapted with permission.^[^ ^110^ ^]^ Copyright 2025, Royal Society of Chemistry).  C) PRG‐G‐C promotes sensory recovery in skin. Top right: PRG‐G‐C composition and fabrication. Bottom right: Scratch behavior and average scratching bouts over 30 min across groups. (Adapted with permission.^[^ ^109^ ^]^ Copyright 2025, ELSEVIER).  D)  GelMA/GeP@Cu electroactive hydrogel promotes nerve fiber ingrowth and neural differentiation, with in vitro results showing PC12 cell differentiation. (Adapted with permission.^[^ ^115^ ^]^ Copyright 2025, JOHN WILEY AND SONS LICENSE). E)  Primary cochlea cell isolation, culture, and Cochlea‐Org formation. Bottom left: Preparation of Ti_3_C_2_T_x_ MXene and MXene‐Matrigel hydrogel. Bottom right: Atoh1‐GFP+ hair cells in organoids after 5‐d treatment with MHY1485 and rapamycin. (Adapted under the terms of the CC BY 4.0 license.^[^ ^118^ ^]^) F) Multifunctional OPTN scaffolds support VML regeneration and neurogenesis. Bottom: Quantitative analysis of MSCs' neurite differentiation.  (Adapted with permission.^[^ ^117^ ^]^ Copyright 2025, JOHN WILEY AND SONS).

Key aspects are presented in **Table** **5**. Recent studies have leveraged multifunctional nanocomposite hydrogels, integrating conductive nanomaterials and stimuli‐responsive mechanisms such as photothermal, photoelectrical, and piezoelectric effects, to synergistically promote neurovascular niche reconstruction and sensory recovery in diabetic wound healing models. Jiang et al. synthesized a photothermally responsive nanocomposite hydrogel with conductive polydopamine‐reduced graphene oxide (pGO), achieving notable success in restoring neural networks and mechanical nociception in infectious diabetic ulcers (IDUs). This hydrogel utilized pGO's conductivity and photothermal properties for NIR‐mediated controlled antibiotic delivery. Additionally, pGO attracted Trem2⁺ macrophages, facilitating collagen remodeling and enhancing angiogenesis through the VEGF‐eNOS pathway, thereby establishing a mature vascular network. Furthermore, 3D imaging illustrated dense PGP9.5⁺ nerve fibers in close association with regenerated blood vessels in the MpGel‐NIR cohort, indicating the reconstruction of the neurovascular niche. This structural regeneration resulted in functional recovery, as MpGel‐NIR reinstated mechanical nociception (verified by behavioral pain thresholds) and upregulated the mechanosensitive ion channel PIEZO2 in regenerated nerves, essential for protective sensation.^[^ ^111^ ^]^ Similarly, Qiao et al. demonstrated that TiO_2_/Bi_2_S_3_ quantum dot nanotubes (TBNTs) enhance photocurrent generation when integrated into a dual‐crosslinked collagen/hyaluronic acid hydrogel (TBCHA) for a photoelectrically active wound dressing^[^ ^108^ ^]^ (**Figure** **8**A). In vitro, photostimulation of TBCHA increased NIH‐3T3 and PC12 cell metabolic activity, enhancing ATP production and Ca^2^⁺ influx. This stimulation further promoted fibroblast contractility and neural differentiation, indicated by increased β3‐tubulin. Additionally, it polarized macrophages towards an anti‐inflammatory phenotype. In another study, Tan et al. demonstrated a self‐powered smart patch (PRG‐G‐C) that combined a piezoelectric polarized β‐phase PVDF film with a conductive reduced graphene oxide‐gelatin methacryloyl matrix, providing programmed biological‐electrical signals for enhanced nerve regeneration^[^ ^109^ ^]^ (Figure 8C). The patch produced endogenous electrical stimulation through piezoelectric transduction resulting from mechanical deformation. As a result, CXCL12 was rapidly released to recruit BMSCs to the wound site, while ginseng‐derived ELVs (G‐Exos) were released in a sustained manner to promote neural differentiation. In vivo, PRG‐G‐C restored functional sensation, evidenced by restored scratching behavior in an itching model. Lastly, an ECM‐based conductive interpenetrating network hydrogel, incorporating an oxidized chondroitin sulfate (OCS)/gelatin methacryloyl (GM) network with polypyrrole nanoparticles, was established by Fan et al., demonstrating notable effectiveness in diabetic wound healing and neurovascular regeneration. The hydrogel displayed robust tissue adhesion through aldehyde‐amino group crosslinking and exhibited enhanced hemostatic capabilities in a liver injury model, enabling swift wound closure. Mechanistically, the hydrogel's conductivity facilitated intracellular calcium influx in HUVEC PC12 cells, thus activating the MEK/ERK and PI3K/AKT signaling pathways, which are critical for angiogenesis and neurogenesis.^[^ ^112^ ^]^

In bone and muscle repair models, conductive nanocomposite hydrogels harness multifunctional nanomaterials to drive coordinated neurogenic and osteogenic differentiation, thereby enabling fully functional tissue regeneration. A photosensitive conductive hydrogel (GBM), incorporating magnesium‐modified black phosphorus (BP@Mg) within a GelMA matrix, was developed by Jing et al. for multifunctional applications in infected bone defect repair via innervated regeneration. The nanocomposite demonstrated photothermal and photodynamic antibacterial properties under light, effectively resolving infection. In vivo analysis of GBM‐treated infected skull defects revealed enhanced neural network reconstruction, as indicated by increased CGRP⁺ nerve fibers and NGF expression at 8 weeks postimplantation. This innervation was directly associated with improved osteogenesis, as micro‐CT analysis indicated a significantly larger new bone volume in the GBM group, while histological examination revealed a high density of osteoblasts and increased osteocalcin expression. Moreover, the conductive network and bioactive Mg^2^⁺ release synergistically facilitated SC‐mediated axonal guidance and neuro‐osteogenic coupling, with CGRP⁺ fibers closely associated with new bone formation.^[^ ^116^ ^]^ In a similar study, Integrated copper ion‐modified germanium phosphorus nanosheets (GeP@Cu) in a GelMA matrix exhibited tri‐functional efficacy for bone defect repair by promoting neural regeneration, angiogenesis, and osteogenesis^[^ ^115^ ^]^ (Figure 8D). GelMA/GeP@Cu in rat calvarial defects enhanced neuro‐vascularized bone regeneration synergistically. Micro‐CT analysis indicated a reduced bone defect area and increased total vessel volume in the GelMA/GeP@Cu group, while immunohistochemical studies showed dense sensory innervation and elevated osteogenic markers. Additionally, GeP@Cu nanosheets demonstrated antibacterial properties, preventing infections, while the conductive scaffold supported neuro‐osteogenic coupling, with regenerated nerves and vessels spatially linked to newly formed bone. In addition, Wang et al utilized a Mo_2_Ti_2_C_3_ MXene/gelatin/chitosan hydrogel composed of 2D MXene nanosheets as a conductive nanocomposite to promote innervation. The remarkable electrical conductivity of MXene provided bioelectrical signals that mimicked the native neural microenvironment, facilitating the directional growth and differentiation of NSC and enhancing the expression of essential neurotrophic factors (NGF, BDNF) in local cells, thereby creating a neuro‐supportive environment, as indicated by increased Tuj‐1 and 5‐HT markers.^[^ ^113^ ^]^ Using the same class of material for addressing volumetric muscle loss (VML), Zheng et al. utilized a redox‐responsive niobium carbide MXene nanosheet within a multifunctional hydrogel matrix (comprising oxidized pullulan[O‐Pu] and dithiodipropionate dihydrazide [TPH] polymers) to achieve enhanced innervation^[^ ^117^ ^]^ (Figure 8F). Its high electrical conductivity mimicked the endogenous bioelectrical cues, promoting in situ neural differentiation of MSCs and guiding neurite outgrowth without external stimulation. Concurrently, the scaffold's redox‐responsive degradation ensured alleviation of oxidative stress, enabling a pro‐regenerative microenvironment that protects nascent nerves and supports M2 macrophage polarization.

**Table 5:** Summary of studies utilizing CNHs for PNS applications, detailing the materials used, fabrication techniques, conductivity, differentiation/ES settings, and type of cells. (Note: Abbreviations are explained in Sections S1.1. in the Supporting Information).

**Hydrogel Backbone**	**Nanomaterial (Dimension)**	**Fabrication & Integration Method**	**Highest Conductivity [S cm^−1^]**	**Differentiation Strategy**	**ES Setting**	**Type of Cell**	**Key Outcomes**	**Ref**.
Gelatin	CNT (1D)	Covalent crosslinking ‐ OP1	3.4×10^−1^	C	—	Cell line (NE‐4C)	MNH hydrogels with CNTs supported NE‐4C stem cell proliferation and differentiation, with conductivity enhancing cell spreading and neural stem cell viability. The results highlight the potential of conductive hydrogels and antimicrobial peptides in fostering neurogenesis in epithelial tissues.	[107]
Collagen / HA	CNT / Bi_2_S_3_ (1D)	PPM/ self‐assembly ‐ OP1	—	C+N+E	**OWS** [**Parameters not reported,** **Duration**: 3.5 h over 7 d]	Cell line (PC12) / Fibroblast (NIH‐3T3)	TiO2/Bi_2_S_3_ QD nanotubes exhibited high photoelectric conversion under red‐light excitation. The TBCHA hydrogel promoted nerve reinnervation and skin repair in deep burn models. Electrical activation of L‐VGCCs may induce neuronal differentiation and fibroblast activation, while mechanical stress from fibroblasts guided nerve fiber growth and ECM scaffold formation.	[108]
Gelatin	rGO (2D)	PPM ‐ OP1	—	C	—	Stem cells (MSCs)	The self‐powered PRG‐G‐C smart patch accelerated nerve regeneration and sensation restoration within 23 d. Integrating a piezoelectric film and conductive gel, it provides electrical stimulation and controlled biological cue release.	[109]
Alginate	PEI, GO (0D, 2D)	Extrusion Bioprinting ‐ OP4	—	C	—	Stem cells (MSCs)	SDF‐1α is released from the hydrogel to recruit BMSCs from the bone marrow. The recruited BMSCs over‐express MMP‐2 to hydrolyze the cGP‐bFGF crosslinker. The released GO‐PEI‐bFGF delivers pDNAs‐bFGF into BMSCs, which then differentiate into neurons in rat skin. Figure 8B	[110]
PAAm / Chitosan	rGO (2D)	Covalent crosslinking ‐ OP1	—	C	—	Schwann cells	Behavioral tests showed significant restoration of mechanical nociception in treated mice, a critical sensory function of the skin .immunofluorescence analysis revealed a marked increase in PIEZO2 expression in the treated groups.	[111]
GelMA / Oxidized chondroitin sulfate	PPy (0D)	PPM ‐ OP1	3.12×10^−3^	C	—	Cell line (PC12)	The ECM‐based conductive hydrogel accelerates diabetic wound healing by promoting neurovascular regeneration and collagen deposition. It exhibits both angiogenic and neurogenic potential, with the GM‐OCS‐P hydrogel enhancing intracellular Ca2+ levels in HUVECs and PC12 cells. This activation stimulates protein phosphorylation in the MEK/ERK and PI3K/AKT pathways, facilitating neurovascular regeneration.	[112]
Gelatin /chitosan	MXene (2D)	Covalent crosslinking ‐ OP1	—	C	—	Cell line (C2C12)	The Mo_2_Ti_2_C_3_ MXene hydrogel promotes neurogenesis by upregulating NGF and BDNF, essential for neuronal survival and differentiation in bone defect repair. It enhances new neuron formation, evidenced by increased Tuj‐1 and serotonin (5‐HT) expression, supporting nerve tissue regeneration in the bone microenvironment.	[113]
Gelatin	rGO (2D)	Extrusion bioprinting ‐ OP1	—	C	—	Schwann cells	Results showed a significant increase in NGF, PMP22, and Krox20 markers on the rGO/GelMA scaffolds compared to the control, indicating that rGO promotes neural differentiation, specifically aiding in the maturation of Schwann cells (SCs) and myelination simultaneous to bone differentiation.	[114]
Gelatin	GeP (2D)	PPM ‐ OP1	4.00×10^−3^	C	—	Stem cells (NSCs)	The GelMA/GeP@Cu hydrogel demonstrated strong antibacterial activity, effectively preventing infections through its copper ion‐modified GeP nanosheets. In vivo, the hydrogel activated sensory innervation in a rat skull defect model, aiding neuro‐vascularized bone regeneration by enhancing both nerve regeneration and angiogenesis.	[115]
GelMA	Black phosphorus (2D)	PPM ‐ OP1	10^−3^	C + N	—	Cell line (PC12)	The GBM photosensitive conductive hydrogel enhanced Schwann cell migration and function in infected bone defects through magnesium ions and conductivity. It promoted neurotrophic factor secretion, notably NGF, supporting neurite outgrowth and nerve regeneration. In vivo, it facilitated CGRP nerve fiber regeneration, improving sensory nerve function.	[116]
Pullulan	MXene (2D)	Covalent crosslinking ‐ OP1	2.00×10^−3^	C	—	Stem cells (MSCs)	The Nb2C MXene‐functionalized OPTN hydrogel scaffold enhances neurogenesis by promoting MSC differentiation into neural lineages, aiding functional recovery after volumetric muscle loss. it scavenges ROS, reducing oxidative stress and inflammation to support neurogenesis in muscle tissue.	[117]
Matrigel	Mxene (2D)	Thermal gelation ‐ OP1	7.3 ×10^−5^	C + N	—	Stem cells (cochlea organoids)	Ti_3_C_2_T_x_ MXene in Matrigel enhances cochlear organoid differentiation into functional hair cells, promoting auditory neurogenesis. The MXene‐Matrigel composite activates the mTOR pathway, supporting hair cell survival and differentiation. Regenerated hair cells exhibit superior electrophysiological properties and improved innervation with spiral ganglion neurons, fostering functional neurogenesis.	[118]
GelMA	Carbon nanotube sheets (2D)	PPM ‐ OP1	—	C + N	—	Spiral ganglion neurons	Spiral ganglion neurons on the GelMA‐SACNT scaffold showed improved orientation, longer neurites, and larger axon growth cones, highlighting the material's potential in promoting neuronal growth and restoring hearing function.	[119]

In cochlear applications, conductive hydrogels have been tailored to promote auditory neurogenesis and restore hearing function by enhancing the differentiation of cochlear organoids and spiral ganglion neurons. Zhang et al. synthesized a Ti_3_C_2_T_x_ MXene‐Matrigel composite hydrogel that notably improved cochlear organoid development by facilitating hair cell regeneration, maturation, and synaptic connections^[^ ^118^ ^]^ (Figure 8E). The MXene nanocomposite enhanced Matrigel's properties, augmenting hydrophilicity and bioelectrical conductivity to establish a pro‐differentiation microenvironment. This process activated the mTOR‐HIF1α signaling pathway, leading to hair cell differentiation, as evidenced by increased Atoh1‐GFP⁺ progenitor cells and Myo7a⁺ mature hair cells. Furthermore, the regenerated hair cells displayed electrophysiological characteristics akin to native cells, demonstrated by comparable resting membrane potential (≈−40.2 mV), voltage responses, and action potentials triggered by membrane depolarization. Importantly, MXene‐Matrigel promoted functional reinnervation, fostering synapse‐like interactions between hair cells and spiral ganglion neurons (SGNs) through the upregulation of presynaptic and postsynaptic markers. In another study, a GelMA‐SACNT composite scaffold integrated super‐aligned carbon nanotube sheets (SACNTs)  to create an electroconductive, topographically guided platform for SGNs regeneration. The scaffold leveraged the anisotropic alignment of SACNTs (30–50 nm diameter) to provide directional cues, while GelMA ensured biocompatibility and porous structural support (≈10 µm pores). This nanocomposite design enhanced SGN adhesion and neuritogenesis: SGNs cultured on GelMA‐SACNT exhibited longer neurites, larger growth cone areas, and filopodia length compared to controls, with neurites aligning along the nanotube axis. By merging SACNT‐derived topographical guidance with GelMA‐mediated bioactivity, this scaffold drove the formation of anisotropic, electrophysiologically active SGN networks—applicable for reconnecting auditory pathways in sensorineural hearing loss.^[^ ^119^ ^]^

## 4 Discussion

The application distribution indicates a strategic emphasis on critical clinical needs and translational potential. The higher occurrence of CNS applications (*n* = 53, 42.4%) compared with PNS (*n* = 39, 31.2%) underscores the severity of the former condition and the unique technical challenges making them amenable to biomaterial interventions. The significant emphasis on SCI (*n* = 42) points to not merely a research preference but an alignment of clinical necessity, technical feasibility, and regulatory pathways, positioning it as an optimal ground for CNH strategies.^[^ ^120^ ^]^ Additionally, the brain's intricate 3D structure, regional heterogeneity, and complex vasculature present technical challenges to biomaterial‐based interventions, accounting for the relatively low number of brain‐targeted CNH investigations (*n* = 9) despite pressing clinical need.^[^ ^121^ ^]^ Most research has focused on acute injuries, such as traumatic brain injury and stroke, which share inflammatory and necrotic mechanisms with SCI, indicating that current CNH platforms are suited for early‐stage injury repair. The scarcity of research on chronic neurodegenerative diseases offers a crucial opportunity to improve the clinical relevance of CNH technologies. Among the applications in PNS, the predominance of sciatic nerve repair studies (*n* = 20) capitalizes on the inherent regenerative capabilities of the PNS, combined with surgical convenience, well‐established functional assessments, and its relatively uncomplicated anatomical configuration,^[^ ^122^ ^]^ all of which collectively facilitate the design and assessment of biomaterials. However, this emphasis may overlook the unique anatomical and functional complexities of other peripheral nerves, which likely demand tailored CNH strategies.^[^ ^123^ ^]^ On the other hand, the expanding scope of PNS‐focused research, encompassing neurogenesis within epithelial tissues and osseous structures (each *n* = 6), cochlear and auditory applications (*n* = 2), and muscular systems (*n* = 1), highlights an increasing recognition of the essential contribution of innervation to tissue regeneration and functionality. The development of biomaterials that simultaneously facilitate tissue repair alongside neural integration is an avenue toward the realization of fully integrated regenerative therapies.^[^ ^124^ ^]^

NTE requires materials with a balance between electrical conductivity, biocompatibility, mechanical compliance, and ease of processing. Carbon‐based nanomaterials make up 36.8% of the studies due to their excellent electrical properties and functional versatility. Their high aspect ratio leads to efficient conductive networks, and the surface groups on graphene derivatives enhance dispersion and bioactive molecule attachment. Still, in the absence of surface modification, they are prone to aggregation, which may lead to variable cellular responses or induce toxicity, thus underscoring the necessity for careful functionalization approaches.^[^ ^125^ ^]^ On the other hand, Metal‐based nanostructures (24.0%) and conductive polymers (16.0%) offer specialized functionalities and integration modes. Iron oxide and gold nanoparticles provide magnetic navigation, improved imaging contrast, and notable electrical conductivity, with known safety profiles: iron oxide is biodegradable via standard metabolic processes, while gold's inertness ensures stability. Conductive polymers demonstrate capabilities in in situ hydrogel polymerization, forming interpenetrating networks and consistent electrical performance; nonetheless, their long‐term biostability and potential inflammatory byproducts require additional refinement.^[^ ^29^, ^126^ ^]^ Beyond the traditionally recognized categories, novel materials—specifically MXenes (*n* = 7), semiconductor nanostructures (8.0%), and piezo‐/magnetoelectric ceramics (4.0%)—are broadening the repertoire of CNHs.  MXenes possess metal‐like conductivity alongside hydrophilicity and adjustable surface terminations; however, their biocompatibility and degradation mechanisms are still subjects of ongoing research.^[^ ^127^ ^]^ Semiconductors, including black phosphorus and silicon, provide wavelength‐specific, optically induced stimulation alongside intrinsic biodegradability, yet they are susceptible to rapid degradation via environmental factors.^[^ ^128^ ^]^ Lastly, ceramics such as barium titanate facilitate wireless, mechanically or magnetically triggered electrical signals, thereby creating opportunities for non‐invasive neural modulation, although their inherent brittleness and complexity of integration present obstacles to seamless incorporation within hydrogels.^[^ ^129^ ^]^ Each category has unique advantages and disadvantages, emphasizing the application‐oriented material selection in the development of CNHs.

The cell sources in CNH research are skewed considerably toward rodent‐derived models, reflecting their ease of use and well‐established protocols but raising concerns about translational relevance. NSCs represent the predominant option (n = 40), but their inherent heterogeneity and the necessity for specific growth‐factor‐enriched media may pose challenges to reproducibility and scalability.^[^ ^130^ ^]^ The absence of human iPSCs in CNH research is a critical gap that undermines its translational relevance and clinical impact. Integrating patient derived iPSCs could overcome limitations of differences between human and rodent models while enabling personalized disease modeling, pharmacogenomic studies, and reduced immunogenicity in regenerative approaches.^[^ ^131^ ^]^ However, extended iPSC differentiation timelines demand materials with stable electrical and mechanical properties that resist degradation and support prolonged maturation, and require careful optimization of biomaterial–stimulation compatibility. Despite these challenges, iPSCs have proven effective for generating diverse neuron types, 3D brain‐like organoids, and CRISPR engineered isogenic models.^[^ ^132^, ^133^ ^]^ Systematic incorporation and investigation of iPSC‐derived neural cells into CNH platforms has the potential to advance personalized NTE greatly.

Among studies on SCI repair, dual‐functionality platforms emphasize that effective repair must tackle both immediate electrical disconnection and subsequent adverse microenvironments. SCI begins with mechanical trauma disrupting neural pathways and signal conduction; however, ongoing secondary processes, such as reactive oxygen species production, inflammatory cell infiltration, and glial scar formation, exacerbate damage and hinder regeneration. This understanding acknowledges that effective spinal cord repair necessitates not only the reinstatement of electrical signal transmission but also the active modulation of oxidative stress, inflammatory responses, and excessive glial proliferation.^[^ ^134^ ^]^ Moreover, wireless stimulation technologies represent an advancement in SCI treatment by transforming how electrical therapeutic interventions are delivered and by eliminating many of the complications and limitations of traditional implanted electrode systems such as risks of tissue damage, inflammatory responses, electrode degradation, and infection.^[^ ^135^ ^]^ Main modalities include magnetic induction, where embedded nanoparticles convert applied fields into localized currents; capacitive coupling, which noninvasively transfers energy across tissue via electric field interfaces; and piezoelectric stimulation, in which body movements drive continual therapeutic currents. Each approach bypasses the need for rigid implants and offers customizable, on‐demand neuromodulation suited to the dynamic environment of spinal cord repair. Clinically, when integrated with advanced imaging and neuro‐monitoring, wireless platforms can help form closed‐loop systems that track electrical activity, tissue regeneration, and functional recovery while autonomously adjusting stimulation for optimal results. This precision and adaptability promise significant progress towards truly intelligent and integrated therapeutic strategies for SCI.

Neurological disorders, including TBI, stroke, and neurodegenerative diseases such as Alzheimer's and Parkinson's, pose significant challenges for modern medicine.^[^ ^136^ ^]^ The brain's complex nature and restricted regenerative abilities demand novel therapeutic approaches. Instead of attempting to utilize a uniform scaffold for all forms of injuries, studies have emphasized the necessity of customizing material composition, architectural configuration, and bioactive properties to align with the cellular, molecular, and tissue‐level disease features. PD, for example, involves dopaminergic neuron degeneration, oxidative stress, neuroinflammation, and protein misfolding^[^ ^137^ ^]^; stroke leads to ischemia–reperfusion injury, cavity formation, and inflammation; and traumatic brain injury features mechanical disruption and secondary oxidative and inflammatory damage.^[^ ^138^ ^]^ Research has focused on developing hydrogels, nanoparticles, and composite scaffolds tailored to the specific redox environment, mechanical properties, and electrical requirements of each condition. Xu et al. demonstrated the effectiveness of tannic acid‐modified gold nano‐crosslinker hydrogels in Parkinson's disease models by scavenging reactive oxygen species and enhancing electrical conductivity for neuroprotection. This self‐healing hydrogel improved inflamed neural stem cell conditions, increased tyrosine hydroxylase‐positive neuron populations, and enhanced motor function in PD rats.^[^ ^74^ ^]^ In contrast, Zhang et al. addressed the adverse conditions post‐stroke using GelMA/PEDOT:PSS hybrids, where the hydrogel matrix minimized apoptosis when cocultured with oxygen‐glucose deprivation/reperfusion neurons, and PEDOT:PSS maintained conductivity while integrating electrical stimulation with anti‐inflammatory signals.^[^ ^79^ ^]^

The recognition that neural regeneration is intimately connected to the regeneration of other tissue types has emerged as a central theme in CNH for PNS applications, affecting how researchers approach complex regenerative challenges. In bone regeneration, for example, neural innervation is observed to precede and direct vascularization and ossification, with nerve‐derived signals regulating the regenerative cascade. Jing et al. emphasized this temporal priority in their work on infected bone defect work, showing that nerve regeneration contributed to the subsequent vascularization, ossification, and mineralization processes.^[^ ^116^ ^]^ Similarly, Zhang et al. illustrated this principle in their neuralized bone regeneration study, showing that Schwann cells and bone marrow mesenchymal stem cells engage in synergistic interactions that promote the differentiation and function of both the neural and bone tissue components.^[^ ^114^ ^]^ Calcitonin gene‐related peptide (CGRP) illustrates the multifunctional role of neural signaling molecules in tissue regeneration. Initially identified for pain signaling, CGRP is now recognized as a fundamental regulator of diverse physiological processes.^[^ ^139^ ^]^ Jing et al. demonstrated the enhanced CGRP+ nerve fiber regeneration in infected bone defect models, emphasizing the activation of this multifaceted signaling pathway in multi‐tissue regeneration.^[^ ^116^ ^]^ The implications of neural‐tissue crosstalk are extended to design principles for CNH systems. Rather than the singular pursuit of material optimization for neural compatibility, successful CNH platforms must consider the needs of disparate tissue types and the changing nature of these needs throughout the regenerative process. In their work related to diabetic wound healing, Fan et al. carefully designed conductive interpenetrating network hydrogels to address both inadequate vascularization and severe peripheral neuropathy through synchronized neurovascular regeneration.^[^ ^112^ ^]^

Finally, the intersection of photonics and NTE has resulted in a versatile CNH approach in which light activated stimulation delivers spatially and temporally precise cues. Progress has been made through advanced nanomaterial engineering that facilitates red‐light activation with enhanced conversion efficiency. Qiao et al. developed TiO_2_/Bi_2_S_3_ heterojunction nanotubes, which exhibited an impressive 66‐fold enhancement of photocurrent (9.22 µA cm^−^^2^) over traditional TiO_2_ nanotubes while providing safe red‐light activation.^[^ ^108^ ^]^ This strategy not only allowed deep tissue penetration but also reduced photothermal damage and cellular stress associated with shorter wavelengths. The clinical potential of photoelectric stimulation goes well beyond simple electrical activation by using noninvasive light to deliver precise, controlled currents. The same photonic trigger can further accommodate complementary therapeutic modalities such as photothermal antimicrobial efficacy or photodynamic remodeling, thus giving rise to a multiplexed therapeutic strategy within a single application. Jing et al. demonstrated this in infected bone defects: near infrared irradiation of BP@Mg hydrogels provided antibacterial photothermal therapy to aid neural regeneration.^[^ ^116^ ^]^

## 5 Limitations and Future Perspectives

This systematic scoping review synthesizes contemporary research on conductive nanocomposite hydrogels for neural tissue engineering. Our search was limited to published, peer‐reviewed literature in major databases, and we did not search for grey literature, which may have omitted some very recent or ongoing work. Moreover, our review deliberately excluded constructs with no cellular component or tissue formation, which has resulted in the lack of discussion regarding advancements in other areas employing CNHs, such as neural interfaces for monitoring or biosensing applications. Moreover, owing to the substantial number of SCI studies, not all could be discussed and summarized within the main results section; readers are therefore referred to the supplementary Information for further details. As a scoping review, we did not engage in a formal evaluation of study quality or risk of bias, thereby limiting our ability to differentiate between high‐quality and low‐quality findings. Additionally, the variability observed in reporting methodologies precluded conducting a quantitative synthesis (e.g., meta‐analysis), thereby adhering only to a narrative approach.

The future of CNHs in NTE relies on a combination of new materials, multifunctional designs, innovative production processes, and stringent safety and regulatory demands. By identifying new materials and employing sophisticated design strategies, researchers can fabricate multifunctional CNHs that exhibit drug delivery, real‐time biosensing, and dynamic physiological responses. These CNHs can sense and react to the dynamic microenvironment of neural injury and respond to stimuli such as pH, temperature changes, light, magnetic fields, and injury‐specific biochemical cues. By combining sensing components and actuation modules in a single network, bioelectronic interfaces can be designed to bridge the gap between tissue conditions and therapeutic responses. A future direction involves the development of smarter, responsive systems that can integrate sensing capabilities to guide therapeutic output, moving towards closed‐loop bioelectronic therapies. Furthermore, the intersection of advanced fabrication techniques with CNHs has the potential to propel the field further. 3D and 4D bioprinting can fabricate intricate, high‐resolution structures with engineered geometry, stiffness, and electrical properties, whereas microfluidic fabrication can produce hydrogel microspheres or aligned networks of fibers that replicate natural anisotropy and augmented cell guidance. Additionally, injectable CNHs combine moldable, shear‐thinning behavior with high mechanical integrity, helping to fill irregular gaps resulting from discrete injuries. Together, these technologies can create patient‐specific implants that mimic the intricate architecture of the nervous system.

Advancing the integration of iPSCs with CNHs technologies can mark a significant development in NTE. This approach offers an ethical and personalized source of cells that can transform into neurons, astrocytes, and oligodendrocytes. By incorporating these cells, researchers can work on creating a biomimetic environment that provides essential electrical signals to steer differentiation, encourage neurite extension, and support the formation of functional synapses. Future research may focus on refining the composition and structure of CNHs to guide iPSCs toward specific neuronal or glial pathways and ensure their long‐term viability and integration into damaged neural tissue.

Regulatory approval will only follow if there are robust manufacturing processes that provide batch‐to‐batch consistency, scalability in cost‐effective terms, and conformance to applicable medical device standards. For CNHs to be of clinical benefit, the rates of degradation must be equal to or slightly greater than the rate at which tissue heals; losing structural support too rapidly and materials remaining as residues for too long can induce chronic inflammation. A systematic investigation of metabolite clearance, interaction with the blood–brain barrier, and long‐term nanotoxicity over long periods, especially concerning conductive fillers, is called for. In addition, the balance between conductivity and biocompatibility is of utmost importance; high levels of conductive fillers can provoke inflammation, whereas high ionic content might be toxic to encapsulated cells. Recently utilized biodegradable conductive materials, such as black phosphorus, germanium phosphide, MXenes, and polymer‐oligomer conjugates, hold promise for providing conductivity, degradability, and cytocompatibility in a single step.

## 6 Conclusion

Conductive nanocomposite hydrogels have emerged as versatile, multifunctional platforms for neural repair, aiming to integrate electrical conductivity with antioxidant, immunomodulatory, and controlled‐release drug delivery functions to create synergistic microenvironments that counter oxidative stress, suppress inflammation, and reestablish bioelectrical signaling. In CNS, applications ranging from spinal cord injury to Parkinson's disease and stroke leverage tailored hydrogel compositions—incorporating anti‐inflammatory cues, dynamic stiffness modulation, and cell supportive matrices—to promote stem cell survival, remyelination, and cavity filling. In PNS, magnetically responsive scaffolds, reinforced by aligned fibers and biomimetic topographies, guide axonal regeneration and targeted cell delivery, while wireless, self‐powered piezoelectric, magnetoelectric, and photosensitive systems deliver on‐demand stimulation. Also, applications extend to neurovascular niche reconstruction in diabetic wounds, neuro‐osteogenic coupling in bone repair, redox‐responsive innervated muscle regeneration, and cochlear haircell and spiral ganglion neuron recovery. In various settings, the integration of electrical, topographical, and biochemical signals has been demonstrated to influence stem cell development and enhance the expression of neuronal markers. These hydrogels, which respond to multiple stimuli, thus offer a potentially versatile and promising clinical approach for the comprehensive therapeutic goals of neural tissue engineering.

## Conflict of Interest

The authors declare no conflict of interest.

## Author Contributions

M.M.: Framework Development, Research Design, Data Extraction, Data Analysis, Data Visualization, Draft Composition, Revision. B.O.: Data Extraction, Data Visualization, Draft Composition, Revision and Editing. A.B.B.: Data Extraction, Draft Composition. R.Y.: Data Extraction, Draft Composition. M.M.: Data Extraction, Draft Composition. C.B.U.: Framework Development, Research Design, Project Management, Supervision, Validation, Revision. I.T.O.: Project Management, Supervision, Validation, Revision & Editing.

## Supporting information



Supporting Information

## Data Availability

All pertinent information is publicly available in the referenced OSF repository at osf.io/ptw3n.^[^ ^2^ ^]^ Preregistered protocol DOI: https://doi.org/10.17605/OSF.IO/DH4PX. Extracted raw data: Associated project/Files/ Reconciled data and extraction form. Excluded studies log: Associated project/Files/ Excluded studies with reasons. Analysis & visualization code: Associated project/Files/Data analysis files

## References

[1] A. C. Tricco , E. Lillie , W. Zarin , K. K. O'Brien , H. Colquhoun , D. Levac , D. Moher , M. D. J. Peters , T. Horsley , L. Weeks , S. Hempel , E. A. Akl , C. Chang , J. McGowan , L. Stewart , L. Hartling , A. Aldcroft , M. G. Wilson , C. Garritty , S. Lewin , C. M. Godfrey , M. T. Macdonald , E. V. Langlois , K. Soares‐Weiser , J. Moriarty , T. Clifford , Ö. Tunçalp , S. E. Straus , Ann. Intern. Med. 2018, 169, 467 30178033 10.7326/M18-0850

[2] M. Moghaddasi , B. Oktay , C. Üstündağ , Hydrogel‐Based Conductive Nanocomposites in Neural Tissue Engineering 2024. 10.17605/OSF.IO/DH4PX

[3] M. Ouzzani , H. Hammady , Z. Fedorowicz , A. Elmagarmid , Syst. Rev. 2016, 5, 210. 27919275 10.1186/s13643-016-0384-4 PMC5139140

[4] P. J. Bazira , Surg. Oxf. 2021, 39, 451.

[5] A. Maria , A. Mallamaci , in The Role of Natural Antioxidants in Brain Disorders (Eds: A. Imran , G. Hussain ), Springer International Publishing, Cham 2023, pp. 25–48.

[6] J. Moskow , B. Ferrigno , N. Mistry , D. Jaiswal , K. Bulsara , S. Rudraiah , S. G. Kumbar , Bioactive Materials 2019, 4, 107. 30723843 10.1016/j.bioactmat.2018.09.001 PMC6351356

[7] S. Mantha , S. Pillai , P. Khayambashi , A. Upadhyay , Y. Zhang , O. Tao , H. M. Pham , S. D. Tran , Materials 2019, 12, 3323. 31614735 10.3390/ma12203323 PMC6829293

[8] M. F. Akhtar , M. Hanif , N. M. Ranjha , Saudi Pharm. J. 2016, 24, 554. 27752227 10.1016/j.jsps.2015.03.022 PMC5059832

[9] P. Madhusudanan , G. Raju , S. Shankarappa , J. R. Soc. Interface 2020, 17, 20190505, 31910776 10.1098/rsif.2019.0505 PMC7014813

[10] H. Zhao , M. Liu , Y. Zhang , J. Yin , R. Pei , Nanoscale 2020, 12, 14976. 32644089 10.1039/d0nr03785k

[11] C. Dannert , B. T. Stokke , R. S. Dias , Polymers 2019, 11, 275. 30960260 10.3390/polym11020275 PMC6419045

[12] Z. Ahmad , S. Salman , S. Ali Khan , A. Amin , Z. Ur Rahman , Y. O. Al‐Ghamdi , K. Akhtar , E. M. Bakhsh , S. B. Khan , Materials 2019, 12, 3323.31614735

[13] J. T. Seil , T. J. Webster , WIREs Nanomedicine Nanobiotechnology 2010, 2, 635. 20730786 10.1002/wnan.109

[14] Bioinspired micro‐ and nano‐structured neural interfaces, PubMed, 2022, https://pubmed.ncbi.nlm.nih.gov/35947922/ (accessed: July, 2025). 10.1088/1361-6528/ac8881 35947922

[15] Advances in Conductive Hydrogel for Spinal Cord Injury Repair and Regeneration, *PubMed* , 2023, https://pubmed.ncbi.nlm.nih.gov/38084124/ (accessed: July, 2025). 10.2147/IJN.S436111 PMC10710813 38084124

[16] J. Senanayake , H. G. Sundararaghavan , Bioelectricity 2024, 6, 13.

[17] A. Raspa , F. Gelain , Curr. Neuropharmacol. 2021, 19, 2110. 33176654 10.2174/1570159X18666201111111102 PMC9185766

[18] S. Jooken , O. Deschaume , C. Bartic , Gels 2023, 9, 153. 36826323 10.3390/gels9020153 PMC9957407

[19] M. Farokhi , F. Mottaghitalab , M. R. Saeb , S. Shojaei , N. K. Zarrin , S. Thomas , S. Ramakrishna , Macromol. Biosci. 2021, 21, 2000123. 10.1002/mabi.202000123 33015992

[20] S. H. Ku , M. Lee , C. B. Park , Adv. Healthcare Mater. 2013, 2, 244. 10.1002/adhm.201200307 23184559

[21] A. D. Sontakke , S. Tiwari , M. K. Purkait , FlatChem 2023, 38, 100484.

[22] L. Liang , X. Peng , F. Sun , Z. Kong , J.‐W. Shen , Nanoscale Adv. 2020, 26, 904. 10.1039/d0na00904k PMC9419276 36133293

[23] S.‐P. Peng , M. Schachner , E. Boddeke , S. Copray , Cell. Reprogramming 2016, 18, 55. 10.1089/cell.2015.0059 26990843

[24] Recent advances in the application of MXenes for neural… : Neural Regeneration Research, https://journals.lww.com/nrronline/fulltext/2024/02000/Recent_advances_in_the_application_of_MXenes_for.4.aspx (accessed: July, 2025). 10.4103/1673-5374.379037 PMC10503607 37488875

[25] B. Guo , P. X. Ma , Biomacromolecules 2018, 19, 1764. 29684268 10.1021/acs.biomac.8b00276 PMC6211800

[26] E. Mostafavi , D. Medina‐Cruz , K. Kalantari , A. Taymoori , P. Soltantabar , T. J. Webster , Bioelectricity 2020, 2, 120. 34471843 10.1089/bioe.2020.0021 PMC8370325

[27] P. Wang , E. Zhang , D. Toledo , I. T. Smith , B. Navarrete , N. Furman , A. F. Hernandez , M. Telusma , D. McDaniel , P. Liang , S. Khizroev , Nano Lett. 2020, 20, 5765. 32639738 10.1021/acs.nanolett.0c01588

[28] L. Xia , W. Zhu , Y. Wang , S. He , R. Chai , Neural Plast. 2019, 2019, 3608386. 31737061 10.1155/2019/3608386 PMC6817925

[29] J. H. Min , M. Patel , W.‐G. Koh , Polymers 2018, 10, 1078. 30961003 10.3390/polym10101078 PMC6404001

[30] E. Sancar , B. Oktay , E. A. Özerol , Mater. Res. Express 2024, 11, 122001.

[31] Electrically conductive nanomaterials: transformative applications in biomedical engineering‐a review, PubMed, 2024, https://pubmed.ncbi.nlm.nih.gov/39389095/ (accessed: July, 2025). 10.1088/1361-6528/ad857d 39389095

[32] M. S. Vieira , A. K. Santos , R. Vasconcellos , V. A. M. Goulart , R. C. Parreira , A. H. Kihara , H. Ulrich , R. R. Resende , Biotechnol. Adv. 2018, 36, 1946. 30077716 10.1016/j.biotechadv.2018.08.002

[33] S. Wang , S. Guan , C. Sun , H. Liu , T. Liu , X. Ma , Brain Res. 2023, 1798, 148163. 36379314 10.1016/j.brainres.2022.148163

[34] C. Zhou , T. Wu , X. Xie , G. Song , X. Ma , Q. Mu , Z. Huang , X. Liu , C. Sun , W. Xu , Eur. Polym. J. 2022, 177, 111454.

[35] B. C. Heng , Y. Bai , X. Li , Y. Meng , X. Zhang , X. Deng , Smart Mater. Med. 2022, 3, 4.

[36] R. Zhu , Z. Sun , C. Li , S. Ramakrishna , K. Chiu , L. He , Exp. Neurol. 2019, 319, 112963. 31125549 10.1016/j.expneurol.2019.112963

[37] P. R. Lee , K. Lee , J. M. Park , S. Kim , S. B. Oh , Int. J. Oral Sci. 2025, 17, 45. 40461462 10.1038/s41368-025-00374-8 PMC12134352

[38] W. Zhang , Y. Yang , B. Cui , Curr. Opin. Solid State Mater. Sci. 2021, 25, 100873. 33364912 10.1016/j.cossms.2020.100873 PMC7751896

[39] J. Xie , M. R. MacEwan , X. Li , S. E. Sakiyama‐Elbert , Y. Xia , ACS Nano 2009, 3, 1151. 19397333 10.1021/nn900070z PMC2765554

[40] T. Dvir , B. P. Timko , D. S. Kohane , R. Langer , Nat. Nanotechnol. 2011, 6, 13. 21151110 10.1038/nnano.2010.246 PMC4059057

[41] B. K. K. Teo , S. T. Wong , C. K. Lim , T. Y. S. Kung , C. H. Yap , Y. Ramagopal , L. H. Romer , E. K. F. Yim , ACS Nano 2013, 7, 4785. 23672596 10.1021/nn304966z

[42] J. Luo , M. Walker , Y. Xiao , H. Donnelly , M. J. Dalby , M. Salmeron‐Sanchez , Bioact. Mater. 2022, 15, 145. 35386337 10.1016/j.bioactmat.2021.11.024 PMC8940943

[43] C. Schulte , M. Ripamonti , E. Maffioli , M. A. Cappelluti , S. Nonnis , L. Puricelli , J. Lamanna , C. Piazzoni , A. Podestà , C. Lenardi , G. Tedeschi , A. Malgaroli , P. Milani , Front. Cell. Neurosci. 2016, 10, 267. 27917111 10.3389/fncel.2016.00267 PMC5114288

[44] D. Huang , Y. Huang , Y. Xiao , X. Yang , H. Lin , G. Feng , X. Zhu , X. Zhang , Acta Biomater. 2019, 97, 74. 31400521 10.1016/j.actbio.2019.08.013

[45] Y. Zhang , Z. Wang , Q. Sun , Q. Li , S. Li , X. Li , Materials 2023, 16, 5156 37512430

[46] S. Huang , X. Hong , M. Zhao , N. Liu , H. Liu , J. Zhao , L. Shao , W. Xue , H. Zhang , P. Zhu , R. Guo , Bioeng. Transl. Med. 2022, 7, 10315. 10.1002/btm2.10315 PMC9471997 36176618

[47] In vitro neurotoxicology: an introduction, PubMed, https://pubmed.ncbi.nlm.nih.gov/21815055/ (accessed: July, 2025).

[48] M. Keremidarska‐Markova , I. Sazdova , B. Ilieva , M. Mishonova , M. Shkodrova , K. Hristova‐Panusheva , N. Krasteva , M. Chichova , Nanomaterials 2024, 14, 188. 38251152 10.3390/nano14020188 PMC10818754

[49] M. Toledano , M. Toledano‐Osorio , A. Carrasco‐Carmona , C. Vallecillo , R. Toledano , A. L. Medina‐Castillo , R. Osorio , Polymers 2020, 12, 1845. 32824577 10.3390/polym12081845 PMC7465038

[50] T. R. Ham , N. D. Leipzig , Biomed. Mater. 2018, 13, 024105. 29155409 10.1088/1748-605X/aa9bbb PMC5824690

[51] M. Liu , W. Zhang , S. Han , D. Zhang , X. Zhou , X. Guo , H. Chen , H. Wang , L. Jin , S. Feng , Z. Wei , Adv. Mater. 2024, 36, 2313672. 10.1002/adma.202313672 38308338

[52] L. Chen , W. Wang , Z. Lin , Y. Lu , H. Chen , B. Li , Z. Li , H. Xia , L. Li , T. Zhang , J. Nanobiotechnol. 2022, 20, 210. 10.1186/s12951-022-01396-8 PMC9074236 35524268

[53] B. Zhang , W. Wang , P. Gao , X. Li , L. Chen , Z. Lin , H. Chen , W. Liang , Z. Kong , D. Lin , X. Wu , T. Zhang , Adv. Healthcare Mater. 2024, 13, 2304300. 10.1002/adhm.202304300 38589053

[54] G. Agarwal , N. Kumar , A. Srivastava , Mater. Sci. Eng. C Mater. Biol. Appl. 2021, 118, 111518. 33255073 10.1016/j.msec.2020.111518

[55] E. A. Kiyotake , E. E. Thomas , H. B. Homburg , C. K. Milton , A. D. Smitherman , N. D. Donahue , K.‐M. Fung , S. Wilhelm , M. D. Martin , M. S. Detamore , J. Biomed. Mater. Res., Part A 2022, 110, 365. 10.1002/jbm.a.37294 PMC9529015 34390325

[56] C. Wu , S. Chen , T. Zhou , K. Wu , Z. Qiao , Y. Zhang , N. Xin , X. Liu , D. Wei , J. Sun , H. Luo , L. Zhou , H. Fan , ACS Appl. Mater. Interfaces 2021, 13, 52346. 34699166 10.1021/acsami.1c14679

[57] W. Kong , Y. Zhao , Y. Xiaoyu , J. Chen , Y. Chen , Z. Zhao , X. Chen , F. Wang , C. Fu , Ceram. Int. 2023, 49, 20623.

[58] S. Yao , Y. Yang , C. Li , K. Yang , X. Song , C. Li , Z. Cao , H. Zhao , X. Yu , X. Wang , L. N. Wang , Bioactive Materials 2024, 35, 534. 38414842 10.1016/j.bioactmat.2024.01.021 PMC10897856

[59] C. Gao , Y. Li , X. Liu , J. Huang , Z. Zhang , Chem. Eng. J. 2023, 451, 138788.

[60] B. Yang , C. Liang , D. Chen , F. Cheng , Y. Zhang , S. Wang , J. Shu , X. Huang , J. Wang , K. Xia , L. Ying , K. Shi , C. Wang , X. Wang , F. Li , Q. Zhao , Q. Chen , Bioactive Materials 2022, 15, 103. 35386356 10.1016/j.bioactmat.2021.11.032 PMC8941182

[61] C. Xu , Y. Chang , P. Wu , K. Liu , X. Dong , A. Nie , C. Mu , Z. Liu , H. Dai , Z. Luo , Adv. Funct. Mater. 2021, 31, 2104440.

[62] K. Zhang , J. Li , J. Jin , J. Dong , L. Li , B. Xue , W. Wang , Q. Jiang , Y. Cao , Mater. Des. 2020, 196, 109092.

[63] Q. Zhang , L. Zhang , W. Weng , X. Qiu , Y. Zhuang , H. Wang , F. Wei , J. Dai , H. Shen , Y. Chen , ACS Mater. Lett. 2024, 6, 3443.

[64] P. Wu , P. Chen , C. Xu , C. Mu , X. Zou , K. Yang , Y. Xu , X. Li , X. Li , Z. Liu , Z. Wang , Z. Luo , Nano Energy 2024, 130, 110123.

[65] Z. Li , X. Wang , Z. Zhao , Y. Liu , Chem. Eng. J. 2024, 493, 152238.

[66] K. Liu , Y. Wang , X. Dong , C. Xu , M. Yuan , W. Wei , Z. Pang , X. Wu , H. Dai , Small 2024, 20, 2310194. 10.1002/smll.202310194 38279612

[67] F. Xue , T. Liu , X. Liu , K. Chen , L. Duan , G. Gao , Eur. Polym. J. 2022, 173, 111225.

[68] A. Serafin , M. C. Rubio , M. Carsi , P. Ortiz‐Serna , M. J. Sanchis , A. K. Garg , J. M. Oliveira , J. Koffler , M. N. Collins , Biomater. Res. 2022, 26. 10.1186/s40824-022-00310-5 PMC9682832 36414973

[69] J. Cai , H. Zhang , Y. Hu , Z. Huang , Y. Wang , Y. Xia , X. Chen , J. Guo , H. Cheng , L. Xia , W. Lu , C. Zhang , J. Xie , H. Wang , R. Chai , J. Nanobiotechnol. 2022, 20, 460. 10.1186/s12951-022-01669-2 PMC9617371 36307790

[70] S. Song , Y. Li , J. Huang , S. Cheng , Z. Zhang , Biomater. Adv. 2023, 148, 213385. 36934714 10.1016/j.bioadv.2023.213385

[71] P. Wu , C. Xu , X. Zou , K. Yang , Y. Xu , X. Li , X. Li , Z. Wang , Z. Luo , Adv. Mater. 2024, 36, 2310483. 10.1002/adma.202310483 38198600

[72] Q. Liu , W. Wang , H. Yang , Y. Wang , Y. Shi , Y. Chen , D. Luo , D. Guo , Adv. Funct. Mater. 2024, 34, 2406376.

[73] C. Fan , W. Yang , L. Zhang , H. Cai , Y. Zhuang , Y. Chen , Y. Zhao , J. Dai , Biomaterials 2022, 288, 121689. 35931574 10.1016/j.biomaterials.2022.121689

[74] J. Xu , T. Chen , C. Tai , S. Hsu , Biomater. Res. 2023, 27, 8. 36755333 10.1186/s40824-023-00347-0 PMC9909866

[75] J. Xu , C. Tai , T. Chen , S. Hsu , Chem. Eng. J. 2022, 446, 137180.

[76] D. Wei , M. Zeng , B. Su , Y. Zhang , J. Ding , C. Wu , J. Sun , L. Zhou , H. Yin , H. Fan , Chem. Eng. J. 2024, 484, 149521.

[77] J. Xu , C. Wong , S. Hsu , Chem. Mater. 2020, 32, 10407.

[78] Z. Yang , Y. You , X. Liu , Q. Wan , Z. Xu , Y. Shuai , J. Wang , T. Guo , J. Hu , J. Lv , M. Zhang , M. Yang , C. Mao , S. Yang , J. Nanobiotechnol. 2024, 22, 111. 10.1186/s12951-024-02359-x PMC10941401 38486273

[79] Y. Zhang , M. Zhang , R. Zhang , H. Liu , H. Chen , X. Zhang , C. L. , Q. Zeng , Y. Chen , G. Huang , Front. Mater. 2022, 9.

[80] J. Wang , X. Li , Y. Song , Q. Su , X. Xiaohalati , W. Yang , L. Xu , B. Cai , G. Wang , Z. Wang , L. Wang , Bioactive Materials 2021, 6, 1988. 33474513 10.1016/j.bioactmat.2020.12.017 PMC7786039

[81] M. Dong , X. Wang , X.‐Z. Chen , F. Mushtaq , S. Deng , C. Zhu , H. Torlakcik , A. Terzopoulou , X.‐H. Qin , X. Xiao , J. Puigmart‐Luis , H. Choi , A. P. Pêgo , Q.‐D. Shen , B. J. Nelson , S. Pané , Adv. Funct. Mater. 2020, 30, 1910323.

[82] S. Ghosh , M. Dhiman , S. Gupta , P. Roy , D. Lahiri , Biomater. Adv. 2023, 154, 213596. 37672898 10.1016/j.bioadv.2023.213596

[83] D. Xu , F. Siqi , Z. Hui , L. Weicheng , X. Jingdun , L. Jilai , W. Huan , Z. Yuanjin , C. Renjie , Adv. Mater. 2024, 36, 2307896.

[84] Y. Jin , W. Zhang , Y. Zhang , Y. Yang , Z. Fang , J. Song , Y. Qian , W.‐E. Yuan , Biomater. Adv. 2022, 135, 212727. 35929199 10.1016/j.bioadv.2022.212727

[85] Y. Hu , Z. Chen , H. Wang , J. Guo , J. Cai , X. Chen , H. Wei , J. Qi , Q. Wang , H. Liu , Y. Zhao , R. Chai , ACS Nano 2022, 16, 1868. 35112853 10.1021/acsnano.1c11627

[86] E. Hoveizi , Biomed. Mater. 2023, 19, 015012. 10.1088/1748-605X/ad1576 38091624

[87] A. Soltani Khaboushan , A. Azimzadeh , S. Behboodi Tanourlouee , M. Mamdoohi , A.‐M. Kajbafzadeh , K. V. Slavin , V. Rahimi‐Movaghar , Z. Hassannejad , Sci. Rep. 2024, 14, 15196. 38956215 10.1038/s41598-024-65286-9 PMC11219763

[88] J. Park , J. Jeon , B. Kim , M. S. Lee , S. Park , J. Lim , J. Yi , H. Lee , H. S. Yang , J. Y. Lee , Adv. Funct. Mater. 2020, 30, 2003759.

[89] Q. Huang , Y. Cai , X. Yang , W. Li , H. Pu , Z. Liu , H. Liu , M. Tamtaji , F. Xu , L. Sheng , T.‐H. Kim , S. Zhao , D. Sun , J. Qin , Z. Luo , X. Lu , Nano Res. 2022, 15, 3434.

[90] Y. Gao , C. Dai , M. Zhang , J. Zhang , L. Yin , W. Li , K. Zhang , Y. Yang , Y. Zhao , Adv. Funct. Mater. 2024, 34, 2314610.

[91] C. Xu , P. Wu , K. Yang , C. Mu , B. Li , X. Li , Z. Wang , Z. Liu , X. Wang , Z. Luo , Small 2024, 20, 2309793 10.1002/smll.202309793 38148305

[92] Y. Han , M. Sun , X. Lu , K. Xu , M. Yu , H. Yang , J. Yin , Composites, Part B 2024, 273, 111241.

[93] S. Liu , B. Chen , Y. Feng , C. Gao , D. Du , T. Jiang , Y. Tu , F. Peng , Chem. Eng. J. 2024, 479, 147745.

[94] S. Wang , Z. Wang , W. Yang , Z. Xu , H. Dai , F. He , S. Yan , X. Shi , Adv. Mater. 2024, 36, 2311264. 10.1002/adma.202311264 38330187

[95] B. Xia , X. Gao , J. Qian , S. Li , B. Yu , Y. Hao , B. Wei , T. Ma , H. Wu , S. Yang , Y. Zheng , X. Gao , L. Guo , J. Gao , Y. Yang , Y. Zhang , Y. Wei , B. Xue , Y. Jin , Z. Luo , J. Zhang , J. Huang , Adv. Mater. 2024, 36, 2305374. 10.1002/adma.202305374 37652460

[96] L. Wang , P. Dang , H. Zheng , L. Wei , S. Jiang , J. Wang , Y. Cai , W. Wang , C. Zhang , N. Li , J. Xia , Colloids Surf. Physicochem. Eng. Asp. 2024, 701, 134822.

[97] M. Antman‐Passig , J. Giron , M. Karni , M. Motiei , H. Schori , O. Shefi , Adv. Funct. Mater. 2021, 31, 2010837.

[98] M. M. Pillai , G. Sathishkumar , S. Houshyar , R. Senthilkumar , A. Quigley , S. Shanthakumari , R. Padhye , A. Bhattacharyya , ACS Appl. Bio Mater. 2020, 3, 4454. 10.1021/acsabm.0c00430 35025444

[99] L. Huang , X. Yang , L. Deng , D. Ying , A. Lu , L. Zhang , A. Yu , B. Duan , ACS Appl. Mater. Interfaces 2021, 13, 16106. 33787211 10.1021/acsami.1c01904

[100] M. Kasper , B. Ellenbogen , R. Hardy , M. Cydis , J. Mojica‐Santiago , A. Afridi , B. S. Spearman , I. Singh , C. A. Kuliasha , E. Atkinson , K. J. Otto , J. W. Judy , C. Rinaldi‐Ramos , C. E. Schmidt , Biomaterials 2021, 279, 121212. 34717196 10.1016/j.biomaterials.2021.121212 PMC9036633

[101] Y. Yang , X. Yin , H. Wang , W. Qiu , L. Li , F. Li , Y. Shan , Z. Zhao , Z. Li , J. Guo , J. Zhang , Y. Zhao , Nano Energy 2023 107, 108145.

[102] F. Delavar , M. Mohseni , A. Jahandideh , M. Khajehmohammadi , N. Najmoddin , Int. J. Biol. Macromol. 2025, 286, 137833. 39566755 10.1016/j.ijbiomac.2024.137833

[103] K. Li , D. Ye , Z. An , J. Xu , X. Sun , M. Liu , P. Li , Mater. Today Commun. 2024, 39, 109120.

[104] S. Das , J. Jegadeesan , B. Basu , ACS Biomater. Sci. Eng. 2024, 10, 1620. 38345020 10.1021/acsbiomaterials.3c01226

[105] J. Amagat , Y. Su , F. H. Svejsø , A. L. Friec , S. M. Sønderskov , M. Dong , Y. Fang , M. Chen , Mater. Today Bio 2022, 16, 100437. 10.1016/j.mtbio.2022.100437 PMC9526217 36193343

[106] F. Zheng , R. Li , Q. He , K. Koral , J. Tao , L. Fan , R. Xiang , J. Ma , N. Wang , Y. Yin , Z. Huang , P. Xu , H. Xu , Mater. Sci. Eng. C Mater. Biol. Appl. 2020, 109, 110560. 32228996 10.1016/j.msec.2019.110560

[107] M. Xu , Q. Li , Z. Fang , M. Jin , Q. Zeng , G. Huang , Y.‐G. Jia , L. Wang , Y. Chen , Biomater. Sci. 2020, 8, 6957. 33103177 10.1039/d0bm01466d

[108] Z. Qiao , J. Ding , M. Yang , Y. Wang , T. Zhou , Y. Tian , M. Zeng , C. Wu , D. Wei , J. Sun , H. Fan , Acta Biomater. 2024, 184, 114. 38942188 10.1016/j.actbio.2024.06.028

[109] M.‐H. Tan , X.‐H. Xu , T.‐J. Yuan , X. Hou , J. Wang , Z.‐H. Jiang , L.‐H. Peng , Biomaterials 2022, 283, 121413. 35276616 10.1016/j.biomaterials.2022.121413

[110] C. Zhang , T.‐J. Yuan , M.‐H. Tan , X.‐H. Xu , Y.‐F. Huang , L.‐H. Peng , Biomater. Sci. 2021, 9, 2146. 33496688 10.1039/d0bm01963a

[111] L. Jiang , X. Wu , Y. Wang , C. Liu , Y. Wu , J. Wang , N. Xu , Z. He , S. Wang , H. Zhang , X. Wang , X. Lu , Q. Tan , X. Sun , Adv. Sci. 2023, 10, 2300339. 10.1002/advs.202300339 PMC10369251 37148168

[112] L. Fan , C. Xiao , P. Guan , Y. Zou , H. Wen , C. Liu , Y. Luo , G. Tan , Q. Wang , Y. Li , P. Yu , L. Zhou , C. Ning , Adv. Healthcare Mater. 2022, 11, 2101556. 10.1002/adhm.202101556 34648694

[113] H. Wang , Y.‐C. Hsu , C. Wang , X. Xiao , Z. Yuan , Y. Zhu , D. Yang , ACS Appl. Mater. Interfaces 2024, 16, 17208. 38530974 10.1021/acsami.3c19410

[114] X. Zhang , H. Zhang , Y. Zhang , H. Huangfu , Y. Yang , Q. Qin , Y. Zhang , Y. Zhou , J. Mater. Chem. B 2023, 11, 1288. 36651822 10.1039/d2tb01979e

[115] Y. Xu , C. Xu , K. Yang , L. Ma , G. Li , Y. Shi , X. Feng , L. Tan , D. Duan , Z. Luo , C. Yang , Adv. Healthcare Mater. 2023, 12, 2301151. 10.1002/adhm.202301151 37421228

[116] X. Jing , C. Xu , W. Su , Q. Ding , B. Ye , Y. Su , K. Yu , L. Zeng , X. Yang , Y. Qu , K. Chen , T. Sun , Z. Luo , X. Guo , Adv. Healthcare Mater. 2023, 12, 2201349. 10.1002/adhm.202201349 36325633

[117] H. Zheng , Z. Yang , L. Zhou , B. Zhang , R. Cheng , Q. Zhang , Small 2024, 20, 2310483. 10.1002/smll.202310483 39254284

[118] Z. Zhang , S. Gao , Y.‐N. Hu , X. Chen , C. Cheng , X.‐L. Fu , S.‐S. Zhang , X.‐L. Wang , Y.‐W. Che , C. Zhang , R.‐J. Chai , Adv. Sci. 2022, 9, 2203557. 10.1002/advs.202203557 PMC9661825 36117048

[119] Y. Hu , W. Chen , H. Yin , X. Chen , J. Cai , J. Guo , S. Zhou , R. Chai , M. Tang , Materials Today Nano 2022, 18, 100181.

[120] E. Lacroce , G. Perale , F. Rossi , in Diagnosis and Treatment of Spinal Cord Injury, (Eds.: R. Rajendram , V. R. Preedy , C. R. Martin ), Academic Press, USA 2022, p. 549.

[121] S. Aqel , N. Al‐Thani , M. Z. Haider , S. Abdelhady , A. A‐Al‐Thani , F. Kobeissy , A. A Shaito , Biology 2023, 13, 21. 38248452 10.3390/biology13010021 PMC10813103

[122] Functional evaluation of peripheral nerve regeneration and target reinnervation in animal models: a critical overview, PubMed https://pubmed.ncbi.nlm.nih.gov/26228942/ (accessed: July 2025). 10.1111/ejn.13033 26228942

[123] B. Lopes , B. Lopes , P. Sousa , R. Alvites , M. Branquinho , A. C. Sousa , C. Mendonça , L. M. Atayde , A. L. Luís , A. S. P. Varejão , A. Ce Maurício , Int. J. Mol. Sci. 2022, 23, 918. 35055104 10.3390/ijms23020918 PMC8779751

[124] A. P. W. Johnston , F. D. Miller , Cold Spring Harb. Perspect. Biol. 2022, 14, a041233. 35667791 10.1101/cshperspect.a041233 PMC9438784

[125] T. K. Gupta , P. R. Budarapu , S. R. Chappidi , S. S. YB , M. Paggi , S. P. Bordas , Curr. Med. Chem. 2019, 26, 6851. 30474523 10.2174/0929867326666181126113605

[126] W. B. Han , S. M. Yang , K. Rajaram , S.‐W. Hwang , Adv. Sustain. Syst. 2022, 6, 2100075.

[127] I. A. Vasyukova , O. V. Zakharova , D. V. Kuznetsov , A. A. Gusev , Nanomaterials 2022, 12, 1797. 35683652 10.3390/nano12111797 PMC9182201

[128] M. van Druenen , Adv. Mater. Interfaces 2020, 7, 2001102.

[129] G. G. Genchi , A. Marino , A. Rocca , V. Mattoli , G. Ciofani , Nanotechnology 2016, 27, 232001. 27145888 10.1088/0957-4484/27/23/232001

[130] A. Górska , M. Trubalski , B. Borowski , A. Brachet , S. Szymańczyk , R. Markiewicz , Frontiers in Cell and Developmental Biology 2024, 12, 1435461. 39588275 10.3389/fcell.2024.1435461 PMC11586186

[131] A. Napoli , I. Obeid , J. Cell. Biochem. 2016, 117, 559. 26284690 10.1002/jcb.25312

[132] D. B. Pazzin , T. T. R. Previato , J. I. Budelon Gonçalves , G. Zanirati , F. A. C. Xavier , J. C. da Costa , D. R. Marinowic , Cells 2024, 13, 745. 38727281 10.3390/cells13090745 PMC11083827

[133] T. Sen , R. P. Thummer , Neurotox. Res 2022, 40, 1597. 36044181 10.1007/s12640-022-00564-w PMC9428373

[134] Q.‐M. Pang , S.‐Y. Chen , Q.‐J. Xu , S.‐P. Fu , Y.‐C. Yang , W.‐H. Zou , M. Zhang , J. Liu , W.‐H. Wan , J.‐C. Peng , T. Zhang , Front. Immunol. 2021, 12, 751021. 34925326 10.3389/fimmu.2021.751021 PMC8674561

[135] M. Gulino , D. Kim , S. Pané , S. D. Santos , A. P. Pêgo , Front. Neurosci. 2019, 13, 689. 31333407 10.3389/fnins.2019.00689 PMC6624471

[136] P. Kumar , D. Zelena , A. Gautam , Neurological Disorders and Challenges in Their Theranostics (eds. A. Gautam , V. Chaudhary ), Springer Nature, Berlin, Germany 2024, pp. 1–29.

[137] D. W. Dickson , Parkinsonism Relat. Disord. 2018, 46, S30. 28780180 10.1016/j.parkreldis.2017.07.033 PMC5718208

[138] L. Wu , X. Xiong , X. Wu , Y. Ye , Z. Jian , Z. Zhi , L. Gu , Nat. Rev. Drug Discovery 2021, 20, 689 34194012

[139] A. Bonura , N. Brunelli , M. Marcosano , G. Iaccarino , L. Fofi , F. Vernieri , C. Altamura , Int. J. Mol. Sci. 2023, 24, 13979. 37762283 10.3390/ijms241813979 PMC10530509

