# Supplementary material for: Conductive Nanocomposite Hydrogels for Neural Tissue Engineering: A Systematic Scoping Review of Recent Trends
Source: Adv Sci (Weinh). 2025 Sep 8;12(38):e16085. doi: 10.1002/advs.202416085 (PMC12520499; doi:10.1002/advs.202416085)
Supplement: Supplementary file 1 — Supporting Information [file ADVS-12-e16085-s001.docx]

Supporting Information

**CONDUCTIVE NANOCOMPOSITE HYDROGELS FOR NEURAL TISSUE ENGINEERING: A SYSTEMATIC SCOPING REVIEW OF RECENT TRENDS**

*Mohammad Moghaddasi^1^, Busra Oktay^1^, Ayse Betul Bingol^1^, Reyhan Yanikoglu^1^,Meryem Muslu^2^ , Ibrahim T Ozbolat^4^, Cem Bulent Ustundağ^3^**

- 1. **List of Abbreviations**

**Fig.4-C**

- CNT: Carbon Nanotubes
- GO: Graphene Oxide
- rGO: Reduced Graphene Oxide
- PEDOT: Poly(3,4-ethylenedioxythiophene)
- Ppy: Polypyrrole
- PEI: Polyethyleneimine
- BP: Black Phosphorus
- GeP: Germanium Phosphide
- SiP: Silicon Phosphide
- CoFe_2_O_4_: Cobalt Ferrite
- BaTiO_3_: Barium Titanate
- BiFeO_3_: Bismuth Ferrite

**Fig.4-D**

- NSCs: Neural Stem Cells
- MSCs: Mesenchymal Stem Cells
- ADSCs: Adipose-Derived Stem Cells
- PC12: Pheochromocytoma Cells
- SH-SY5Y: Human Neuroblastoma Cells
- C2C12: Mouse Myoblast Cells
- Neuro2a: Mouse Neuroblastoma Cells
- RSC-96: Rat Schwann Cells
- NPCs: Neural Progenitor Cells
- NE-4C: Neuroepithelial Cells

**Table 2**

PVA, polyvinyl alcohol; HA, hyaluronic acid; PEG, polyethylene glycol; PEGDA, polyethylene glycol diacrylate; Au, gold; GO, graphene oxide; MoS₂, molybdenum disulfide; AuNPs, gold nanoparticles; BaTiO₃, barium titanate; rGO, reduced graphene oxide; 0-D, zero-dimensional nanomaterials; 1-D, one-dimensional nanomaterials; 2-D, two-dimensional nanomaterials; h, hour(s); NSCs, neural stem cells; MSCs, mesenchymal stem cells; BM-MSCs, bone marrow-derived mesenchymal stem cells; PC12, rat pheochromocytoma cells; BP, black phosphorus; PPM, Photopolymerization; OP1, Option 1 = Suspending Pre-Assembled Nanomaterial Prior to Hydrogel Crosslinking; OP2, Option 2 = Hydrogel Crosslinking Followed by In Situ Nanomaterial Formation; OP3, Option 3 = Hydrogel Crosslinking Followed by Inward Diffusion of Pre-Assembled Nanomaterial; OP4, Option 4 = Co-Dependent Hydrogel-Nanomaterial Assembly; C, Only the Conductive Material; C+E, Conductive Hydrogel + Electrical Stimulation; C+N, Conductive Hydrogel + Neurogenic Differentiation Medium; C+N+E, Conductive Hydrogel + Electrical Stimulation + Neurogenic Differentiation Medium; EFS, Electric Field Strength; MWS, Magnetic Wireless Stimulation; DCS, Direct Current Stimulation; ACS, Alternating Current Stimulation; UWS,Ultrasound Wireless Stimulation; WS, Wireless Stimulation; PW, Pulse Width; PA, Pulse Amplitude; f, Frequency; FS, Field Strength; I, Intensity.

**Table 3**

HT-22, Mouse Hippocampal Neuronal Cells; SH-SY5Y, Human Neuroblastoma Cells; NSCs, Neural Stem Cells; MSCs, Mesenchymal Stem Cells; BP, Black Phosphorus; 0-D, zero-dimensional nanomaterials; 1-D, one-dimensional nanomaterials; 2-D, two-dimensional nanomaterials; h, hour(s); PPM, Photopolymerization; OP1, Option 1 = Suspending Pre-Assembled Nanomaterial Prior to Hydrogel Crosslinking; OP2, Option 2 = Hydrogel Crosslinking Followed by In Situ Nanomaterial Formation; OP3, Option 3 = Hydrogel Crosslinking Followed by Inward Diffusion of Pre-Assembled Nanomaterial; OP4, Option 4 = Co-Dependent Hydrogel-Nanomaterial Assembly; C, Only the Conductive Material; C+E, Conductive Hydrogel + Electrical Stimulation; C+N, Conductive Hydrogel + Neurogenic Differentiation Medium; C+N+E, Conductive Hydrogel + Electrical Stimulation + Neurogenic Differentiation Medium; EFS, Electric Field Strength; MWS, Magnetic Wireless Stimulation; DCS, Direct Current Stimulation; WS, Wireless Stimulation; f, Frequency; FS, Field Strength.

**Table 4**

NIPAM, Poly(N-isopropylacrylamide); PAAm, Polyacrylamide; PEI, Polyethylenimine; PLLA, Poly(L-lactic acid); MWCNTs, Multi-Walled Carbon Nanotubes; g-C3N4, Graphitic Carbon Nitride; RSC-96, Rat Schwann Cells; Neuro2a, Mouse Neuroblastoma Cells; ADSC, Adipose-Derived Stem Cells; PC12, Rat pheochromocytoma cells; 0-D, zero-dimensional nanomaterials; 1-D, one-dimensional nanomaterials; 2-D, two-dimensional nanomaterials; h, hour(s); PPM, Photopolymerization; OP1, Option 1 = Suspending Pre-Assembled Nanomaterial Prior to Hydrogel Crosslinking; OP2, Option 2 = Hydrogel Crosslinking Followed by In Situ Nanomaterial Formation; OP3, Option 3 = Hydrogel Crosslinking Followed by Inward Diffusion of Pre-Assembled Nanomaterial; OP4, Option 4 = Co-Dependent Hydrogel-Nanomaterial Assembly; C, Only the Conductive Material; C+E, Conductive Hydrogel + Electrical Stimulation; C+N, Conductive Hydrogel + Neurogenic Differentiation Medium; C+N+E, Conductive Hydrogel + Electrical Stimulation + Neurogenic Differentiation Medium; EFS, Electric Field Strength; MWS, Magnetic Wireless Stimulation; DCS, Direct Current Stimulation; ACS, Alternating Current Stimulation; PES, Pulsed Electrical Stimulation; WS, Wireless Stimulation; PW, Pulse Width; PA, Pulse Amplitude; f, Frequency; FS, Field Strength; I, Intensity.

**Table 5**

Bi_2_S_3_, Bismuth Sulfide; PEI, Polyethylenimine; GO, Graphene Oxide; rGO, Reduced Graphene Oxide; HA, Hyaluronic Acid; PAAm, Polyacrylamide; GelMA, Gelatin Methacryloyl; MSCs, Mesenchymal Stem Cells; PC12, Rat Pheochromocytoma Cells; NSCs, Neural Stem Cells; C2C12, Mouse Myoblast Cells; NIH-3T3, Mouse Embryonic Fibroblast Cells; NE-4C, Mouse Neuroectodermal Stem Cells; 0-D, Zero-Dimensional Nanomaterials; 1-D, One-Dimensional Nanomaterials; 2-D, Two-Dimensional Nanomaterials; h, Hour(s); PPM, Photopolymerization; OP1, Option 1 = Suspending Pre-Assembled Nanomaterial Prior to Hydrogel Crosslinking; OP2, Option 2 = Hydrogel Crosslinking Followed by In Situ Nanomaterial Formation; OP3, Option 3 = Hydrogel Crosslinking Followed by Inward Diffusion of Pre-Assembled Nanomaterial; OP4, Option 4 = Co-Dependent Hydrogel-Nanomaterial Assembly; C, Only the Conductive Material; C+E, Conductive Hydrogel + Electrical Stimulation; C+N, Conductive Hydrogel + Neurogenic Differentiation Medium; C+N+E, Conductive Hydrogel + Electrical Stimulation + Neurogenic Differentiation Medium; OWS, Optical Wireless Stimulation;

**1.2 Data Extraction Form**

1.1 Is the study included or excluded?

- Included
- Excluded
  1. What is the origin of the Neural cells in the scaffold?
- Human
- Animal
- Both
- Unclear

2.2 What is the main type of neural cell utilized?

- Stem cells
- Progenitor cells
- Induced pluripotent stem cells (iPSCs)
- Cell line
- Other

2.2.1 If cell line ,which one was utilized?

- PC12 (Pheochromocytoma 12**)**
- Neuro2a (Neuroblastoma 2A)
- SH-SY5Y(Human neuroblastoma cell line)
- U87-MG (Human glioblastoma cell line)
- C2C12(Mouse myoblast cell line)
- NH-3T3(Mouse fibroblast cell line)
- HT-22(Mouse hippocampal cell line)
- RSC96(Rat Schwann cell line)
- Other

2.3 What is the mode of culture?

- Mono-culture
- Co-culture
- Unclear

2.3.1 If co-culture, which is the type of the secondary cells?

- Glial Cells (Astrocytes, Microglia, Oligodendrocytes)
- Endothelial Cells
- Fibroblasts
- Immune cells
- Other

2.4 Is the cell seeding density onto the hydrogel reported?

- Yes
- No
- Unclear

2.4.1 If yes, Insert Cell density.

[ ___________ ]

2.4.2 Insert Cell density Unit

[ ___________ ]

2.5 How long cells were cultured within the hydrogel before in vitro cell characterization?

- < 3 days (< 3d)
- 3 days-2 weeks (3d-2w)
- 2 weeks- 3months (2w-3m)
- 3 months-1 year (3m-1y)
- >1year (> 1y)
- Not reported

3.1 What is the origin of the biomaterial used as the backbone of the hydrogel?

- Natural
- Synthetic
- Mixed (specify)
- Unclear
- Not reported

3.1.1 If natural biomaterial, which sub-type is used?

- Protein-based
- Polysaccharide-based
- Decellularized Extracellular Matrix (dECM)
- Other
- Mixed (Specify)
- Unclear

3.1.1.1 If Protein-based, which one?

- Collagen
- Fibrinogen / Fibrin
- Silk
- Other
- Unclear

3.1.1.2 If Polysaccharide-based, which one?

- Alginate
- Gelatin
- Gellan Gum
- Hyaluronic acid (HA)
- Dextran
- Agarose
- Chitosan
- Cellulose
- Other
- Unlcear

3.1.1.3 If Mixed, specify:

[_____________]

3.1.2 If Synthetic biomaterial, which subtype?

- Polyethylene Glycol (PEG)
- Poly(lactic-co-glycolic acid) (PLGA)
- Polyvinyl Alcohol (PVA)
- Polylactic Acid (PLA)
- Polycaprolactone (PCL)
- Poly(2-hydroxyethyl methacrylate) (PHEMA)
- Polyacrylic Acid (PAA)
- Polydimethylsiloxane (PDMS)
- Polyurethane
- Polyacrylates/Polyacrylamides
- Other
- Unclear

3.2 What is the fabrication method?

- Crosslinking
- Self-assembly
- 3D bioprinting
- Other
- Unclear

3.2.1 If crosslinking, which one?

- Covalent
- Physical
- Ionic
- Photopolymerization
- Thermal gelation
- Other
- Unclear

3.2 .2 If 3D bioprinting, which method?

- Extrusion-based
- Inkjet-based
- Laser-assisted
- Stereolithography (SLA)
- Digital Light Processing (DLP)
- Combination
- Other
- Unclear

3.2.3 If self-assembly,which one?

- Peptide-Based
- DNA-based
- Polysaccharide-based
- Block Copolymer-based
- Other
- Unclear

3.3 What is the geometry of the hydrogel synthesized?

- Injectable
- film/sheet
- conduit
- General 3D structure
- Other
- Unclear

4.1 What type of nanomaterial is used inside the hydrogel structure?

- Carbon-based
- Metal-Based
- Ceramic-Based
- Polymer-Based
- Semiconductor-Based
- Other
- Mixed (Specify)

4.1.1 If carbon based, which one?

- Graphene oxide (GO)
- Reduced Graphene Oxide (rGo)
- Graphene
- Carbon nanotube (Single/Multi Walled) (CNT)
- Carbon nanodots
- Other

4.1.2 If metal-based, which one?

- Gold (AuNPs)
- Silver (AgNPs)
- Iron (FeNPs)
- Platinum (PtNPs)
- Mxene
- Other

4.1.3 If Ceramic-Based, which one ?

- Cobalt Ferrite (CoFe2​O4​)
- Barium Titanate (BaTiO3)
- Zirconium-based
- Other

4.1.4 If polymer-Based, which one?

- polypyrrole (Ppy)
- polyaniline (PANI)
- poly(3,4-ethylenedioxythiophene) (PEDOT)
- polythiophene (PEI)
- Other

4.1.5 If Semiconductor-Based, which one?

- Black Phosphorus (BP)
- Silicon-Based
- Germanium-based
- Titanium-Based
- Other

4.1.6 If Mixed, Specify.

[____________]

4.2 How was the nanostructure integrated with the hydrogel?

- Suspending pre-assembled nanomaterial prior to hydrogel crosslinking (OP1)
- Hydrogel crosslinking followed by in situ nanomaterial formation (OP2)
- Hydrogel crosslinking followed by inward diffusion of pre-assembled nanomaterial (OP3)
- Co-dependent hydrogel- nanomaterial assembly (OP4)
- Other
- Unclear

4.3 What is the dimension of the conductive nanoparticle integrated?

- Zero-dimensional (e.g. nanoparticle) (0D)
- One-dimensional (e.g. nanowires, nanotubes, etc) (1D)
- Two-dimensional (e.g. nanoplates, nanosheets, etc.) (2D)
- Mixed (specify)
- Unclear

5.1 Which in vitro quality-assuring aspects were studied for the neural tissue models?

- Cell viability (Specify method)
- Cell proliferation (Specify method)
- Cell adhesion
- Cell migration (Specify method)
- Neural Gene Expression Analysis (e.g. via RT-qPC)
- Neural Protein Expression Analysis (e.g. via Immunocytochemistry)

5.2 Which in vitro quality-assuring aspects were studied for the hydrogel?

- Degradation test
- Rheological test
- Porosity
- Swelling test
- Mechanical tests
- electrical properties

5.2.1 If mechanical/ rheological tests were performed, was there a comparison between conductive hydrogel’s relevant properties with that of pure hydrogel?

- Yes
- No
- Unclear

5.2.1.1 If yes, what parameter(s) was used to compare them?

- Elastic / Young modulus
- Compression/ Bulk modulus
- Storage or loss modulus (G’/G’’)
- Tensile strength /strain
- Unclear
- Other

5.2.2 If the Electrical property of the nanocomposite hydrogel was measured, which approach(s) was used?

- Conductivity measurements (Specify method)
- Impedance-based methods (Specify method)
- Electrochemical methods (Specify method)
- Unclear
- Other

5.2.2.1 Insert the highest conductivity reported in S/cm

[____________]

6.1 What is the type of neural tissue intended to be modeled/regenerated?

- Peripheral nervous system ( Motor, Sensory, Autonomic nerves) (PNS, specify area)
- Central nervous system (Brain, Spinal Cord tissue) (CNS, Specify area)
- Neural organoids
- Mixed
- Other
- Unclear

6.2 For neural cell differentiation, which condition(s) were applied?

- Conductive hydrogel + Electrical stimulation (C + E)
- Conductive hydrogel + Neurogenic Differentiation medium (C + N)
- Conductive hydrogel + Electrical stimulation + Neurogenic Differentiation medium ( C +N +E)
- Only the conductive material (C)

6.2.1 If electrical stimulation was performed, which type of electrical stimulation was applied?

- - Continuous Stimulation (DC)
  - Alternating Stimulation (AC)
  - Wireless electrical stimulation (Specify)
  - Combination
  - Unclear

6.2.1.1 For continuous stimulation, if reported, insert the strength of the electrical field applied in mV/cm:

[________________]

6.2.1.2 For Alternating stimulation, if reported, insert the Pulse width in milliseconds (ms):

[________________]

6.2.1.3 For Alternating stimulation, if reported, insert the Pulse Frequency in Hertz(Hz)

[________________]

6.2.1.4 For Alternating stimulation, if reported, insert the Pulse Amplitude

[________________]

6.2.1.4.1 insert the Pulse Amplitude unit

[________________]

6.2.1.5 For Magnetic wireless electrical stimulation, insert the magnetic field strength

In milliTesla (mT)

[________________]

6.2.1.6 For Magnetic wireless electrical stimulation, insert the magnetic field frequency in Hz

[________________]

6.2.1.7 For other types of wireless stimulation, insert method name along parameters with units

[________________]

6.2.2 if electrical stimulation was performed, if reported, insert the number of total hours stimulation was performed.

[________________]

6.2.2.1 electrical stimulation happened over a span of how many days?

[________________]

6.2.3 If a Neurogenic differentiation medium was utilized, what type(s) of neurotrophic factor did it contain?

- Basic Fibroblast Growth Factor (bFGF)
- Glial Cell-Derived Neurotrophic Factor (GDNF)
- Brain-Derived Neurotrophic Factor (BDNF)
- Vascular Endothelial Growth Factor (VEGF)
- retinoic acid (RA)
- Mixed (Specify)
- Other
- Not reported

6.2.3.1 If reported, Insert the duration of incubation of the cell-laden hydrogel inside this medium. (in days)

[________________]

6.2.3.2 IF reported, insert the concentration amount with the Unit.

[________________]

- 1. Which approach(s) was used to study the neural differentiation profile?
- Morphology Screening (e.g neurite growth, synaptic connectivity, somatic size, etc)
- Electrophysiological assessment (e.g action potential generation, synaptic activity)
- Neural gene marker analysis
- Neural protein marker analysis
- Other
- Unclear

6.3.1 If Morphology screening was performed, Which method(s) was used?

- Neurite Growth/Length
- Synapsis formation/connectivity
- Somatic size
- Other
- Unclear

6.3.2 If Electrophysiological Assessment was performed, Which aspect(s) was studied?

- Action Potential Generation
- Synaptic Activity
- Membrane Properties
- Neurotransmitter release
- Other
- Unclear

6.3.3 If Genetic marker analysis was performed, Which method(s) was used?

- PCR
- RNA sequencing (RNA-seq)
- In situ Hybridization (ISH)
- Microarray Analysis
  - Other
  - Unclear

6.3.3.1 Genetic material related to which group was analyzed?

- Neural Stem Cells
- Neuronal Cells and Function
- Oligodendrocytes
- Astrocytes
- Neurotrophic Factors
- Unclear
- Other

6.3.3.1.1 if Neural stem cell, genetic material for which marker?

- Nestin
- SRY-Box Transcription Factor 2 (Sox2)
- CD133
- Other

6.3.3.1.2 if Neuronal cells and function, genetic material for which marker?

- Beta-Tubulin III (TUJ1)
- Microtubule-Associated Protein 2 (MAP2)
- Neuronal Nuclei (NeuN)
- Synaptophysin (SYP)
- Neurofilament Chain (NF)
- Tau
- L-Type Voltage-Gated Ca2+ Channel (Cav1.2)
- Calcium Channel, Voltage-Gated, Beta 3 Subunit (Cacnb3)
- PSD95 (postsynaptic density protein 95)
- SYN (Synapsin)
- Other

6.3.3.1.3 if Oligodendrocytes, genetic material for which marker?

- Oligodendrocyte Transcription Factor 2 (Olig2)
- 2',3'-Cyclic Nucleotide 3'-Phosphodiesterase (CNPase)
- Myelin Basic Protein (MBP)
- Oligodendrocyte Marker 4 (O4)
- Other

6.3.3.1.4 if Astrocytes, genetic material for which marker?

- Glial Fibrillary Acidic Protein (GFAP)
- S100 Protein
- Aquaporin 4 (AQP4)
- Other

6.3.3.1.5 if Neurotrophic factors, genetic material for which marker?

- - - Brain-Derived Neurotrophic Factor (BDNF)
    - Neurotrophin-3 (NT-3)
    - Nerve Growth Factor (NGF)
    - Other

6.3.4 If neural protein marker analysis was performed, Which method(s) was used?

- Western Blot
- ELISA (Enzyme-Linked Immunosorbent Assay)
- Immunostaining
- Mass Spectrometry (MS)
- Flow Cytometry
- Unclear
- Other

6.3.4.1 which group(s) of neural proteins was analyzed?

- - Neural Stem Cells
  - Neuronal Cells and Function
  - Oligodendrocytes
  - Astrocytes
  - Neurotrophic Factors
  - Unclear
  - Other

6.3.4.1.1 if Neural stem cell, which protein?

- Nestin
- SRY-Box Transcription Factor 2 (Sox2)
- CD133
- Other

6.3.4.1.2 If Neuronal cells and function, which protein?

- - - - Beta-Tubulin III (TUJ1)
      - Microtubule-Associated Protein 2 (MAP2)
      - Neuronal Nuclei (NeuN)
      - Synaptophysin (SYP)
      - Neurofilament Chain (NF)
      - Tau
      - L-Type Voltage-Gated Ca2+ Channel (Cav1.2)
      - Calcium Channel, Voltage-Gated, Beta 3 Subunit (Cacnb3)
      - PSD95 (postsynaptic density protein 95)
      - SYN (Synapsin)
      - Other

6.3.4.1.3 If Oligodendrocytes, which protein?

- Oligodendrocyte Transcription Factor 2 (Olig2)
- 2',3'-Cyclic Nucleotide 3'-Phosphodiesterase (CNPase)
- Myelin Basic Protein (MBP)
- Other

6.3.4.1.4 f Astrocytes, which protein?

- Glial Fibrillary Acidic Protein (GFAP)
- S100 Protein
- Aquaporin 4 (AQP4)
- Other

6.3.4.1.5 if Neurotrophic factors, which protein?

- - - Brain-Derived Neurotrophic Factor (BDNF)
    - Neurotrophin-3 (NT-3)
    - Nerve Growth Factor (NGF)
    - Other
  1. Was in vivo testing performed for the developed neural model?
- Yes
- No

7.1.1 If yes which approach(s) was used to assess the effectiveness of the model in regenerating defect neural tissue?

- Histological Analysis
- Behavioral Testing
- Electrophysiological Measurements
- Protein Marker Analysis
- Gene Marker Analysis
- Other
- Unclear

7.1.1.1 If Behavioral Testing was performed, which functional aspect(s) was assessed?

- - - Motor Function (Specify)
    - Sensory Function (Specify)
    - Cognitive Ability (Specify)
    - Emotional or Social Behavior (Specify)
    - Other (Specify)
    - Unclear

7.1.1.2 If Electrophysiological Measurements were performed, which parameter(s) was analyzed?

- Local Field Potential (LFP)
- Single-Unit Recording
- Multi-Unit Recording
- EEG or MEG Signals
- Other
- Unclear

7.1.1.3 If Protein Marker Analysis was performed Insert the protein markers analyzed during in vivo study.

[______________________]
